# In the pursuit of novel therapeutic agents: synthesis, anticancer evaluation, and physicochemical insights of novel pyrimidine-based 2-aminobenzothiazole derivatives†

**DOI:** 10.1039/d4ra01874e

**Published:** 2024-05-20

**Authors:** Toka I. Ismail, Nashwa El-Khazragy, Rasha A. Azzam

**Affiliations:** a Chemistry Department, Faculty of Science, Helwan University Cairo 11795 Egypt rasha_azzam@science.helwan.edu.eg; b Department of Clinical Pathology-Hematology, Ain Shams Medical Research Institute (MASRI), Faculty of Medicine, Ain Shams University Cairo 11566 Egypt; c Department of Genetics and Molecular Biology, Egypt Center for Research and Regenerative Medicine (ECRRM) Cairo 11599 Egypt

## Abstract

Cancer remains a worldwide healthcare undertaking, demanding continual innovation in anticancer drug development due to frequent drug resistance and adverse effects associated with existing therapies. The benzothiazole compounds, particularly 2-aminobenzothiazole derivatives, have attracted interest for their versatility in generating novel anticancer agents. This study explores the synthesis, and anticancer evaluation of new pyrimidine-based 2-aminobenzothiazole derivatives. A range of synthetic methods have been developed based on the reaction of 2-benzothaizolyl guanidine with various reagents such as α,β-unsaturated carbonyl, 2-cyano-three-(dimethylamino)-*N*-acrylamide, β-diketones, β-keto esters, and *S*,*S* ketene dithioacetals. Human tumour cell lines such as HepG2, HCT116, and MCF7 were used in *in vitro* cytotoxicity studies, and the results showed that several of the synthesized compounds were more potent than the standard drug, 5-fluorouracil, in terms of cell viability% with low IC_50_. Furthermore, the computed drug likeness and ADMET properties of the most potent synthesized compounds suggest their potential as promising candidates for further development, with favorable bioavailability and pharmacokinetic profiles.

## Abstract 2

2-Aminobenzothiazole derivatives, have attracted interest for their versatility in generating novel anticancer agents. This study explores the synthesis, and anticancer evaluation of new pyrimidine-based 2-aminobenzothiazole derivatives.

## Graphical abstract

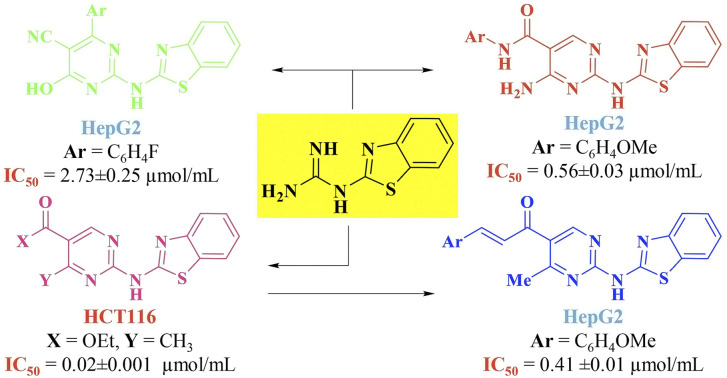

## 1. Introduction

**Fig. 1:**
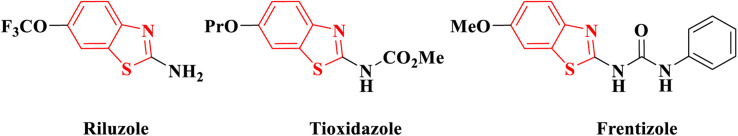
Commercial drugs having 2-aminobenzothiazole.

Cancer has emerged as a significant healthcare concern worldwide, with a rising number of cases over time. To address this issue, numerous anticancer drugs have been approved and are currently in clinical use. However, the challenges of drug resistance and adverse effects persist, creating a continual need for innovative, potent, and safe candidates for cancer therapy. In recent decades, researchers have explored and documented various heterocyclic ring-based derivatives in the literature. Notably, benzothiazole scaffold-based compounds have proven to be versatile rings for the development of novel and safe anticancer candidates. The scaffold of 2-aminobenzothiazole has undergone extensive exploration to create diverse analogues demonstrating remarkable biological activity against various targets. Notably, several therapeutic agents incorporating this framework have received clinical approval. One such example is riluzole, a vital drug based on 2-aminobenzothiazole, employed in treating amyotrophic lateral sclerosis, a severe neurodegenerative disorder^1^ (Fig. 1). Many studies have indicated its promising anti-tumor effects on various human solid cancer cell lines.^2^ Another significant compound is tioxidazole, an anthelmintic drug designed for the treatment of parasitic infections^3^ (Fig. 1). Additionally, frentizole, depicted in Fig. 1, serves as a non-toxic antiviral and immune suppressive agent utilized in clinical settings for conditions such as rheumatoid arthritis and systemic lupus erythematosus.^4^

**Fig. 2:**
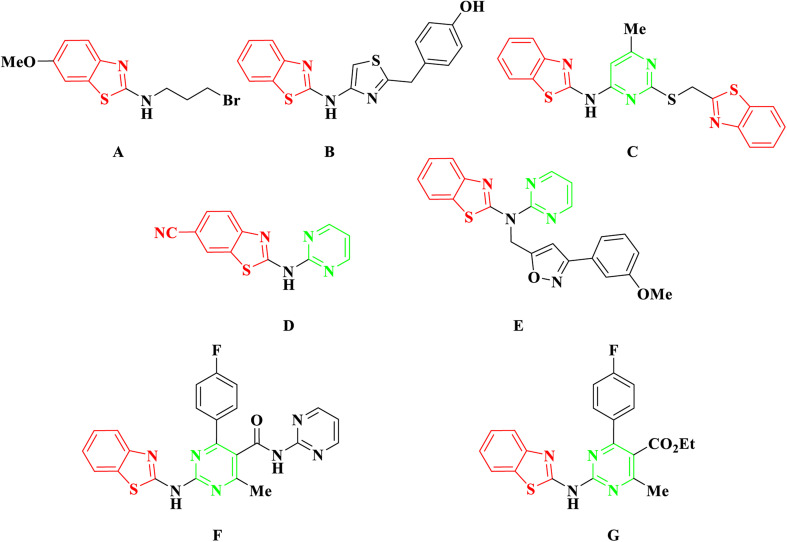
Examples of anticancer agents containing 2-aminobenzothiazole.

In recent developments, 2-aminobenzothiazole derivatives have emerged as novel antineoplastic agents, showcasing a diverse range of protein targets, including tyrosine kinases such as EGFR, CSF1R, VEGFR-2, MET, and FAK, serine/threonine kinases such as Aurora, CK, CDK, DYRK2, and RAF, mutant p53 protein, BCL-XL, PI3K kinase, HSP90, NSD1, HDAC, LSD1, DNA topoisomerases, FTO, mPGES-1, hCA IX/XII, SCD, and CXCR receptor.^5^ Concurrently, 2-aminobenzothiazole stands as a prominently featured scaffold in medicinal chemistry, prevalent in bioactive molecules, particularly those pertaining to cancer agents—exemplified by compounds A, B, and C,^6–8^Fig. 2. 2-Aminobenzothiazoles with a pyrimidine base, in particular, have demonstrated noteworthy anticancer activities against various cell lines and enzymes. For example, a series of cyano and amidinobenzothiazole-substituted anilins were synthesized and assessed for their antiproliferative effects on various tumor cell lines, such as Hep-2, MCF-7, HeLa, MiaPaCa-2, SW 620, and H 460. Notably, the pyrimidine-based carbonitrile benzothiazole derivative D exhibited potency against all cancer cell lines studied,^9^Fig. 2. Additionally, derivatives of 2-aminobenzothiazole, incorporating both isoxazole and pyrimidine rings, were synthesized and evaluated for their anticancer activity using the MTT assay across diverse cell lines, including A549, Colo205, MCF-7, and U937. Among them, compound E demonstrated notable anticancer efficacy against Colo205 and U937, exhibiting a potential IC_50_ value in comparison to the standard drug etoposide,^10^Fig. 2. Moreover, compounds F and G displayed notable efficacy against three leukemia cell lines and protein tyrosine kinase (PTK), demonstrating inhibitory concentrations of 0.131 μM and 0.161 μM, respectively.^11^

Various methods have been utilized for the synthesis of diverse derivatives of pyrimidine-based 2-aminobenzothiazole. One approach involves the nucleophilic substitution reaction of commercially available 2,4-dichloro-5-methylpyrimidine with 2-aminobenzothiazole at the C-4 position of the pyrimidine ring in the presence of sodium hydroxide (NaOH) at room temperature, yielding *N*-(2-chloro-5-methylpyrimidin-4-yl)benzo[*d*]thiazol-2-amine.^12^ Another method involves the reaction of 2-benzothaizolyl guanidine with various molecules. The synthesis of 2-benzothaizolyl guanidine involves treating 2-aminobenzothiazole with *S*-methyl isothiourea or reacting cyano guanidine with *o*-aminothiophenol in an acidic medium.^11,13^ The resultant 2-benzothaizolyl guanidine reacts with substituted benzaldehydes and ethyl acetoacetate, methyl acetoacetate, or ethyl cyanoacetate, following Biginelli's method with modifications, to yield pyrimidine-based 2-aminobenzothiazole derivatives.^11^ Additionally, 2-benzothaizolyl guanidine, when treated with methyl or ethyl acetoacetate in the presence of an excess of trimethylorthoacetate (TMOA) under nitrogen, results in another series of derivatives of pyrimidine-based 2-aminobenzothiazole, dependent on the involved 1,2-diketones.^14^ Further diversification is achieved by the reaction of 2-benzothaizolyl guanidine with different chalcones, diethyl malonate, or β-ketoester, either in aqueous or acidic medium.^13,15–18^ Trifluoromethyl-substituted *N*-(pyrimidin-2-yl)benzo[*d*]thiazol-2-amines are prepared through the cyclocondensation reaction of 2-benzothaizolyl guanidine with 4-alkoxy-4-alkyl(aryl/heteroaryl)-1,1,1-trifluoroalk-3-en-2-ones or 2,2,2-trifluoro-1-(2-methoxycyclohexen-1-en-1-yl)ethanone.^19^ Moreover, a series of pyrimidine-base 2-aminobenzothiazoles is obtained by reacting 2-benzothaizolyl guanidine with ethyl 2-butylacetoacetate, diethyl ethoxymethylenemalonate, ethyl ethoxymethylenecyanoacetate, and ethoxymethylenemalononitrile in a basic medium.^20^

In our previous investigations, we undertook the design and synthesis of a series of innovative benzothiazole derivatives in conjunction with pyrimidine,^21^ pyridine,^22,23^ purine analogues,^24^ or thiophene ring.^25^ These compounds were evaluated for their antimicrobial, antiviral, and/or anticancer activities.^26^ Recognizing the key role of 2-aminobenzothiazole as a promising anticancer agent, our exploration was encouraged to create new derivatives of 2-aminobenzothiazole, specifically in collaboration with the pyrimidine ring. This strategic modification aimed to further enhance the structural features and optimize the potency of the compounds. In this manuscript, we present our comprehensive investigation encompassing the synthesis, anticancer evaluation, and molecular docking studies of the newly designed pyrimidine-based 2-aminobenzothiazole derivatives.

## 2. Results and discussion

### 2.1. Chemistry

**Scheme 1:**
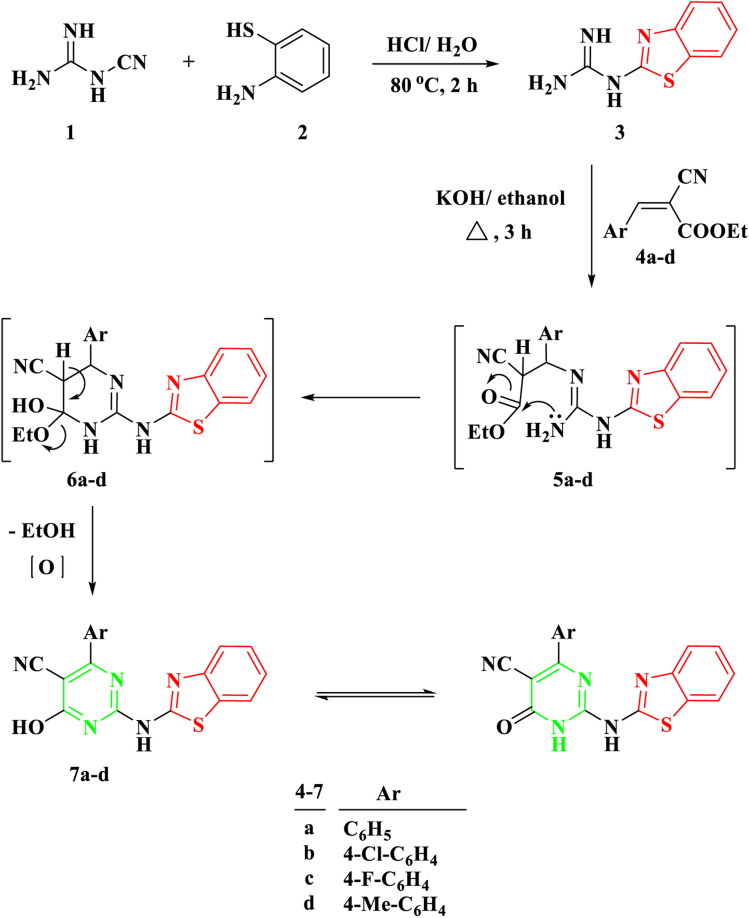
Synthesis of 2-(benzo[*d*]thiazol-2-ylamino)-6-oxo-4-aryl-1,6-dihydropyrimidine-5-carbonitrile 7a–d.

To effectuate the synthesis of our designated novel pyrimidine-base 2-aminobenzothiazole derivatives, the initiation of the synthetic pathway involved the utilization of 2-benzothiazolyl guanidine 3 as the foundational precursor. The synthesis of 2-benzothiazolyl guanidine was accomplished through a straightforward method. This method involved the condensation reaction between *o*-aminothiophenol 1 and cyanoguanidine 2, facilitated by an acidic medium and heating at 80 °C.^13^ This method was chosen based on the readily available starting materials and the overall efficiency and scalability of the process. Several α,β-unsaturated carbonyl compounds 4a–d, as illustrated in Scheme 1, underwent a reaction with 2-benzothiazolyl guanidine 3 to produce 2-aminobenzothiazoyl pyrimidine 7a–d, featuring a cyano group at position 5. The identification of the synthesized compounds 7a–d was established through spectral analyses, including FT-IR, ^1^H NMR, and ^13^C NMR. The IR spectra distinctly indicated the presence of a CN group, evident from a singular band at approximately *ν* 2209–2210 cm^−1^. In the ^1^H NMR spectra of 5a–d, a broad signal within the range of *δ* 11.19–12.69 ppm affirmed the NH proton's existence. Furthermore, the ^1^H NMR spectra of compounds 5a–d revealed four distinct signals corresponding to the four protons of the benzene ring in the benzothiazole ring. These signals included two triplets within the range of *δ* 7.13–7.39 ppm and two doublets within the range of *δ* 7.65–8.01 ppm, with each signal representing one proton. Conversely, the ^13^C NMR of 7a exhibited a signal at *δ* 119 ppm for the CN group and another at *δ* 169 ppm for the CO group.

The proposed mechanism for the generation of compounds 7a–d initiates with a Michael addition involving the amino group of 2-benzothiazolyl guanidine 3 and the double bond of the α,β-unsaturated carbonyl compounds 4a–d, intermediate 5. This is followed by intramolecular cyclization through the addition of the NH proton to the carbonyl group of COOEt, intermediate 6, and the elimination of EtOH.

**Scheme 2:**
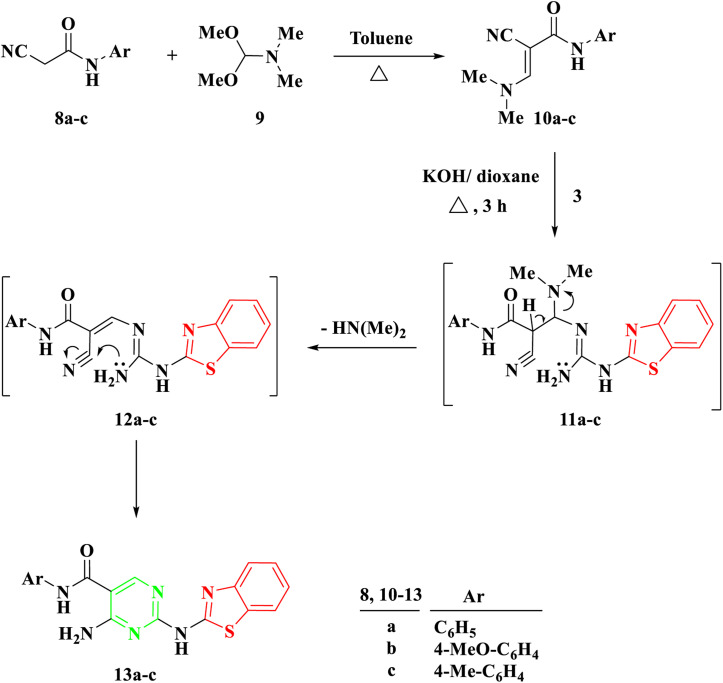
Synthesis of 2-(benzo[*d*]thiazol-2-ylamino)-*N*-arylpyrimidin-5-carboxamide 13a–c.

Another procedure produced 5-carboxamide-2-aminobenzothiazoyl pyrimidine 13a–c by reacting 2-benzothiazolyl guanidine 3 with 2-cyano-3-(dimethylamino)-*N*-acrylamide 10a–c, which were prepared by the reaction of DMF-DMA 9 with derivatives of aryl carboxamide derivatives 8a–c.^27^ The process created intermediate 11a–c by nucleophilically adding the amino group to the double bond through Michael addition. The removal of a (CH_3_)_2_NH molecule, the formation of intermediate 12, and finally cyclization were the next processes, which resulted in the target molecules 13a–c shown in Scheme 2. Based on IR and spectral data, the structure of compounds 13a–c was interpreted. For example, a singlet signal at *δ* 3.75 ppm in the ^1^H NMR spectrum of 13b suggested the existence of a methoxy group. Additionally, two doublet signals at *δ* 6.93 and 7.59 represented two protons for each signal, corresponding to the aromatic ring of 4-methoxybenzene. Finally, a singlet signal at *δ* 8.78 ppm was assigned to (pyrimidine-H), and two singlet signals at *δ* 10.04 and 11.73 ppm were assigned to the NH and NH_2_ groups, respectively.

**Scheme 3:**
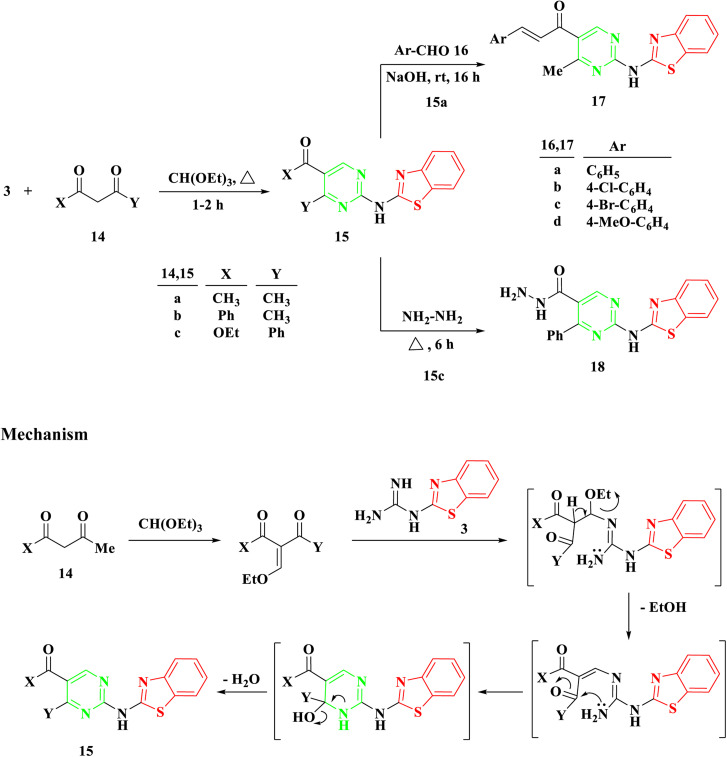
Synthesis of 2-(benzo[*d*]thiazol-2-ylamino)-4-phenylpyrimidine 15a–c, 1-(2-(benzo[*d*]thiazol-2-ylamino)-4-methylpyrimidin-5-yl)-3-arylprop-2-en-1-one 17a–d, and 2-(benzo[*d*]thiazol-2-ylamino)-4-phenylpyrimidine-5-carbohydrazide 18.

Benzothiazolyl guanidine 3 underwent additional treatment with β-diketones, including acetyl acetone 14a, benzoylacetone 14b, and β-keto esters like ethylbenzoyl acetone 14c, in the presence of an excess of triethyl orthoformate under reflux for 1–2 hours. This resulted in the formation of 2-aminobenzothiazol acylpyrimidine 15a and b and 2-aminobenzothiazolyl ethoxycarbonylpyrimidine 15c, Scheme 3. The mechanism appears to involve the initial formation of ethoxy compounds derived from both β-diketones and β-keto esters, followed by the generation of a linear intermediate through ethanol removal and subsequent intramolecular condensation.

Utilizing symmetrical and unsymmetrical diketones, namely 14a and 14b, respectively, yielded a single cyclization product based on NMR spectra. Additionally, reactions involving benzothiazolyl guanidine 3 and triethyl orthoformate with β-keto ester 14c, potentially resulting in two intermediates, produced a singular cyclization product, 2-aminobenzothiazolyl ethoxycarbonylpyrimidine 15c, without the formation of hydroxyacylpyrimidine. The existence of the ester group in compound 15c was confirmed by observing a triplet and quartet of the ethoxy group at chemical shifts of *δ* 1.07 and 4.14 ppm, respectively, in its ^1^H NMR spectra.

The Aldol condensation reaction was carried out on compound 15a with aromatic aldehyde derivatives to produce the corresponding chalcones, which feature an α,β-unsaturated carbonyl system. Chalcones, a subgroup of flavonoids, were synthesized in this study by reacting 2-aminobenzothiazolyl acylpyrimidine 15a with substituted aromatic aldehydes 16a–d in a basic medium, using ethanol as the solvent. This process led to the formation of 2-aminobenzothiazolyl pyrimidine-linked chalcones 17a–d, Scheme 3. The structure of the newly synthesized compounds was determined through IR and NMR spectroscopy. For instance, the IR spectrum of 17a revealed an absorption band at 1663 cm^−1^, indicating the presence of a C=O group (conjugated ketone). In the ^1^H NMR spectrum of 17b, two doublet signals at *δ* 7.58 and 7.82 ppm, with a coupling constant of 15.5 Hz, provided evidence of the E configuration of the produced chalcones.

Following a 6 hours reflux in the presence of excess hydrazine hydrate, 2-aminobenzothiazolyl ethoxycarbonylpyrimidine 15c was entirely utilized to generate the corresponding hydrazide 18. Upon cooling, a white solid with a melting point of 294–295 °C was isolated. The IR spectra exhibited a band at 1628 cm^−1^, indicative of the amide group's carbonyl (CO) functionality. The ^1^H NMR analysis confirmed the absence of the ethoxycarbonyl group in the initial compound 15c, and revealed the presence of NH_2_ at *δ* 4.51 ppm and NH at *δ* 9.66 ppm in the produced hydrazide group.

**Scheme 4:**
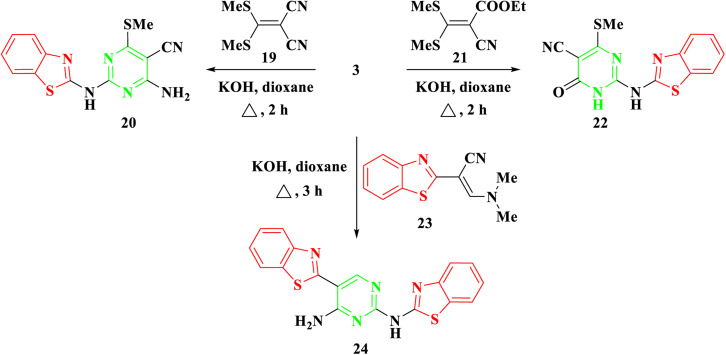
Synthesis of 2-(benzo[*d*]thiazol-2-ylamino)-4/6-(methylthio)pyrimidine 20, 22, & 24.

Furthermore, our investigation was extended to encompass the reaction of benzothiazolyl guanidine 3 with *S*,*S* ketene dithioacetals, such as 2-(bis-(methylthio)methylene)malononitrile 19 and ethyl 2-cyano-3,3-bis(methylthio)acrylate 21, as depicted in Scheme 4. The reaction was conducted using KOH in dioxane, yielding the respective 2-aminobenzothiazol-4-methylthio pyrimidine, 20, and 22. The suggested synthetic route for the target compounds entails the incorporation of the amino group of 3 into the ylidene bond in 19 and 21. Subsequently, this is followed by either eliminating an ethanol molecule when utilizing compound 21 or adding to the cyano group when utilizing compound 19. Finally, the cyclization occurs *via* the addition of the NH group to the cyano group. Elemental analysis and spectral data confirmed the proposed structures of compounds 20 and 22. The IR spectra clearly indicated the presence of NH and CN groups in both 20 and 22, as evidenced by absorption bands at 3378–3379 and 2198–2208 cm^−1^, respectively. The ^1^H NMR of 20 and 22 revealed a singlet signal at *δ* 2.69–2.72 ppm, confirming the presence of SCH_3_ protons. In the case of compound 20, a broad signal at *δ* 7.70 ppm affirmed the existence of NH_2_ groups. Additionally, the ^13^C NMR spectra of compounds 20 and 22 displayed signals at *δ* 40.4–40.5 ppm for the SCH_3_ group and signals at *δ* 115.6–118.4 ppm for the CN group.

Moreover, in the presence of potassium hydroxide, 2-benzothiazolyl guanidine 3 was reacted with 2-benzothiazolyl enaminoacrylonitrile 23.^28^ As shown in Scheme 4, this reaction produced *N*^2^,5-bisbenzothiazolyl pyrimidine 24. Compound 24's structure was determined by thoroughly analyzing its IR and NMR spectra. The IR spectra showed characteristic absorption bands for the NH_2_ and NH groups, which were situated at around 3463 and 3266 cm^−1^, respectively. Four protons of the two benzothiazole rings were attributed to four doublet signals in the ^1^H NMR spectra at *δ* 7.69, 7.90, 8.06, and 8.13 ppm. The CH proton was also allocated a separate singlet signal at *δ* 8.78 ppm. The reaction mechanism involved the Michael addition of the amino group of benzothiazolyl guanidine 3 to the double bond of the enamine, resulting in the elimination of NH(CH_3_)_2_. Subsequently, intramolecular cyclization occurred through the addition of the amino group to the cyano group, ultimately yielding the pyrimidine derivative 24.

### 2.2. *In vitro* cytotoxic activity

The cytotoxic impact of novel pyrimidine-based 2-aminobenzothiazole derivatives 7a–d, 13a–c, 15a–c, 17a–d, 18, 20, 22, 24 was assessed utilizing the standard 3-(4,5-dimethylthiazol-2-yl)-2,5-diphenyltetrazolium bromide (MTT) bioassay. In the initial phase, the synthesized compounds underwent screening for their anticancer activities against three human tumor cell lines, HepG2, HCT116, and MCF7, at a singular concentration 100 μmol mL^−1^. Cell viability percentages were utilized as the metric for assessing outcomes, and comparisons were made with 5-fluorouracil, chosen as the standard positive control drug. 5-Fluorouracil is a chemotherapeutic agent employed in the treatment of diverse malignancies including gastric adenocarcinoma, pancreatic adenocarcinoma, breast carcinoma, and colorectal adenocarcinoma.^29^ The results are detailed in Tables 1 and S1–S6 (ESI)†

**Table 1:** Viability% of synthesized compounds against HepG2, HCT116 and MCF7 cell lines

Comp.	Cell viability (%)		
	HepG2	HCT116	MCF7
7a	85.64	75.12	90.22
7b	72.70	70.62	99.38
7c	61.29	87.26	102.39
7d	95.53	91.33	95.58
13a	72.35	73.66	74.45
13b	68.18	88.68	90.71
13c	83.88	67.11	87.78
15a	81.16	86.64	101.19
15b	94.96	87.75	97.54
15c	72.69	65.68	98.64
17a	82.35	85.90	88.48
17b	76.02	89.53	102.2
17c	80.45	85.90	100.03
17d	61.04	87.15	99.66
18	66.85	91.21	95.11
20	95.01	84.71	84.17
22	81.68	88.29	99.89
24	78.41	87.84	69.98
**5-Flu**	**64.41**	**55.96**	**62.76**

In light of the obtained results, four compounds 7c, 13b, 17d, and 18 demonstrated strong efficacy against the HepG2 cell line, exhibiting cell viability percentages of 61.29, 68.18, 61.04, and 66.85, respectively, in comparison to the standard drug with a cell viability percentage of 64.41. Another compounds showed moderate activities against HepG2 cell line such as 7b, 13a, 15c, and 17b with cell viability percent of 72.70, 72.35, 72.69 and 76.02. Additionally, three compounds 7b, 13c, and 15c exhibited slightly strong efficacy against the HCT116 cell line, displaying cell viability percentages of 70.62, 67.11, and 65.68, respectively, in contrast to the standard drug with a cell viability percentage of 55.96. Furthermore, a singular compound 24 demonstrated efficacy against the MCF7 cell line, with a cell viability percentage of 69.98, compared to the standard drug with a cell viability percentage of 62.76.

**Fig. 3:**
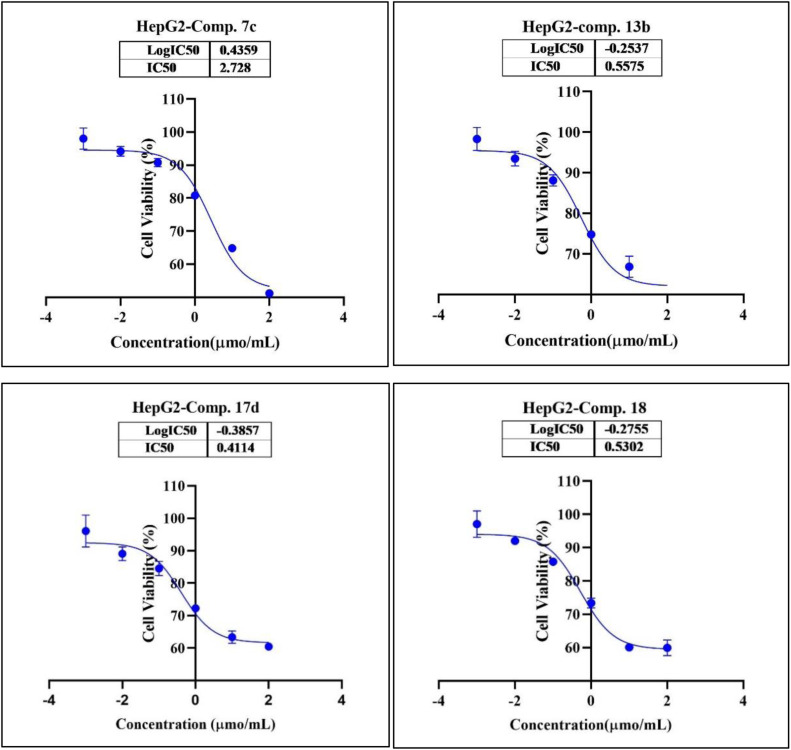
Nonlinear regression curve illustrating the log dose of pyrimidine derivatives 7c, 13b, 17d and 18*versus* the normalized response in HepG2 cells after treatment with serial concentrations in DMEM for 72 hours.

**Fig. 4:**
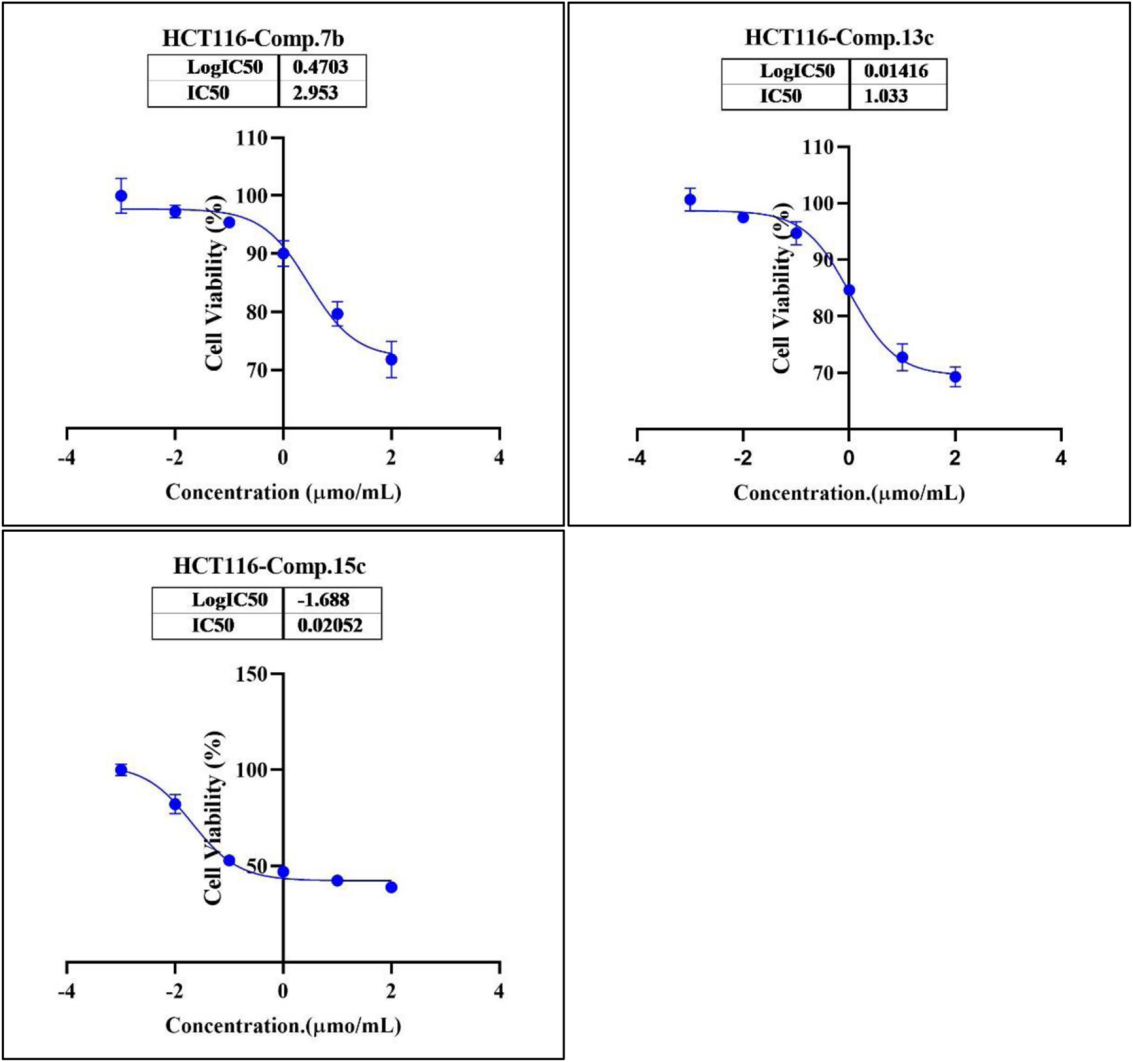
Nonlinear regression curve illustrating the log dose of pyrimidine derivatives 7b, 13c and 15c*versus* the normalized response in HCT116 cells after treatment with serial concentrations in DMEM for 72 hours.

**Fig. 5:**
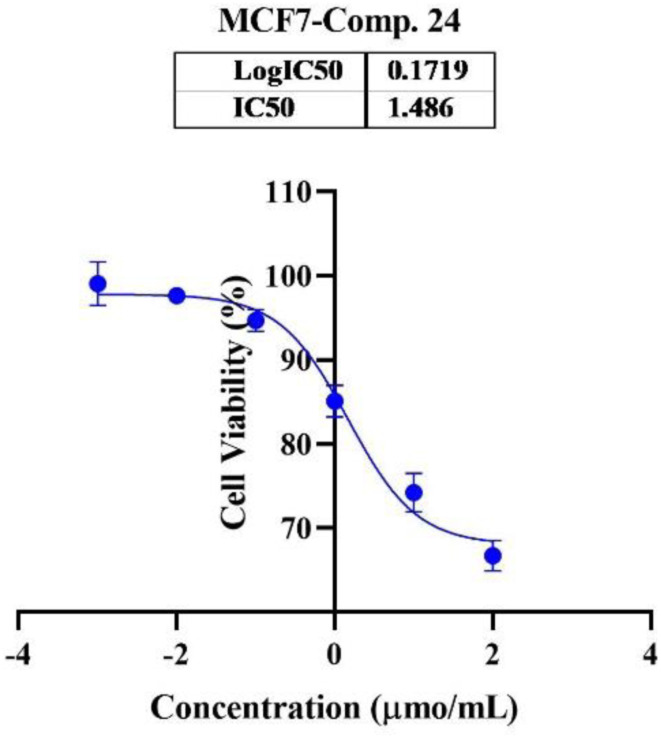
Nonlinear regression curve illustrating the log dose of pyrimidine derivative 24*versus* the normalized response in MCF7 cells after treatment with serial concentrations in DMEM for 72 hours.

In the second phase and based on the screening results, the IC_50_, which represents the compound concentrations required to produce a 50% inhibition of cell growth after 72 h of incubation, was measured for the most potent compounds. Specifically, compounds 7c, 13b, 17d, and 18 were evaluated for the HepG2 cell line. For the HCT116 cell line, compounds 7b, 13c, and 15c were assessed, and compound 24 was tested for the MCF7 cell line. The IC_50_ values were determined through analysis of the concentration–inhibition response curve, Fig. 3–5. Subsequent comparison ensued with the corresponding values attributed to the reference drug, 5-fluorouracil. To determine the IC_50_ values, a range of five different concentrations (100, 10, 1, 0.1, 0.01 μmol mL^−1^) for the tested compounds was applied. The resultant IC_50_ values of tested compounds, in conjunction with those pertaining to the standard drug, are outlined in Tables 2–4. From the IC_50_ results, it was indicated that compound 17d was the most potent pyrimidine-based 2-aminobenzothiazole derivative overall the tested compounds against HepG2 with IC_50_ 0.41 ± 0.01 μmol mL^−1^. The second most potent compound against HepG2 is compound 18 with IC_50_ 0.53 ± 0.05 μmol mL^−1^ followed by compound 13b with IC_50_ of 0.56 ± 0.03 μmol mL^−1^. Notably, three of the newly synthesized compounds, 17d, 18 and 13b, demonstrated higher potency, based on the resulting IC_50_ data, compared to 5-fluorouracil, which has an IC_50_ of 1.03 μmol mL^−1^.^30^ Surprisingly, compound 15c displayed an IC_50_ of 0.02 ± 0.001 μmol mL^−1^, indicating superior efficacy against HCT116 compared to 5-fluorouracil, which exhibited an IC_50_ of 9 ± 1.7 μmol mL^−1^.^31^ Compound 15c not only demonstrated heightened potency relative to 5-fluorouracil but also exhibited notable efficacy alongside compounds 7b and 13c, which displayed IC_50_ values of 2.95 ± 0.26 and 1.033 ± 0.06, respectively. Additionally, compound 24 exhibited IC_50_ value, 1.485 ± 0.15 μmol mL^−1^, lower than the IC_50_ value of 5-flouracel, 7.12 μmol mL^−1^, against MCF7.^32^ These results suggest that the investigated compounds exhibit potential as robust anticancer agents.

**Table 2:** Determination of IC_50_ of compounds 7c, 13b, 17d and 18 on HepG2 cells

Comp.	Conc. μmol mL^−1^					IC_50_ μmol mL^−1^
	0.01	0.1	1	10	100	
7c (viability%)	95.42%	91.76%	81.74%	66.36%	51.32%	2.73 ± 0.25
13b (viability%)	87.97%	80.39%	75.02%	65.36%	58.06%	0.56 ± 0.03
17d (viability%)	87.66%	81.14%	70.22%	62.38%	58.87%	0.41 ± 0.01
18 (viability%)	91.57%	85.88%	73.98%	60.31%	72.06%	0.53 ± 0.05

**Table 3:** Determination of IC_50_ of compounds 7b, 13c and 15c on HCT116 cells

Comp.	Conc. μmol mL^−1^					IC_50_ μmol mL^−1^
	0.01	0.1	1	10	100	
7b (viability%)	96.70%	95.43%	88.77%	80.17%	71.89%	2.95 ± 0.26
13c (viability%)	97.85%	95.79%	84.14%	79.11%	69.42%	1.033 ± 0.06
15c (viability%)	85.04%	53.51%	47.71%	42.95%	39.18%	0.02 ± 0.001

**Table 4:** Determination of IC_50_ of compound 24 on MCF7 cells

Comp.	Conc. μmol mL^−1^					IC_50_ μmol mL^−1^
	0.01	0.1	1	10	100	
24 (viability%)	97.67%	94.28%	84.67%	74.00%	66.50%	1.485 ± 0.15

The findings from this study reveal that the inclusion of halogen groups, specifically F and Cl, on the aryl group bonded with pyrimidine-based 2-aminobenzothiazole 7a–d resulted in an increase of compound activity. Additionally, the incorporation of CO_2_Et, as observed in compound 15c, enhanced the potency of the compound in comparison to its analogs, 15a and 15b, containing COCH_3_ and COPh, respectively. In the context of pyrimidine-based 2-aminobenzothiazole 17a–d, the presence of a methoxy group was found to amplify the compound's potency more significantly than those possessing halogen substituents. Notably, compounds featuring SCH_3_ exhibited the lowest activity levels across the three tested cell lines.

### 2.3. Drug likeness, and physicochemical–pharmacokinetic/ADMET properties

To assess the potential of the synthesized compounds as drug candidates, various parameters including drug likeness, adherence to specific rules, and ADMET (Absorption, Distribution, Metabolism, Excretion, and Toxicity) properties were computed using Molsoft software and the SwissADME program.^33^ Poor oral bioavailability in drug discovery is often associated with characteristics such as more than five hydrogen bond donors, ten hydrogen bond acceptors, a molecular weight exceeding 500 g mol^−1^, and a calculated log *P* greater than 5.

Analysis of the results in Table 5 indicates that all potent compounds exhibited only one or no violation in these criteria. Specifically, none of the compounds surpassed the normal range for the number of hydrogen bond donors, number of hydrogen bond acceptors, and log *P*. Moreover, all compounds demonstrated a drug-likeness score within the range of 0.05 to 0.84. Further examination revealed that the molecular weight and topological polar surface area (TPSA) of compounds 7b, 7c, 13c, 15c, 17d, and 18 did not exceed the standard limits of 500 g mol^−1^ and TPSA of 140 Å^2^, except for compounds 13b and 24, which exhibited slightly higher TPSA values of 143.29 and 146.09 Å^2^, respectively.

**Table 5:** Drug likeness predictions and physicochemical–pharmacokinetic/ADMET properties of the most active compounds

No	Mwt	Number of HBA^a^	Number of HBD^b^	log *P*_o/w_ (iLOGP)^c^	TPSA^d^	Lipinski, Ghose, Veber, Egan, and Muegge violations	Drug-likeness model score
7b	379.82	5	2	2.84	122.96	1, 1, 1, 1, 0	0.38
7c	363.37	6	2	2.61	122.96	1, 1, 1, 1, 1	0.27
13b	392.43	5	3	2.90	143.29	1, 1, 0, 0, 1	0.84
13c	376.43	4	3	2.79	134.06	1, 1, 1, 0, 1	0.73
15c	376.43	5	1	3.31	105.24	1, 1, 1, 1, 1	0.50
17d	402.47	5	1	3.44	105.24	1, 1, 1, 1, 0	0.64
18	362.41	5	3	1.15	134.06	1, 1, 1, 0, 1	0.51
24	376.46	4	2	2.99	146.09	1, 1, 0, 0, 1	0.05

a Number of hydrogen bond acceptors.

b Number of hydrogen bond donors.

c Lipophilicity.

d Topological polar surface area.

Furthermore, the investigation into blood–brain barrier (BBB) permeability, gastrointestinal (GI) absorption, and bioavailability of the synthesized compounds was conducted using the SwissADME program, Table 6. The results presented in Table 6 indicate that all potent synthesized compounds exhibit no blood–brain barrier permeability, suggesting their inability to traverse the BBB. Conversely, compounds such as 7b, 7c, 13b, 13c, and 24 demonstrate low GI absorption, revealing of favorable absorption in the human intestine. In contrast, compounds 15c, 17d, and 18 exhibit high GI absorption. Furthermore, all potent compounds boast a bioavailability score of 0.55, implying favorable pharmacokinetic properties.

**Table 6:** Predicted ADMET properties of the tested compounds

No	GI absorption	BBB permeant	Bioavailability score	CYP-substrate/inhibitor				
				3A4	1A2	2C19	2C9	2D6
7b	Low	No	0.55	Yes	Yes	Yes	Yes	Yes
7c	Low	No	0.55	Yes	Yes	No	Yes	Yes
13b	Low	No	0.55	Yes	Yes	Yes	Yes	Yes
13c	Low	No	0.55	Yes	Yes	Yes	Yes	Yes
15c	High	No	0.55	Yes	Yes	Yes	Yes	Yes
17d	High	No	0.55	Yes	Yes	Yes	Yes	Yes
18	High	No	0.55	Yes	Yes	Yes	No	No
24	Low	No	0.55	Yes	Yes	No	Yes	Yes

### 2.4. Docking study

Protein tyrosine kinases (PTKs) play a crucial role in regulating the proliferation, differentiation, and signaling processes within immune system cells. They are broadly categorized into transmembrane receptor-linked kinases and cytoplasmic kinases.^34^ Abnormal signaling from both receptor tyrosine kinases and intracellular tyrosine kinases can contribute to various diseases, particularly cancer, including non-small cell lung cancer, chronic myeloid leukemia, and gastrointestinal stromal tumors.^35^ Due to its significant involvement in cancer etiology, the PTK receptor has been a subject of considerable attention for a considerable period. Aminobenzazoles, such as aminobenzothiazole, aminobenzoxazoles, and benzimidazoles, when paired with pyrimidines, have emerged as potential inhibitors of PTK.^11^ Given the structural similarity between our synthesized compounds and previously proven potent compounds against protein tyrosine kinases,^11^ we conducted molecular docking studies on the PTK receptor (PDB ID: 2GQG) to elucidate the interactions of the most effective compounds with the PTK binding site.

**Fig. 6:**
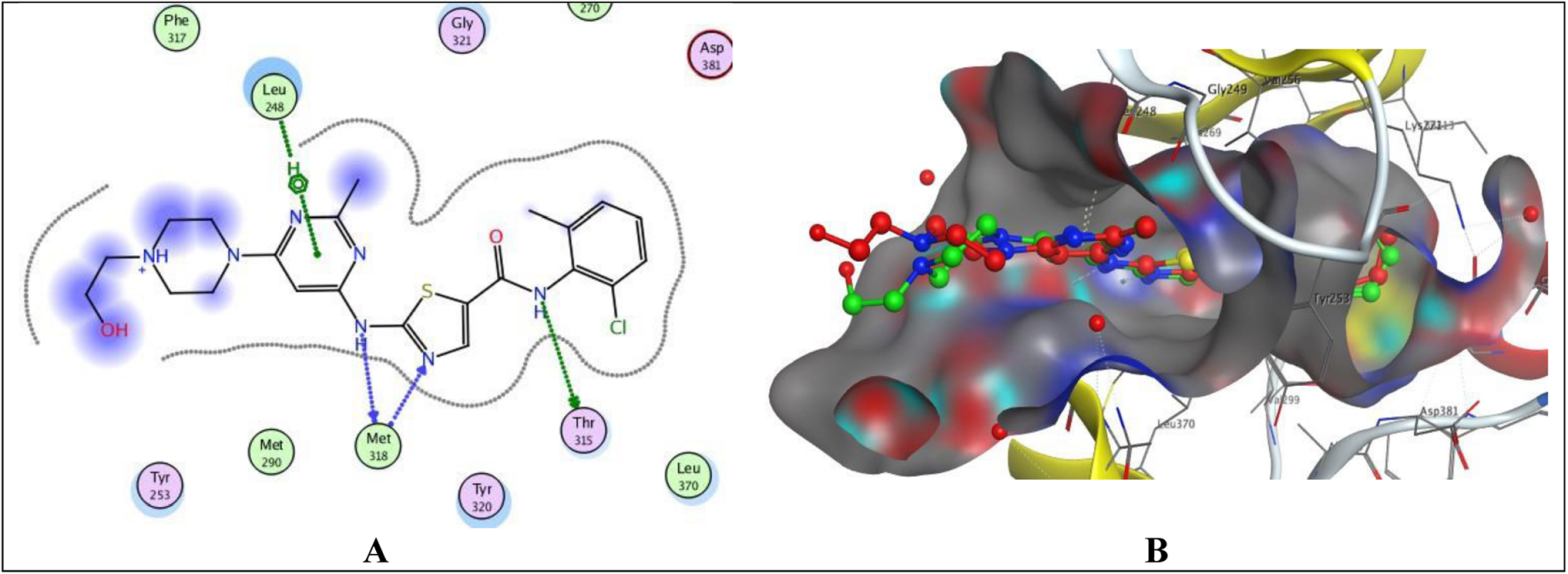
Docking poses of 1N1 ligand inside PTK active site. (A) 2D interaction of 1N1 ligand with active site. (B) 3D docking of 1N1 ligand for validation.

The validation of the docking study involved placing the cocrystallized ligand (1N1) inside the active site after extraction from the respective receptor, as illustrated in Fig. 6. The docking of the cocrystallized ligand 1N1 yielded a root mean square deviation value of 1.1559 and binding energy −9.2907 kcal mol^−1^, Table 7. The results indicated that 1N1 formed one H-bond acceptor with Met318, two H-bond donors with Met318 and Thr315, and one arene–H interaction with Leu248. Fig. 6A and B depicts the various types of interactions between the ligand 1N1 and the PTK active site.

**Table 7:** Molecular docking free binding energy and bond interactions estimate to PTK receptor

Comp.	Binding energy (kcal mol^−1^)	Arene–H interactions	H-bond acceptor	H-bond donor
7b	−6.8897	Val256	—	Met318 (2.83 Å)
				Glu316 (2.21 Å)
7c	−6.5024	—	—	Met318 (2.88 Å)
				Gly249 (3.34 Å)
13b	−6.9625	Leu248	—	Met318 (2.06 Å)
				Met318 (Å)
				Thr319 (Å)
13c	−6.9727	Leu248	—	Met318 (2.10 Å)
15c	−7.7288	Val256	—	Glu316 (3.02 Å)
17d	−7.3209	—	—	Met318 (3.14 Å)
18	−7.6805	—	Met318 (3.61 Å)	
24	−7.5790	—	—	Met318 (2.88 Å)
				Met318 (3.35 Å)
				Met318 (3.49 Å)
1N1	−9.2907	Leu248	Met318 (2.86 Å)	Met318 (2.86 Å)
				Thr315 (2.84 Å)

**Fig. 7:**
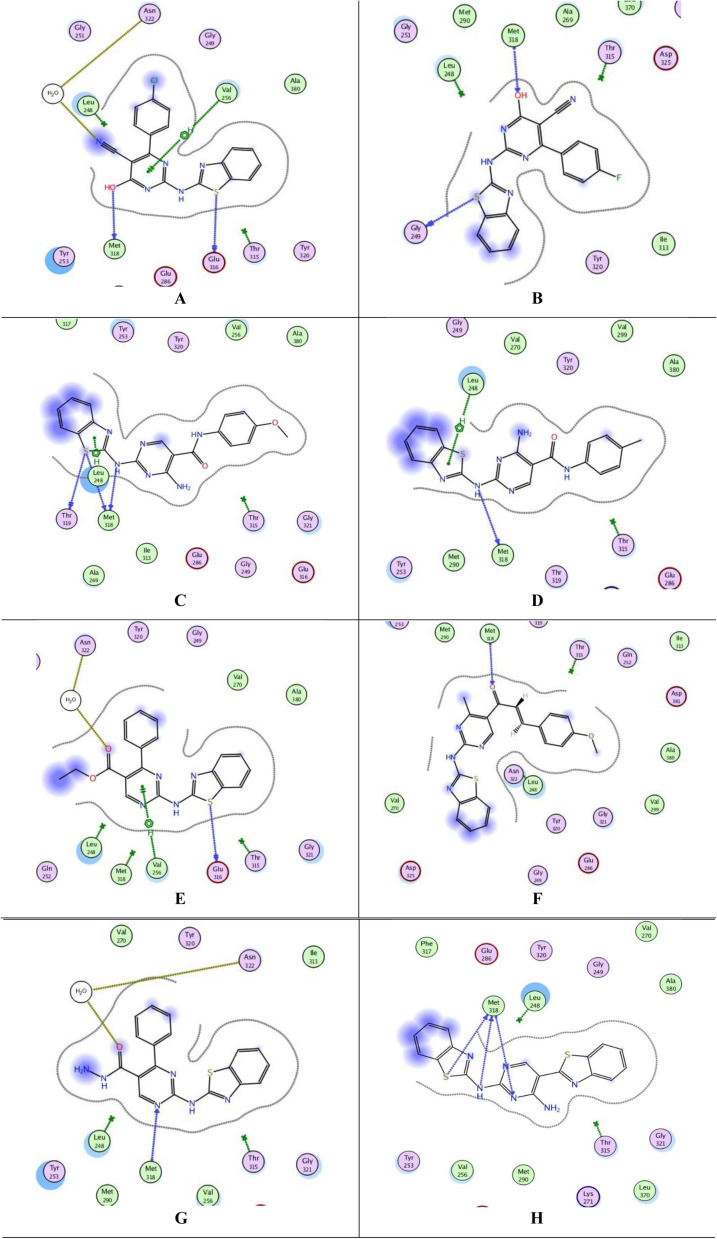
Docking poses of compounds 7b, 7c, 13b, 13c, 15c, 17d, 18 and 24 inside PTK active site. (A) 2D interaction of 7b. (B) 2D interaction of 7c. (C) 2D interaction of 13b. (D) 2D interaction of 13c. (E) 2D interaction of 15c. (F) 2D interaction of 17d. (G) 2D interaction of 18. (H) 2D interaction of 24.

The top-ranked poses of the most active compounds, 7b, 7c, 13b, 13c, 15c, 17d, 18 and 24, within the active site of PTK are summarized in Fig. 7A–H. Notably, the docking analysis revealed that all compounds fit inside the active site. It was observed that all compounds formed a hydrogen donor bond with Met318, except for 15c. Among these compounds, 15c, 17d, 18, and 24 exhibited binding energies closer to the cocrystallized ligand 1N1, with values of −7.7288, −7.3209, −7.6805, and −7.5790 kcal mol^−1^, respectively, Table 7. Despite compound 13c having a binding energy of −6.9625 kcal mol^−1^, it demonstrated four interactions with the active site, including one arene–H interaction with Leu248 and three H-bond donors with Met318 and Thr319. Observations revealed that all compounds, particularly 13c, 15c, 17d, 18, and 24, demonstrated promising interactions with the active site of PTK. However, it is important to note that we were unable to conduct an *in vitro* study due to the unavailability of the required kit.

## 3. Conclusion

In conclusion, our investigation has led to synthesis, and evaluation of new derivatives of pyrimidine-based 2-aminobenzothiazole as potential anticancer agents. The study has successfully elucidated and developed various synthetic routes, leading to a high yield of pyrimidine-based 2-aminobenzothiazole compounds. The confirmation of the desired compounds was achieved through comprehensive analytical and spectral analyses. The results of *in vitro* cytotoxicity studies revealed that several synthesized compounds displayed potent activities against human tumor cell lines, including HepG2, HCT116, and MCF7. Compounds 17d, 18, and 13b demonstrated notable efficacy, with IC_50_ values lower than that of the reference drug, 5-fluorouracil, in the case of HepG2. Moreover, compound 15c exhibited superior potency compared to 5-fluorouracil against HCT116. The physicochemical and pharmacokinetic properties of the synthesized compounds were assessed, and the majority adhered to drug-likeness criteria, indicating their potential as drug candidates. Furthermore, based on the docking study and a comparative analysis with previously similar compounds, the identified potent compounds exhibit promising potential as inhibitors of PTK. The overall findings suggest that these newly synthesized derivatives of pyrimidine-based 2-aminobenzothiazole hold promise as novel anticancer agents, warranting further exploration and optimization for potential clinical applications.

## 4. Experimental

### 4.1. Chemistry

An SMP3 melting point equipment was used to determine melting points. The ^1^H NMR spectra (400 MHz) were obtained at Ain Shams University in Cairo, Egypt, using a Bruker Advance (III)-400 MHz Spectrometer. The ^13^C NMR spectra (125 MHz) were collected using a Bruker Advance (III)-600 MHz Spectrometer at Helwan University's Central Laboratory, Hub of Creativity and Scientific Research. Some spectra were set on the APT system which produces positive methene (CH) and methyl (CH_3_) signals (odd) and negative (even) signals along with solvent signals. The solvent used for NMR experiments was DMSO-*d*_6_, with Si(CH_3_)_4_ serving as the internal standard. Chemical shifts are recorded in parts per million (ppm), and all coupling constants (*J* values) are given in Hertz. The following acronyms are used in NMR analysis: “s” for singlet, “d” for doublet, and “m” for multiplet. The progress of reactions and the analysis of product mixtures were monitored on a regular basis using thin layer chromatography (TLC) on silica gel pre-coated F254 plates Merck, and using UV lamp.

Because certain compounds exhibited low solubility in DMSO-*d*_6_, their ^13^C NMR spectra were not recorded.

#### 4.1.1 General procedure for the synthesis of 2-(benzo[*d*]thiazol-2-ylamino)-6-oxo-4-aryl-1,6-dihydropyrimidine-5-carbonitrile (7a–d)

A solution comprising benzothiazole guanidine 3 (1 mmol) and arylidene ethyl cyanoacetate 4a–d (1.2 mmol) in ethanol absolute (10 mL) with potassium hydroxide (1.3 mmol) was refluxed for 6–8 hours, as determined by TLC. After cooling, the reaction mixture was added to ice acidified with HCl. The resulting solid product was filtered, washed with ethyl acetate, and ethanol, yielding the respective products.

##### 4.1.1.1 2-(Benzo[*d*]thiazol-2-ylamino)-6-oxo-4-phenyl-1,6-dihydropyrimidine-5-carbonitrile (7a)

White solid (yield 66.6%), m.p. 347–348 °C; IR (KBr, *ν* cm^−1^): 3058 (Ar-CH), 2209 (CN), 1624 (C=O); ^1^H NMR (400 MHz, DMSO-*d*_6_): *δ* 7.13 (t, *J* = 7.6 Hz, 1H, benzothiazole-CH), 7.30 (t, *J* = 7.6 Hz, 1H, benzothiazole-CH), 7.48–7.60 (m, 3H, Ar-CH), 7.79 (d, *J* = 7.6 Hz, 1H, benzothiazole-CH), 7.87 (d, *J* = 7.6 Hz, 1H, benzothiazole-CH), 7.95–7.96 (m, 2H, Ar-CH), 11.19 (br, 1H, NH); ^13^C NMR (125 MHz, DMSO-*d*_6_): *δ* 119.7 (CN), 120.3, 121.3, 122.1, 125.7, 128.6, 128.9, 129.4, 129.7, 130.5, 131.6, 133.5, 138.2, 147.4, 150.3, 157.9, 169.1 (Ar,C); anal. calcd for C_18_H_11_N_5_OS (345.07): calc C% 62.60; H%3.21; N% 20.28; S% 9.28; found C% 62.64; H%3.19; N% 20.30; S% 9.31.

##### 4.1.1.2 2-(Benzo[*d*]thiazol-2-ylamino)-4-(4-chlorophenyl)-6-oxo-1,6-dihydropyrimidine-5-carbonitrile (7b)

White solid (yield 70%), m.p. over 350 °C; IR (KBr, *ν* cm^−1^): 3020 (Ar-CH), 2210 (CN), 1694 (C=O); ^1^H NMR (400 MHz, DMSO-*d*_6_): *δ* 7.22 (t, *J* = 8.4 Hz, 1H, benzothiazole-CH), 7.39 (t, *J* = 8.4 Hz, 1H, benzothiazole-CH), 7.62 (d, *J* = 8.4 Hz, 1H, benzothiazole-CH), 7.66 (d, *J* = 8.0 Hz, 2H, Ar-CH), 7.84 (d, *J* = 8.4 Hz, 1H, benzothiazole-CH), 8.01 (d, *J* = 8.8 Hz, 2H, Ar-CH), 12.61 (br, 1H, NH); anal. calcd for C_18_H_10_ClN_5_OS (379.82): calc C% 56.92; H% 2.65; N% 18.44; S% 8.44; found C% 56.95; H% 2.66; N% 18.41; S% 8.48.

##### 4.1.1.3 2-(Benzo[*d*]thiazol-2-ylamino)-4-(4-fluorophenyl)-6-oxo-1,6-dihydropyrimidine-5-carbonitrile (7c)

White solid (yield 68%), m.p. over 350 °C; IR (KBr, *ν* cm^−1^): 3031 (NH), 3021 (Ar-CH), 2210 (CN), 1694 (C=O); ^1^H NMR (400 MHz, DMSO-*d*_6_): *δ* 7.21 (d, *J* = 8.0 Hz, 1H, benzothiazole-CH), 7.35–7.45 (m, 3H, benzothiazole-CH & 2 Ar-CH), 7.65 (d, *J* = 8.0 Hz, 1H, benzothiazole-CH), 7.83 (d, *J* = 8.0 Hz, 1H, benzothiazole-CH), 8.07 (dd, *J* = 8.5 & 5.13 Hz, 2H, Ar-CH); anal. calcd for C_18_H_10_FN_5_OS (363.37): calc C% 59.50; H% 2.77; N% 19.27; S% 8.82; Calc C% 59.54; H% 2.74; N% 19.30; S% 8.79.

##### 4.1.2.4 2-(Benzo[*d*]thiazol-2-ylamino)-6-oxo-4-(*p*-tolyl)-1,6-dihydropyrimidine-5-carbonitrile (7d)

White solid (yield 72%), m.p. over 350 °C; IR (KBr, *ν* cm^−1^): 3029 (Ar-CH), 2918 (Aliph-CH), 2209 (CN), 1694 (C=O); ^1^H NMR (400 MHz, DMSO-*d*_6_): *δ* 2.43 (s, 3H, CH_3_), 7.21 (t, *J* = 7.6 Hz, 1H, benzothiazole-CH), 7.36–7.40 (m, 3H, benzothiazole-CH & 2 Ar-CH), 7.71 (d, *J* = 8.0 Hz, 1H, benzothiazole-CH), 7.83 (d, *J* = 7.6 Hz, 1H, benzothiazole-CH), 7.93 (d, *J* = 7.6 Hz, 2H, Ar-CH); ^13^C NMR (125 MHz, DMSO-*d*_6_): *δ* 21.6 (CH_3_), 119.4 (CN), 121.8, 122.7, 126.2, 129.0, 129.3, 129.4, 130.2, 130.5, 134.8, 136.3, 140.8, 163.6, 169.1 (Ar–C); anal. calcd for C_19_H_13_N_5_OS (359.40): calc C% 63.49; H% 3.65; N% 19.49; S% 8.92; found C% 63.53; H% 3.61; N% 19.51; S% 8.90.

#### 4.1.2 General procedure for the synthesis of 2-(benzo[*d*]thiazol-2-ylamino)-*N*-arylpyrimidin-5-carboxamide (13a–c)

In a solution of benzothiazole guanidine 3 (1 mmol) in dry 1,4-dioxane (10 mL) with potassium hydroxide (1 mmol), 2-cyano-3-(dimethylamino)-*N*-arylacrylamide 10a–c (10 mmol) was introduced. The resulting mixture was refluxed for 4 hours. Following cooling, the mixture was poured into ice water. The resulting solid product was filtered and subjected to crystallization from ethanol/DMF.

##### 4.1.2.1 4-Amino-2-(benzo[*d*]thiazol-2-ylamino)-*N*-phenylpyrimidine-5-carboxamide (13a)

White solid (yield 66.6%), m.p. over 350 °C; IR (KBr, *ν* cm^−1^): 3407, 3289 (NH_2_), 3236 (Ar-CH), 1633 (C=O); ^1^H NMR (400 MHz, DMSO-*d*_6_): *δ* 7.11 (t, *J* = 7.4 Hz, 1H, benzothiazole-CH), 7.23 (t, *J* = 7.6 Hz, 1H, benzothiazole-CH), 7.33–7.41 (m, 3H, Ar-CH), 7.66–7.71 (m, 3H, benzothiazole-CH & 2 Ar-CH), 7.88 (d, *J* = 7.8 Hz, 1H, benzothiazole-CH), 8.81 (s, 1H, pyrimidine-CH), 10.15 (br, 1H, NH), 11.74 (br, 2H, NH); anal. calcd for C_18_H_14_N_6_OS (362.41): calc C% 59.65; H% 3.89; N% 23.19; S% 8.85; found C% 59.61; H% 3.92; N% 23.16; S% 8.81.

##### 4.1.2.2 4-Amino-2-(benzo[*d*]thiazol-2-ylamino)-*N*-(4-methoxyphenyl)pyrimidine-5-carboxamide (13b)

White solid (yield 66.6%), m.p. over 350 °C; IR (KBr, *ν* cm^−1^): 3400, 3286 (NH_2_), 3237 (Ar-CH), 2833 (Aliph-CH), 1626 (C=O); ^1^H NMR (400 MHz, DMSO-*d*_6_): *δ* 3.75 (s, 3H, OCH_3_), 6.93 (d, *J* = 8.5 Hz, 2H, Ar-CH), 7.24 (t, *J* = 7.7 Hz, 1H, benzothiazole-CH), 7.40 (t, *J* = 7.8 Hz, 1H, benzothiazole-CH), 7.59 (d, *J* = 8.5 Hz, 2H, Ar-CH), 7.67 (d, *J* = 8.0 Hz, 1H, benzothiazole-CH), 7.88 (d, *J* = 8.0 Hz, 1H, benzothiazole-CH), 8.78 (s, 1H, pyrimidine-CH), 10.04 (s, 1H, NH), 11.73 (br, 2H, NH); ^13^C NMR (125 MHz, DMSO-*d*_6_): *δ* 55.6 (OCH_3_), 114.2, 120.2, 121.4, 122.8, 122.9, 126.2, 132.2, 132.9, 149.9, 156.1, 157.3, 162.9, 165.2 (Ar–C); anal. calcd for C_19_H_16_N_6_O_2_S (392.43): calc C% 58.15; H% 4.11; N% 21.42; S% 8.17; found C% 58.19; H% 4.09; N% 21.45; S% 8.18.

##### 4.1.2.3 4-Amino-2-(benzo[*d*]thiazol-2-ylamino)-*N*-(*p*-tolyl)pyrimidine-5-carboxamide (13c)

White solid (yield 66.6%), m.p. 344–345 °C; IR (KBr, *ν* cm^−1^): 3402, 3292 (NH_2_), 3204 (Ar-CH), 2960 (Aliph-CH), 1628 (C=O); ^1^H NMR (400 MHz, DMSO-*d*_6_): *δ* 2.28 (s, 3H, CH_3_), 7.10–7.15 (m, 3H, benzothiazole-CH & 2 Ar-CH), 7.30 (t, *J* = 7.7 Hz, 1H, benzothiazole-CH), 7.55–7.59 (m, 3H, benzothiazole-CH & 2 Ar-CH), 7.77 (d, *J* = 7.7 Hz, 1H, benzothiazole-CH), 8.77 (s, 1H, pyrimidine-CH), 9.99 (br, 1H, NH); anal. calcd for C_19_H_16_N_6_OS (376.43): calc C% 60.62; H% 4.28; N% 22.33; S% 8.52; found C% 60.65; H% 4.31; N% 22.30; S% 8.54.

#### 4.1.3 General procedure for the synthesis of 2-(benzo[*d*]thiazol-2-ylamino)-4-phenylpyrimidine (15a–c)

A mixture containing 2 mmol of the benzothiazole guanidine 3 and 3 mmol of acetylacetone 14a, ethyl benzoylacetone 14b or benzoylacetate 14c in 5 mL of triethyl orthoformate was heated for 30–60 minutes. After cooling, 10 mL of ethyl ether was added to the mixture and the resulting precipitate was separated by filtration, subjected to recrystallization in ethanol/DMF, and subsequently dried.

##### 4.1.3.1 1-(2-(Benzo[*d*]thiazol-2-ylamino)-4-methylpyrimidin-5-yl)ethanone (15a)

White solid (yield 66.6%), m.p. 310–311 °C; IR (KBr, *ν* cm^−1^): 3052 (Ar-CH), 2917 (Aliph-CH), 1674 (C=O); ^1^H NMR (400 MHz, DMSO-*d*_6_): *δ* 2.60 (s, 3H, CH_3_), 2.72 (s, 3H, CH_3_), 7.28 (t, *J* = 7.5 Hz, 1H, benzothiazole-CH), 7.42 (t, *J* = 7.5 Hz, 1H, benzothiazole-CH), 7.72 (d, t, *J* = 8.0 Hz, 1H, benzothiazole-CH), 7.97 (d, t, *J* = 7.5 Hz, 1H, benzothiazole-CH), 9.14 (s, 1H, pyrimidine-CH), 12.39 (s, 1H, NH); ^13^C NMR (125 MHz, DMSO-*d*_6_): *δ* 23.7 (2CH_3_), 113.8, 119.9, 121.5, 121.7, 122.8, 123.4, 126.2, 132.6, 157.1, 160.1, 167.9 (Ar–C); anal. calcd for C_14_H_12_N_4_OS (284.34): calc C% 59.14; H% 4.25; N% 19.70; S% 11.28; found C% 59.17; H% 4.28; N% 19.67; S% 11.25.

##### 4.1.3.2 (2-(Benzo[*d*]thiazol-2-ylamino)-4-methylpyrimidin-5-yl)(phenyl)methanone (15b)

White solid (yield 66.6%), m.p. 241–243 °C; IR (KBr, *ν* cm^−1^): 3054 (Ar-CH), 1686 (C=O); ^1^H NMR (400 MHz, DMSO-*d*_6_): *δ* 2.31 (s, 3H, CH_3_), 7.27 (t, *J* = 7.4 Hz, 1H, benzothiazole-CH), 7.42 (t, *J* = 7.4 Hz, 1H, benzothiazole-CH), 7.54–7.63 (m, 3H, Ar-CH), 7.65–7.76 (m, 3H, benzothiazole-CH & 2 Ar-CH), 7.96 (d, *J* = 8.0 Hz, 1H, benzothiazole-CH), 8.97 (s, 1H, pyrimidine-CH), 12.47 (br, 1H, NH); ^13^C NMR (125 MHz, DMSO-*d*_6_): *δ* 30.2 (CH_3_), 120.4, 121.8, 123.4, 126.1, 126.5, 129.1, 129.3, 129.6, 130.2, 130.9, 132.5, 137.6, 157.5, 159.4, 199.2 (Ar–C); anal. calcd for C_19_H_14_N_4_OS (346.41): calc C% 65.88; H% 4.07; N% 16.17; S% 9.26; found C% 65.86; H% 4.10; N% 16.15; S% 9.24.

##### 4.1.3.3 Ethyl 2-(benzo[*d*]thiazol-2-ylamino)-4-phenylpyrimidine-5-carboxylate (15c)

White solid (yield 66.6%), m.p. 211–212 °C; IR (KBr, *ν* cm^−1^): 3050 (Ar-CH), 2982 (Aliph-CH), 1724 (C=O); ^1^H NMR (400 MHz, DMSO-*d*_6_): *δ* 1.07 (t, *J* = 7.0 Hz, 3H, CH_3_), 4.14 (q, *J* = 7.0 Hz, 2H, CH_2_), 7.26 (t, *J* = 7.4 Hz, 1H, benzothiazole-CH), 7.43 (t, *J* = 7.7 Hz, 1H, benzothiazole-CH), 7.53–7.57 (m, 3H, Ar-CH), 7.67–7.74 (m, 3H, benzothiazole-CH & 2 Ar-CH), 7.94 (d, *J* = 7.9 Hz, 1H, benzothiazole-CH), 9.02 (s, 1H, pyrimidine-CH), 12.53 (br, 1H, NH); ^13^C NMR (125 MHz, DMSO-*d*_6_): *δ* 14.1 (CH_3_), 61.5 (CH_2_), 117.3, 120.4, 121.8, 123.4, 126.5, 128.6, 129.4, 130.6, 132.6, 137.6, 149.6, 157.9, 159.3, 161.0, 165.7 (Ar–C); anal. calcd for C_20_H_16_N_4_O_2_S (376.43): calc C% 63.81; H% 4.28; N% 14.88; S% 8.52; found C% 63.83; H% 4.30; N% 14.85; S% 8.55.

#### 4.1.4 General procedure for the synthesis of (*E*)-1-(2-(benzo[*d*]thiazol-2-ylamino)-4-methylpyrimidin-5-yl)-3-arylprop-2-en-1-one (17a–d)

Into a stirred solution of the respective 1-(2-(benzo[*d*]thiazol-2-ylamino)-4-methylpyrimidin-5-yl)ethanone 15a (1 mmol) in 10 mL of ethanol absolute, the corresponding benzaldehyde 16a–d (1 mmol) was added in the presence of NaOH, and the mixture was left at room temperature overnight. The reaction progress was monitored using TLC with AcOEt. Once the reaction was complete, the resulting precipitate was filtered off, and washed by hot ethanol.

##### 4.1.4.1 (*E*)-1-(2-(Benzo[*d*]thiazol-2-ylamino)-4-methylpyrimidin-5-yl)-3-phenylprop-2-en-1-one (17a)

Yellow solid (yield 66.6%), m.p. 241–242 °C; IR (KBr, *ν* cm^−1^): 2815 (Aliph-CH), 1663 (C=O); ^1^H NMR (400 MHz, DMSO-*d*_6_): *δ* 2.71 (s, 3H, CH_3_), 7.26 (t, *J* = 8.0 Hz, 1H, benzothiazole-CH), 7.39–7.51 (m, 4H, 3 Ar-CH & benzothiazole-CH), 7.68–7.75 (m, 3H, 2 CH=CH, benzothiazole-CH), 7.86 (d, *J* = 8.0 Hz, 2H, Ar-CH), 7.69 (d, *J* = 8.0 Hz, 1H, benzothiazole-CH), 9.17 (s, 1H, pyrimidine-CH), 12,47 (br, 1H, NH); anal. calcd for C_21_H_16_N_4_OS (372.44): calc C% 67.72; H% 4.33; N% 15.04; S% 8.61; found C% 67.69; H% 4.31; N% 15.02; S% 8.64.

##### 4.1.4.2 (*E*)-1-(2-(Benzo[*d*]thiazol-2-ylamino)-4-methylpyrimidin-5-yl)-3-(4-chlorophenyl)prop-2-en-1-one (17b)

Orange solid (yield 66.6%), m.p. 328–330 °C; IR (KBr, *ν* cm^−1^): 2826 (Aliph-CH), 1663 (C=O); ^1^H NMR (400 MHz, DMSO-*d*_6_): *δ* 2.64 (s, 3H, CH_3_), 7.00 (t, *J* = 7.5 Hz, 1H, benzothiazole-CH), 7.19 (t, *J* = 7.5 Hz, 1H, benzothiazole-CH), 7.45 (d, *J* = 8.0 Hz, 1H, benzothiazole-CH), 7.50 (d, *J* = 7.4 Hz, 2H, Ar-CH), 7.56 (d, *J* = 15.5 Hz, 1H, CH=CH), 7.67 (d, *J* = 7.7 Hz, 1H, benzothiazole-CH), 7.82 (d, *J* = 15.5 Hz, 1H, CH=CH), 7.90 (d, *J* = 7.4 Hz, 2H, Ar-CH), 9.06 (s, 1H, pyrimidine-CH); anal. calcd for C_21_H_15_ClN_4_OS (406.89): calc C% 61.99; H% 3.72; N% 13.77; S% 7.88; found C% 61.97; H% 3.73; N% 13.79; S% 7.91.

##### 4.1.4.3 (*E*)-1-(2-(Benzo[*d*]thiazol-2-ylamino)-4-methylpyrimidin-5-yl)-3-(4-bromophenyl)prop-2-en-1-one (17c)

Yellow solid (yield 66.6%), m.p. 282–283 °C; IR (KBr, *ν* cm^−1^): 2825 (Aliph-CH), 1661 (C=O); ^1^H NMR (400 MHz, DMSO-*d*_6_): 2.72 (s, 3H, CH_3_), 7.27 (t, *J* = 7.5 Hz, 1H, benzothiazole-CH), 7.42 (t, *J* = 7.5 Hz, 1H, benzothiazole-CH), 7.63–7.77 (m, 5H, 2 Ar-CH, 2 CH=CH & benzothiazole-CH), 7.84 (d, *J* = 8.4 Hz, 2H, Ar-CH), 7.97 (d, *J* = 7.6 Hz, 1H, benzothiazole-CH), 9.19 (s, 1H, pyrimidine-CH), 12.42 (br, 1H, NH); anal. calcd for C_21_H_15_BrN_4_OS (452.01): calc C% 55.88; H% 3.35; N% 12.41; found C% 55.90; H% 3.32; N% 12.38.

##### 4.1.4.4 (*E*)-1-(2-(Benzo[*d*]thiazol-2-ylamino)-4-methylpyrimidin-5-yl)-3-(4-methoxyphenyl)prop-2-en-1-one (17d)

Yellow solid (yield 66.6%), m.p. over 350 °C; IR (KBr, *ν* cm^−1^): 2834 (Aliph-CH), 1685 (C=O); ^1^H NMR (400 MHz, DMSO-*d*_6_): *δ* 2.64 (s, 3H, CH_3_), 3.82 (s, 3H, OCH_3_), 6.99–7.05 (m, 3H, 2 Ar-CH & benzothiazol-H), 7.22 (t, *J* = 7.6 Hz, 1H, benzothiazole-CH), 7.48 (d, *J* = 7.6 Hz, 1H, benzothiazole-CH), 7.56 (d, *J* = 12.4 Hz, 1H, CH=CH), 7.62 (d, *J* = 12.4 Hz, 1H, CH=CH), 7.70 (d, *J* = 7.6 Hz, 1H, benzothiazole-CH), 7.81 (d, *J* = 8.4 Hz, 2H, Ar-CH), 9.03 (s, 1H, pyrimidine-CH); anal. calcd for C_22_H_18_N_4_O_2_S (402.47): calc C% 65.65; H% 4.51; N% 13.92; S% 7.97; found C% 65.60; H% 4.53; N% 13.97; S% 8.01.

#### 4.1.5 General procedure for the synthesis of 2-(benzo[*d*]thiazol-2-ylamino)-4-phenylpyrimidine-5-carbohydrazide (18)

A mixture comprising ethyl 2-(benzo[*d*]thiazol-2-ylamino)-4-phenylpyrimidine-5-carboxylate 18 (1 mmol) and 80% hydrazine hydrate (5 mL) was subjected to reflux for 6 hours. The solid product obtained upon cooling was filtered and recrystallized from ethanol.

White solid (yield 66.6%), m.p. 294–295 °C; IR (KBr, *ν* cm^−1^): 3285 (NH_2_), 1628 (C=O); ^1^H NMR (400 MHz, DMSO-*d*_6_): *δ* 4.51 (br, 2H, NH_2_), 7.26 (t, *J* = 8.0 Hz, 1H, benzothiazole-CH), 7.22 (t, *J* = 8.0 Hz, 1H, benzothiazole-CH), 7.51–7.65 (m, 3H, Ar-CH), 7.70 (d, *J* = 8.0 Hz, 1H, benzothiazole-CH), 7.87–7.91 (m, 2H, Ar-CH), 7.96 (d, *J* = 8.0 Hz, 1H, benzothiazole-CH), 8.65 (s, 1H, pyrimidine-CH), 9.66 (br, 1H, NH), 12.27 (br, 1H, NH); ^13^C NMR (100 MHz, DMSO-*d*_6_): *δ* 120.3, 121.8, 123.2, 126.4, 129.0, 129.3, 130.9, 132.4, 136.9, 149.8, 157.3, 158.7, 159.6, 164.0, 165.9 (Ar–C); anal. calcd for C_18_H_14_N_6_OS (362.09): calc C% 59.65; H% 3.89; N% 23.19; S% 8.85; found C% 59.68; H% 3.93; N% 23.15; S% 8.80.

#### 4.1.6 General procedure for the synthesis of 2-(benzo[*d*]thiazol-2-ylamino)-4/6-(methylthio)pyrimidine 20, 22, & 24

A mixture of benzothiazole guanidine 3 (1 mmol) and (2,2-diisocyanoethene-1,1-diyl)bis(methylsulfane) 19, ethyl 2-isocyano-3,3-bis(methylthio)acrylate 21 or 2-(benzo[*d*]thiazol-2-yl)-3-(dimethylamino) acrylonitrile 23 (1.2 mmol) in dry dioxane (20 mL) with potassium hydroxide (1.3 mmol) was refluxed for 2–4 hours, as determined by TLC. After cooling, the reaction mixture was transferred to ice acidified with HCl. The resulting solid product was separated by filtration, rinsed with ethyl acetate, followed by ethanol, yielding the corresponding products 23, 25 and 27 respectively.

##### 4.1.6.1 4-Amino-2-(benzo[*d*]thiazol-2-ylamino)-6-(methylthio)pyrimidine-5-carbonitrile (20)

Yellow solid (yield 66.6%), m.p. over 350 °C; IR (KBr, *ν* cm^−1^): 3379–3238 (NH_2,_ NH), 3004 (Ar-CH), 2769 (Aliph-CH), 2208 (CN); ^1^H NMR (400 MHz, DMSO-*d*_6_): *δ* 2.72 (s, 3H, SCH_3_), 7.25 (t, *J* = 7.5 Hz, 1H, benzothiazole-CH), 7.40 (t, *J* = 7.6 Hz, 1H, benzothiazole-CH), 7.60–7.70 (m, 3H, NH_2_ & benzothiazol-H), 7.89 (d, *J* = 7.8 Hz, 1H, benzothiazole-CH), 11.82 (br, 1H, NH); ^13^C NMR (125 MHz, DMSO-*d*_6_): *δ* 40.5 (SCH_3_), 115.6 (CN), 120.4, 121.6, 123.3, 126.4, 132.8, 149.8, 163.1, 167.7, 174.4 (Ar–C); anal. calcd for C_13_H_10_N_6_S_2_ (314.39): calc C% 49.66; H% 3.21; N% 26.73; S% 20.40; found C% 49.70; H% 3.18; N% 26.77; S% 20.44.

##### 4.1.6.2 2-(Benzo[*d*]thiazol-2-ylamino)-4-(methylthio)-6-oxo-1,6-dihydropyrimidine-5-carbonitrile (22)

Brown solid (yield 66.6%), m.p. over 350 °C; IR (KBr, *ν* cm^−1^): 3378 (NH), 3055 (Ar-CH), 2924 (Aliph-CH), 2198 (CN), 1663 (C=O); ^1^H NMR (400 MHz, DMSO-*d*_6_): *δ* 2.69 (s, 3H, SCH_3_), 7.11 (t, *J* = 7.6 Hz, 1H, benzothiazole-CH), 7.28 (t, *J* = 7.7 Hz, 1H, benzothiazole-CH), 7.54 (d, *J* = 8.0 Hz, 1H, benzothiazole-CH), 7.76 (d, *J* = 8.0 Hz, 1H, benzothiazole-CH), 10.84 (br, 1H, NH); ^13^C NMR (125 MHz, DMSO-*d*_6_): *δ* 40.4 (SCH_3_), 118.4 (CN), 119.7, 121.1, 1212, 122.0, 125.5, 133.5, 150.7 (Ar,C); anal. calcd for C_13_H_9_N_5_OS_2_ (315.37): calc C% 49.51; H% 2.88; N% 22.21; S% 20.33; found C% 49.57; H% 2.90; N% 22.25; S% 20.30.

##### 4.1.6.3 *N* ^2^,5-Bis(benzo[*d*]thiazol-2-yl)pyrimidine-2,4-diamine (24)

Buff solid (yield 66.6%), m.p. 346–347 °C; IR (KBr, *ν* cm^−1^): 3463, 3266 (NH_2_, NH), 3057 (Ar-CH); ^1^H NMR (400 MHz, DMSO-*d*_6_): *δ* 7.26 (t, *J* = 7.6 Hz, 1H, benzothiazole-CH), 7.39–7.47 (m, 2H, benzothiazol-H), 7.55 (t, *J* = 7.6 Hz, 1H, benzothiazole-CH), 7.69 (d, *J* = 8.0 Hz, 1H, benzothiazole-CH), 7.90 (d, *J* = 7.8 Hz, 1H, benzothiazole-CH), 8.06 (d, *J* = 8.0 Hz, 1H, benzothiazole-CH), 8.13 (d, *J* = 8.0 Hz, 1H, benzothiazole-CH), 8.78 (s, 1H, pyrimidine-CH), 11.8 (br, 1H, NH); anal. calcd for C_18_H_12_N_6_S_2_ (376.46): calc C% 57.43; H% 3.21; N% 22.32; S% 17.04; found C% 57.47; H% 3.17; N% 22.38; S% 17.10.

### 4.2. Anticancer evaluation

#### 4.2.1 Preparation of pyrimidine derivatives provided compound

A 100 μmol mL^−1^ stock solution was created by reconstituting the dried extract in an appropriate volume of DMSO, based on the molecular weight of each compound, followed by 5 seconds of sonication. This stock solution was aliquoted and stored at −20 °C until needed. Final test compound concentrations for all experiments were prepared by diluting the stock with the medium. The control cells received the carrier solvent (0.1% DMSO).

#### 4.2.2 Screening of the cytotoxic effect of different pyrimidine derivatives provided

##### 4.2.2.1 Cell line

The Human adenocarcinoma breast cancer cells “MCF7”, Human colorectal cancer cell lines “HCT116”, and the human hepatocellular carcinoma cells “HepG2” were obtained from Nawah Scientific, Cairo, Egypt, in 25 mL T-culture flask. The cells were prepared for experiments using the conventional trypsinization procedure with trypsin/EDTA. Cells were subculture “passaging” in Dulbecco's Modified Eagle Medium with high glucose (4.5 g L^−1^), l-glutamine and sodium pyruvate, supplemented with 10% fetal bovine serum (FBS) and 1% antibiotic/antimycotic mixture (Gibco, Thermosientific, Germany) containing 10% fetal bovine serum (FBS) (Gibco, Thermosientific, Germany) and 1% of penicillin G sodium (10.000 UI), streptomycin (10 mg) and amphotericin B (25 μg) (PSA) (Gibco, Thermosientific, Germany). Culture Flasks were incubated at 37 °C in an atmosphere of 5% CO_2_, monitored until it reaches the 70% confluence, then the cells were harvested by conventional trypsinization using 0.25% Trypsin EDTA (Gibco, Thermosientific, Germany). Cells from the 4th passages were used for the downstream experiment.

##### 4.2.2.2 Assessment of cell viability by cell proliferation assay (MTT) 48 hours after culture

One day before conducting the experiment, the cancer cells were seeded in 96-well culture plate. 8 × 10^3^ cells per well were seeded in 200 μL of DMEM, supplemented with 10% FBS and 1% of penicillin G sodium (10.000 UI), streptomycin (10 mg) and amphotericin B (25 μg) (PSA) (Gibco, Thermosientific, Germany). Culture plates were incubated at 37 °C in an atmosphere of 5% CO_2_ for 24 hours for attach of cells. On the next day, a constant concentration of 100 μmol mL^−1^, was prepared for each compound and used for treatment of cells. In addition, the carrier solvent (0.1% DMSO) was used for control cells. The 5-Fluorouracil was used as positive control for the three cancer cell lines with a concentration 140.0 μmol mL^−1^,^36^ 1.1 μmol mL^−1^,^37^ and 125 μg mL^−1^,^38^ on MCF7, HepG2, and HCT116; respectively. Cells were maintained at 37 °C in an atmosphere of 5% CO_2_ for 48 hours. At the end of incubation, the cell proliferation assay was performed using the Vybrant® MTT Cell Proliferation Assay Kit, cat no: M6494 (Thermo Fisher, Germany). 100 μL of media was removed from and replaced by fresh media. Twenty μL of 3-(4,5-dimethylthiazol-2-yl)-2,5-diphenyltetrazolium bromide (MTT) solution (1 mg mL^−1^), Invitrogen, ThermoScientific, Germany was added to each well and the plates were incubated at 37 °C and 5% CO_2_ for four hours. Finally, the MTT solution was removed and 100 μL of sodium dodecyl sulphate with hydrochloric acid (SDS-HCL) was added to the wells. Cell viability was determined by measuring the optical density at 570 nm on a spectrophotometer (ELx 800; Bio-Tek Instruments Inc., Winooski, VT, USA).

#### 4.2.3 Determination of the half maximal cytotoxic effect (IC_50_) half maximal cytotoxic effect (IC_50_) of pyrimidine derivatives on three cancer cell lines

One day before conducting the experiment, the cancer cells were seeded in 96-well culture plate. 8 × 10^3^ cells per well of cells were seeded in 200 μL of Dulbecco's Modified Eagle Medium (DMEM) (Gibco, Thermosientific, Germany) with high glucose (4.5 g L^−1^), l-glutamine and sodium pyruvate, containing 10% fetal bovine serum (FBS) (Gibco, Thermosientific, Germany) and 1% of penicillin G sodium (10.000 UI), streptomycin (10 mg) and amphotericin B (25 μg) (PSA) (Gibco, Thermosientific, Germany). Culture plates were incubated at 37 °C in an atmosphere of 5% CO_2_ for 24 hours to reach the 70% confluence. On the next day, a serial concentration of each tested compound was performed “100 μmol mL^−1^, 10 μmol mL^−1^, 1.0 μmol mL^−1^, 0.1 μmol mL^−1^, and 0.01 μmol mL^−1^” were prepared and used for treatment of cells. In addition, the carrier solvent (0.1% DMSO) was used for control cells.

The treated cancer cells were incubated at 37 °C in an atmosphere of 5% CO_2_ for 72 hours, then the cell viability was tested by MTT assay and the IC_50_ was calculated. At the end of incubation time, the cell cytotoxicity assay was performed using the Vybrant® MTT Cell Proliferation Assay Kit, cat no: M6494 (Thermo Fisher, Germany). 100 μL of media was removed and replaced by new media. Twenty μL of 4,5-dimethylthiazol-2-yl)-2,5-diphenyltetrazolium bromide (MTT) solution (1 mg mL^−1^) (Invitrogen, ThermoScientific, Germany) was added to each well and the plates were incubated at 37 °C and 5% CO_2_ for four hours. Finally, the MTT solution was removed and 100 μL of sodium dodecyl sulphate with hydrochloric acid (SDS-HCL) was added to the wells. Cell viability was determined by measuring the optical density at 570 nm on a spectrophotometer (ELx 800; Bio-Tek Instruments Inc., Winooski, VT, USA).

#### 4.2.4 Calculation of IC_50_ of pyrimidine derivatives on three cancer cell lines

At the end of each time interval, the cell proliferation assay was conducted, and the % of viability was determined which represent the cytotoxic effect of serial doses of each compound. The *XY* curve was plotted to illustrate the relation between the log dose of (inhibitor) *versus* the normalized response. The best fit point was determined by linear regression analysis. Calculation of half maximal stimulatory concentration (IC_50_). The IC_50_ was calculated using the GraphPad prism software, Prism 9, version 9.1.0 (221). The determination of IC_50_ for each group was calculated based on concentration–response curves of analyzed cellular metabolic activity, which were normalized to untreated cells.

### 4.3. Drug likeness predictions and physicochemical–pharmacokinetic/ADMET properties

Drug-likeness, a qualitative concept integral to drug design, plays a key role in predicting drug-like properties. Essential factors such as solubility, permeability, transporter effects, and metabolic stability significantly influence the success of drug candidates by impacting oral bioavailability, toxicity, metabolism, clearance, and *in vitro* pharmacology. To assess the drug-likeness of the synthesized compounds, five distinct filters—Lipinski,^39^ Veber,^40^ Muegge,^41^ Ghose,^42^ and Egan^43^ rules—were employed, along with considerations for bioavailability. Furthermore, drug-likeness scores were determined using both the Molsoft software and the SwissADME program.

### 4.4. Molecular docking study

The molecular studies were conducted using the Molecular Operating Environment (MOE 2014). Ligand molecules were drawn using the builder molecule, and their energy was minimized. The minimization process continued until an rmsd gradient of 0.01 kcal mol^−1^ was achieved, employing the MMFF94X force field, with automatic calculation of partial charges. Docking simulations utilized the crystal structure of the PTK receptor in complex with 1N1 from the Protein Data Bank (PDB ID: 2GQG). Ligands bound to the structure were excluded, and the MOE protonate 3D application was employed to add missing hydrogens and accurately assign ionization states. For the generation of the active site, the MOE-Alpha site finder was utilized, and the obtained alpha spheres were employed to create dummy atoms. Subsequently, ligands were docked into the active sites using MOE-Dock. The ranking of optimized poses was determined using GBVI/WSA DG free-energy estimates, and the docking poses underwent visual examination. The final step involved investigating interactions with binding pocket residues.

## Author contributions

Toka I. Ismail carried out the chemical experiments and analyzed the resulting structures. Nashwa El-Khazragy led and conducted the analysis of the anticancer study. Rasha A. Azzam developed and designed the work, carried out the docking analysis, and evaluated the ADMET properties. All authors contributed to manuscript development.

## Conflicts of interest

There are no conflicts to declare.

## Supplementary Material

RA-014-D4RA01874E-s001
