# Supplementary material for: In the pursuit of novel therapeutic agents: synthesis, anticancer evaluation, and physicochemical insights of novel pyrimidine-based 2-aminobenzothiazole derivatives
Source: RSC Adv. 2024 May 20;14(23):16332–48. doi: 10.1039/d4ra01874e (PMC11103668; doi:10.1039/d4ra01874e)
Supplement: RA-014-D4RA01874E-s001 [file RA-014-D4RA01874E-s001.pdf]

Electronic Supplementary Material (ESI) for RSC Advances.
This journal is © The Royal Society of Chemistry 2024

In the Pursuit of Novel Therapeutic Agents: Synthesis, Anticancer Evaluation, and Physicochemical Insights of Novel Pyrimidine-based 2-Aminobenzothiazole Derivatives

Toka I. Ismail[1], Nashwa El-Khazragy[2,3], Rasha A. Azzam[1]

[1] *Chemistry Department, Faculty of Science, Helwan University, Cairo, Egypt, 11795*
[2] *Department of Clinical Pathology-Hematology, Ain Shams Medical Research Institute (MASRI), Faculty of Medicine, Ain Shams University, Cairo 11566, Egypt*
[3] *Department of Genetics and Molecular Biology, Egypt Center for Research and Regenerative Medicine (ECRRM), Cairo11599, Egypt*

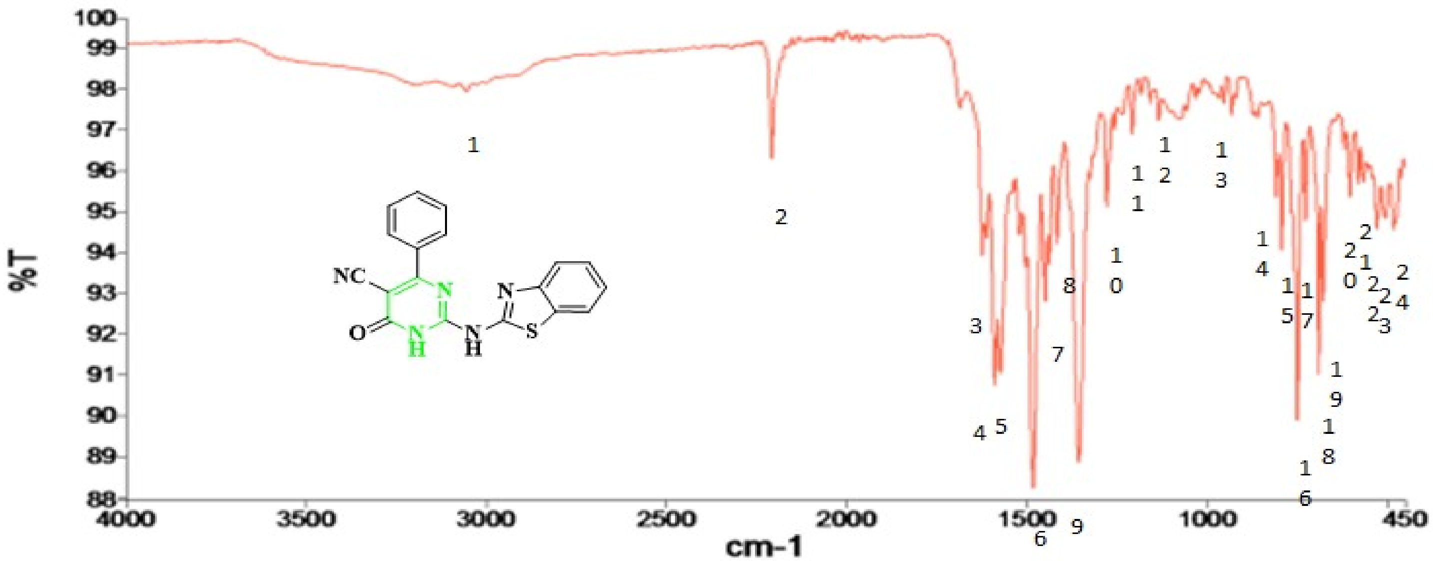

No.	Position	Intinsty	No.	Position	Intinsty	No.	Position	Intinsty	No.	Position	Intinsty
1	3058.10	97.97	7	1448.91	92.81	13	930.39	97.39	19	676.42	92.81
2	2209.58	96.33	8	1415.83	94.22	14	807.79	95.38	20	601.72	95.36
3	1624.58	93.94	9	1355.50	88.83	15	791.92	94.07	21	579.27	95.69
4	1589.13	90.74	10	1278.71	95.12	16	748.84	89.87	22	526.86	94.58
5	1572.21	91.02	11	1207.81	96.92	17	725.09	94.79	23	503.50	94.84
6	1482.98	88.20	12	1133.87	97.27	18	689.24	91.01	24	480.26	94.55

Figure 1. IR spectrum of compound **7a**

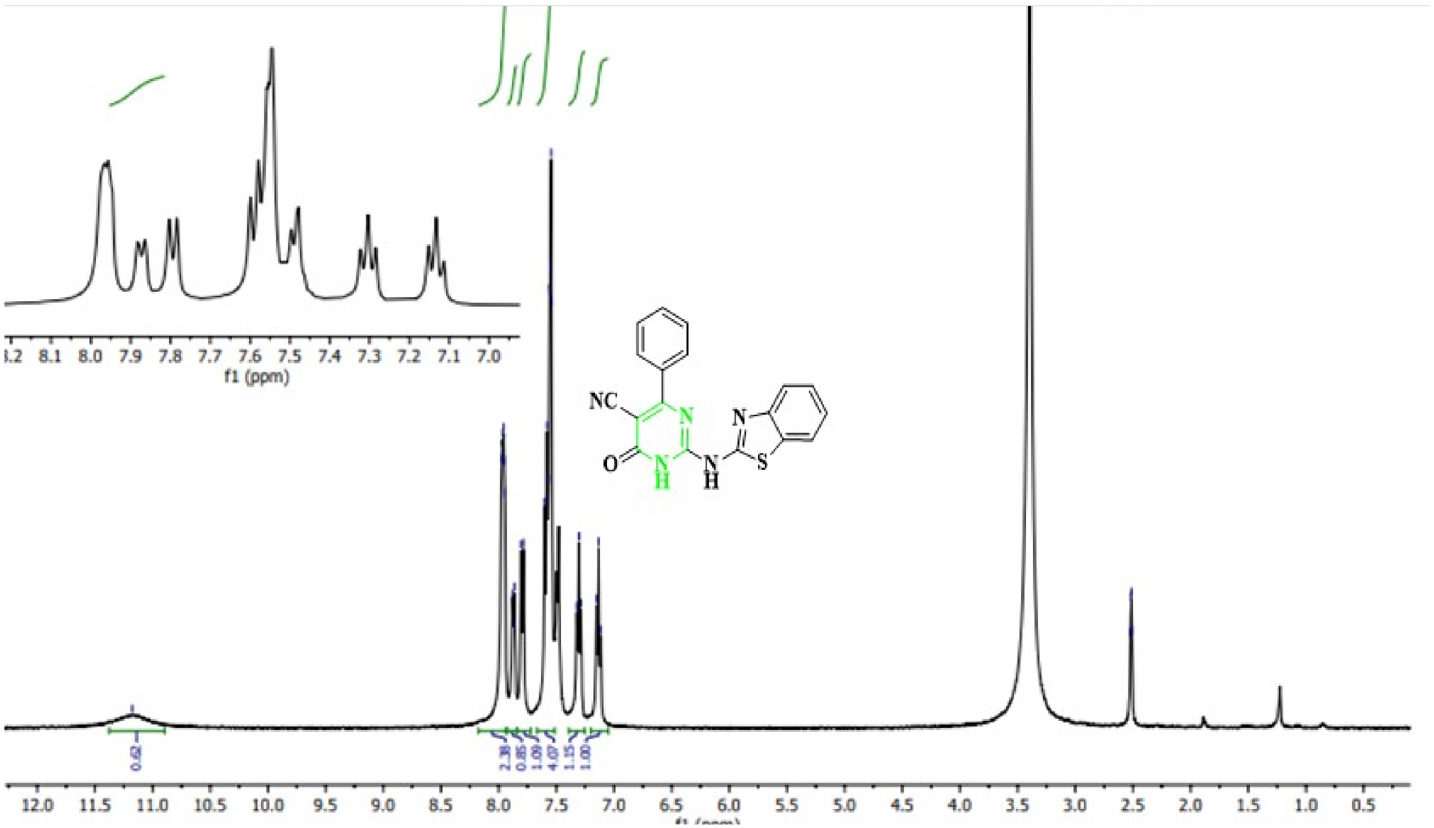

Figure 2. ^{1}H NMR spectrum of compound **7a**

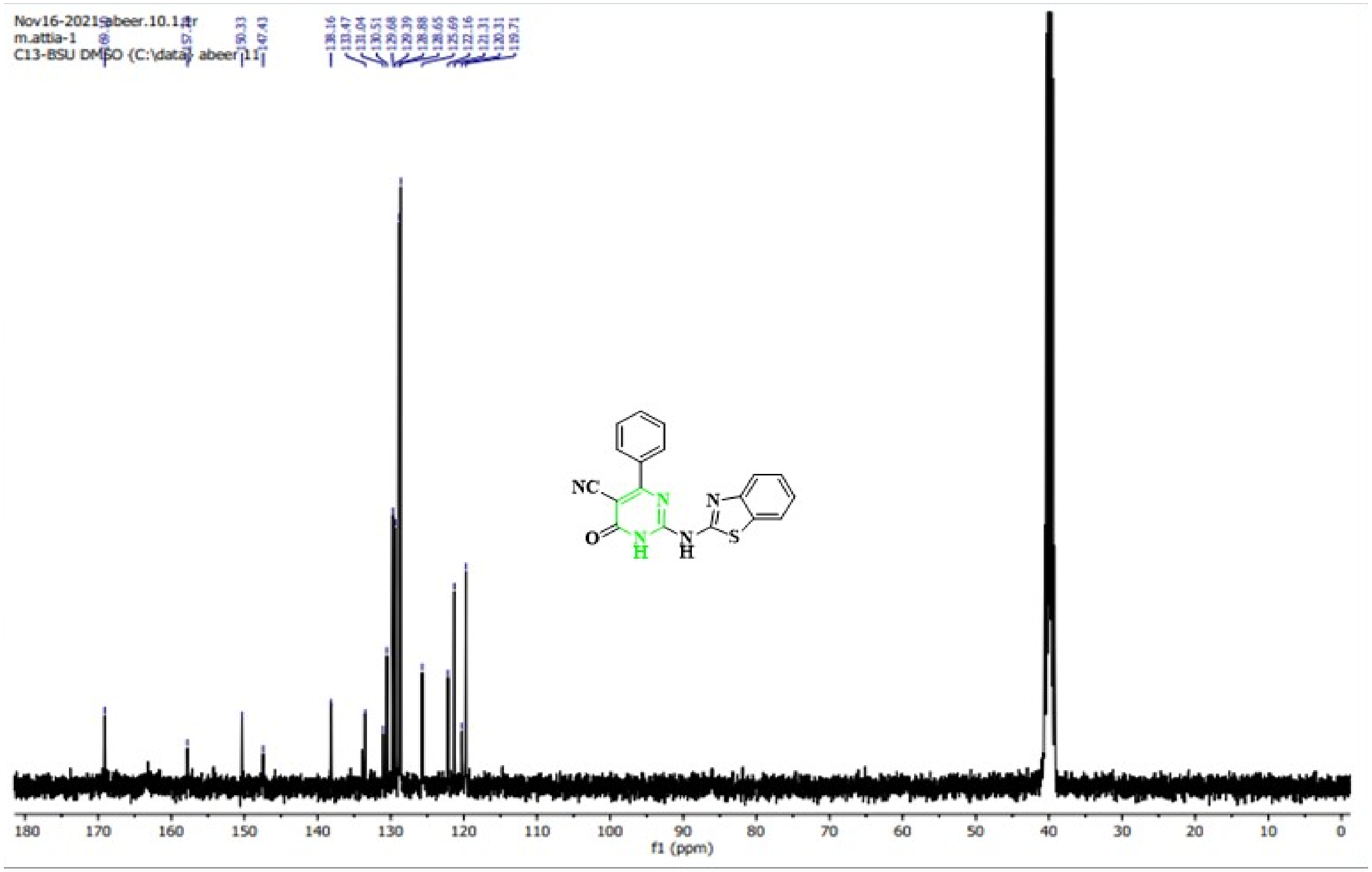

Figure 3. ^{13}C NMR spectrum of compound **7a**

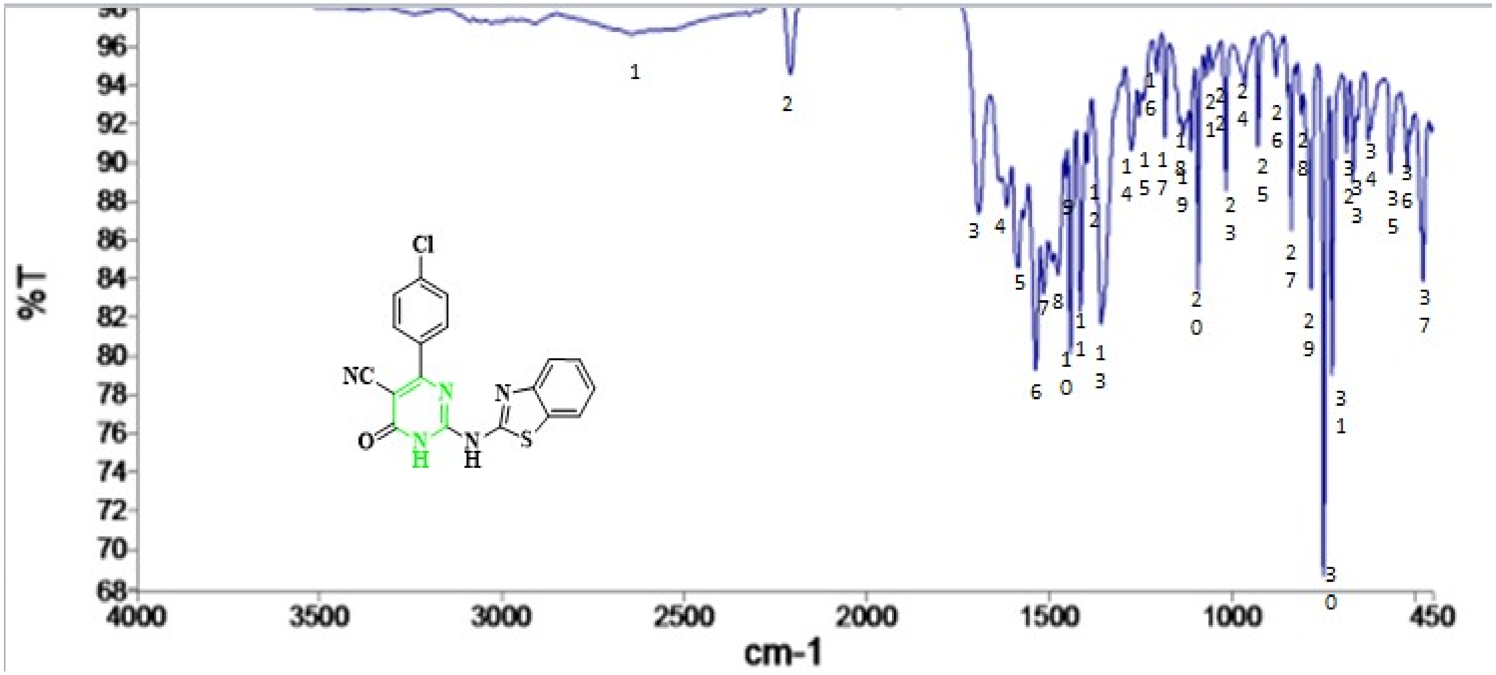

No.	Position	Intinsty	No.	Position	Intinsty	No.	Position	Intinsty	No.	Position	Intinsty	No.	Position	Intinsty
1	2640.09	96.75	9	1456.16	89.26	17	1183.08	91.32	25	927.78	90.90	33	667.05	88.99
2	2210.17	94.63	10	1442.97	80.15	18	1133.67	91.47	26	880.01	94.48	34	626.14	91.20
3	1694.03	87.39	11	1415.59	82.34	19	1111.97	90.65	27	837.80	86.55	35	564.82	89.48
4	1617.26	87.75	12	1396.75	90.08	20	1092.21	83.41	28	810.05	92.58	36	519.40	89.66
5	1585.69	84.58	13	1358.93	81.68	21	1070.58	94.49	29	783.17	83.49	37	474.72	83.89
6	1537.44	79.31	14	1275.38	90.65	22	1054.71	94.84	30	748.44	68.58			
7	1516.39	83.25	15	1255.09	92.44	23	1015.91	88.61	31	725.22	79.01			
8	1475.74	84.22	16	1205.88	94.71	24	964.39	94.00	32	685.98	90.56			

Figure 4. IR spectrum of compound **7b**

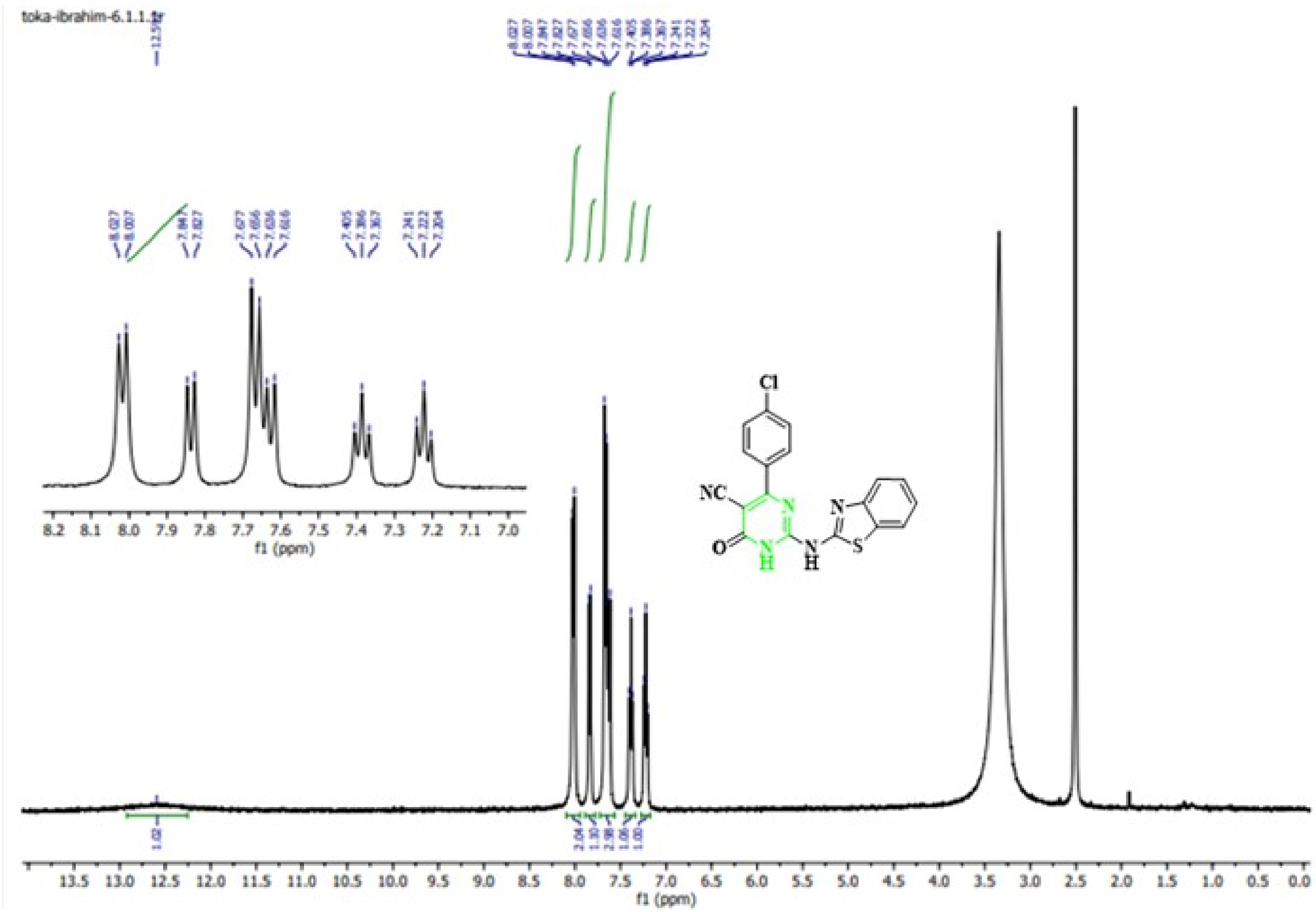

Figure 5. ^{1}H NMR spectrum of compound **7b**

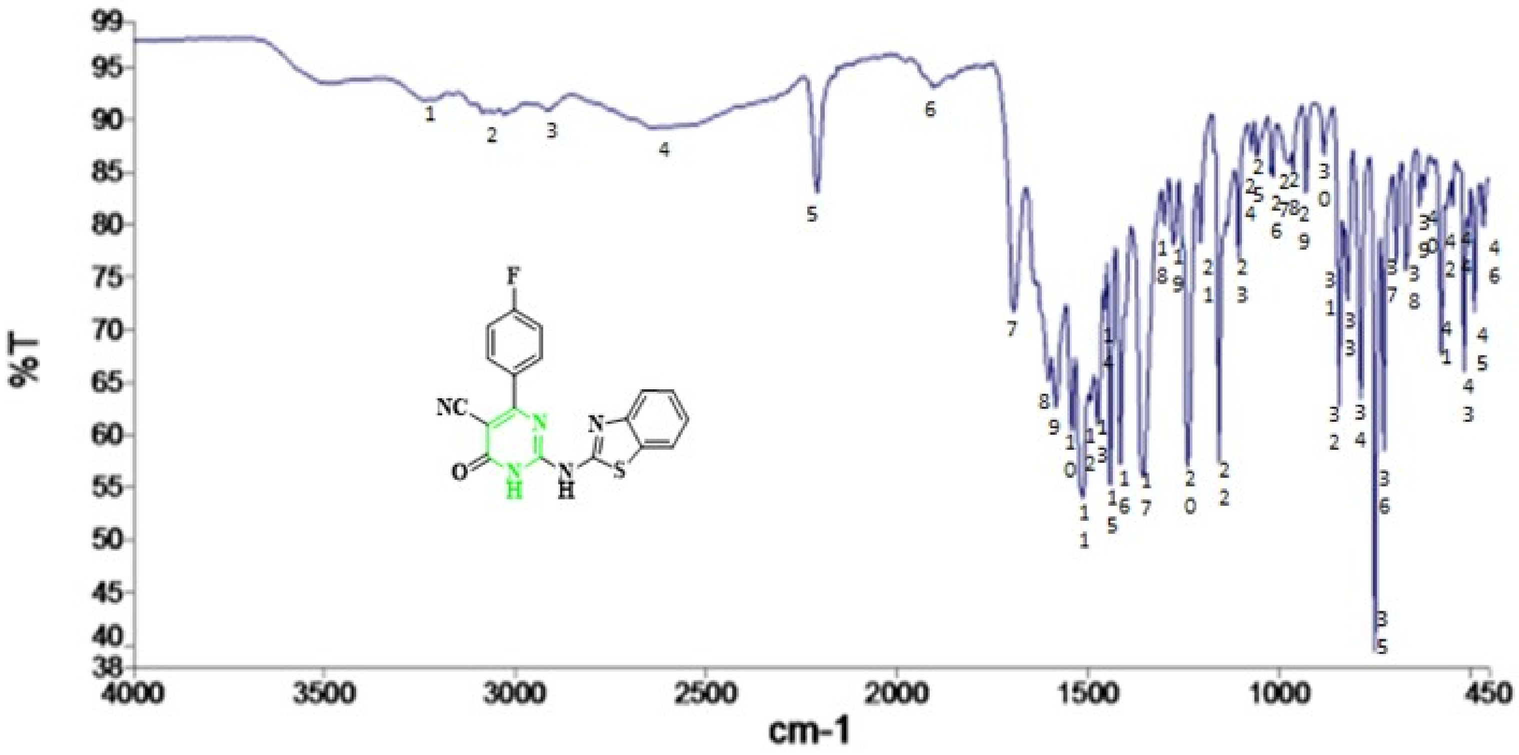

No.	Position	Intinsty	No.	Position	Intinsty	No.	Position	Intinsty	No.	Position	Intinsty	No.	Position	Intinsty
1	3239.76	92.00	11	1513.60	54.01	20	1238.47	56.90	29	928.22	83.18	38	666.40	75.77
2	3031.77	90.65	12	1492.31	63.22	21	1205.57	78.37	30	881.30	86.76	39	630.09	81.86
3	2917.33	91.10	13	1474.08	60.91	22	1157.00	57.39	31	840.59	62.81	40	617.49	83.53
4	2639.74	89.38	14	1456.38	71.98	23	1104.99	76.78	32	829.10	79.09	41	573.32	67.70
5	2210.16	83.19	15	1441.88	55.19	24	1071.04	87.17	33	819.40	72.81	42	543.94	81.88
6	1906.98	93.18	16	1415.16	57.16	25	1055.98	86.58	34	784.77	63.45	43	513.85	66.07
7	1694.14	71.78	17	1355.10	55.92	26	1014.53	84.68	35	747.44	39.26	44	502.33	79.88
8	1602.35	65.20	18	1300.37	80.09	27	975.76	85.95	36	724.15	58.52	45	486.76	71.77
9	1583.81	62.60	19	1275.81	78.19	28	961.02	85.19	37	691.32	76.60	46	462.71	79.98
10	1542.22	60.39												

Figure 6. IR spectrum of compound **7c**

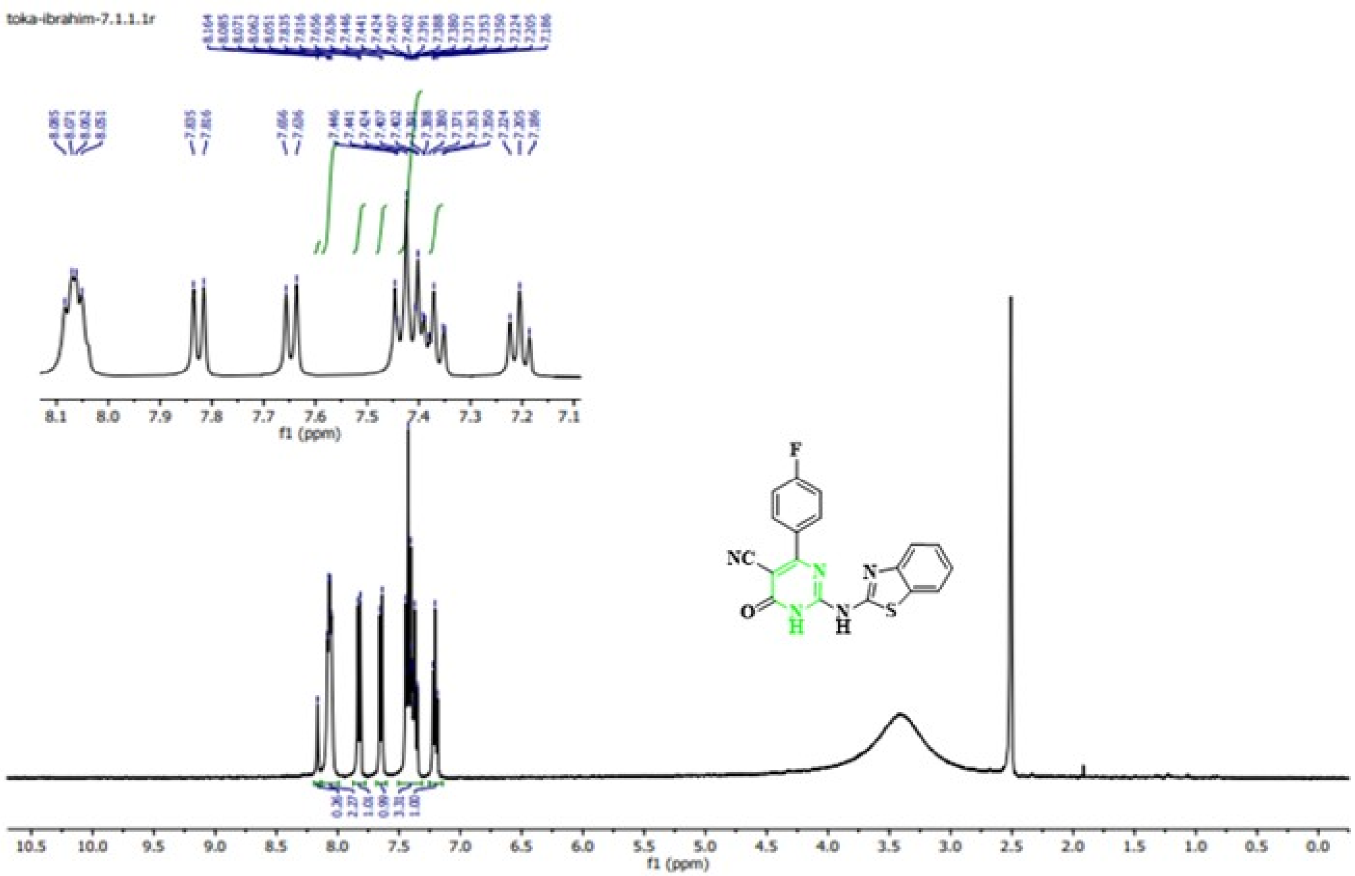

Figure 7. ^{1}H NMR spectrum of compound **7c**

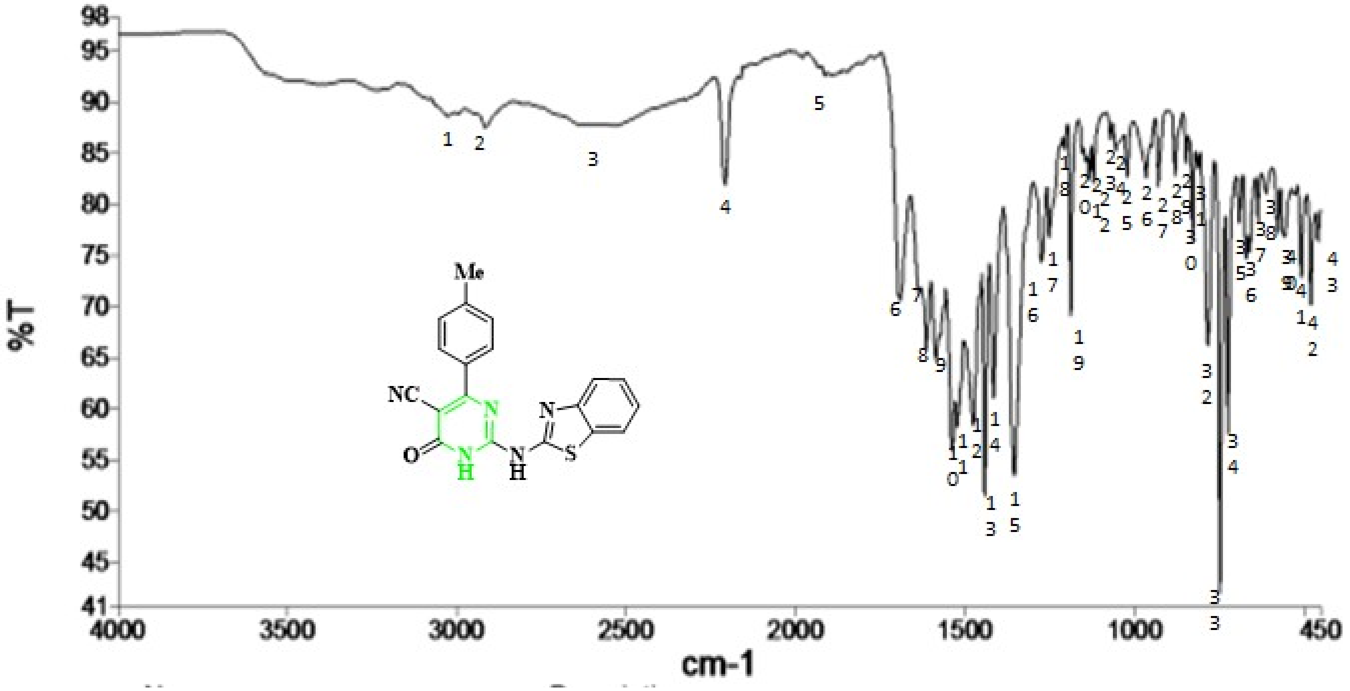

No.	Position	Intinsty	No.	Position	Intinsty	No.	Position	Intinsty	No.	Position	Intinsty	No.	Position	Intinsty
1	3029.19	88.72	10	1536.70	55.88	19	1187.88	69.15	28	847.10	84.12	37	634.94	78.32
2	2918.35	87.62	11	1522.81	58.23	20	1133.86	82.57	29	828.07	76.48	38	610.34	81.19
3	2544.06	87.84	12	1475.47	58.39	21	1120.02	82.17	30	812.04	83.70	39	577.55	77.16
4	2209.86	81.98	13	1441.61	51.42	22	1070.30	86.46	31	783.02	66.23	40	555.76	76.92
5	1912.80	92.70	14	1415.79	61.01	23	1055.44	85.34	32	746.82	42.03	41	507.21	72.90
6	1692.31	70.65	15	1354.16	53.42	24	1021.13	82.76	33	723.88	57.60	42	478.58	70.18
7	1637.14	71.38	16	1274.52	74.41	25	965.32	82.67	34	690.20	78.24	43	456.14	76.34
8	1614.38	65.84	17	1250.83	76.80	26	929.25	81.8	35	670.10	74.75			
9	1585.30	64.80	18	1206.37	85.49	27	880.07	82.86	36	659.41	75.41			

Figure 8. IR spectrum of compound **7d**

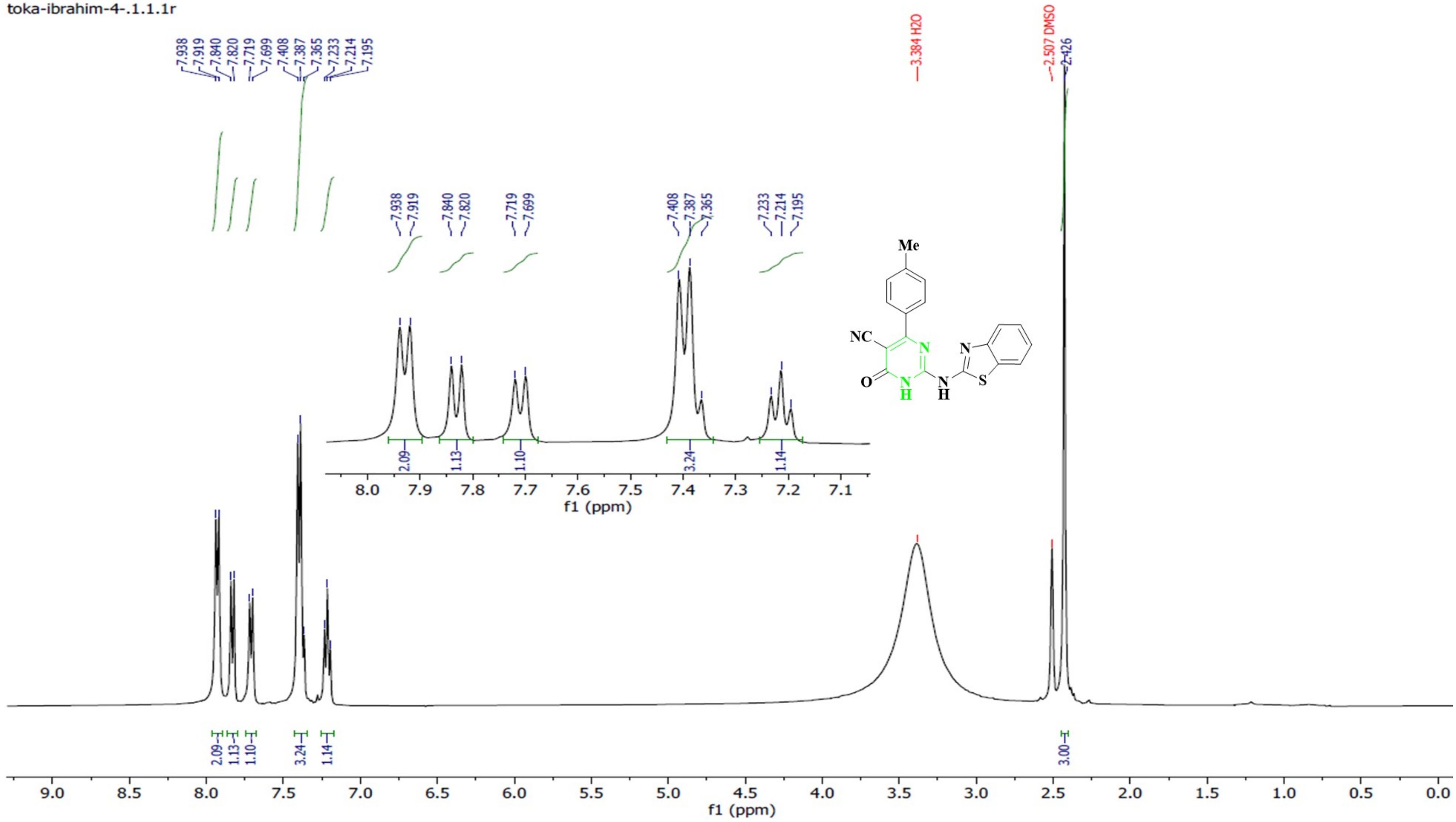

Figure 9. ^{1}H NMR spectrum of compound **7d**

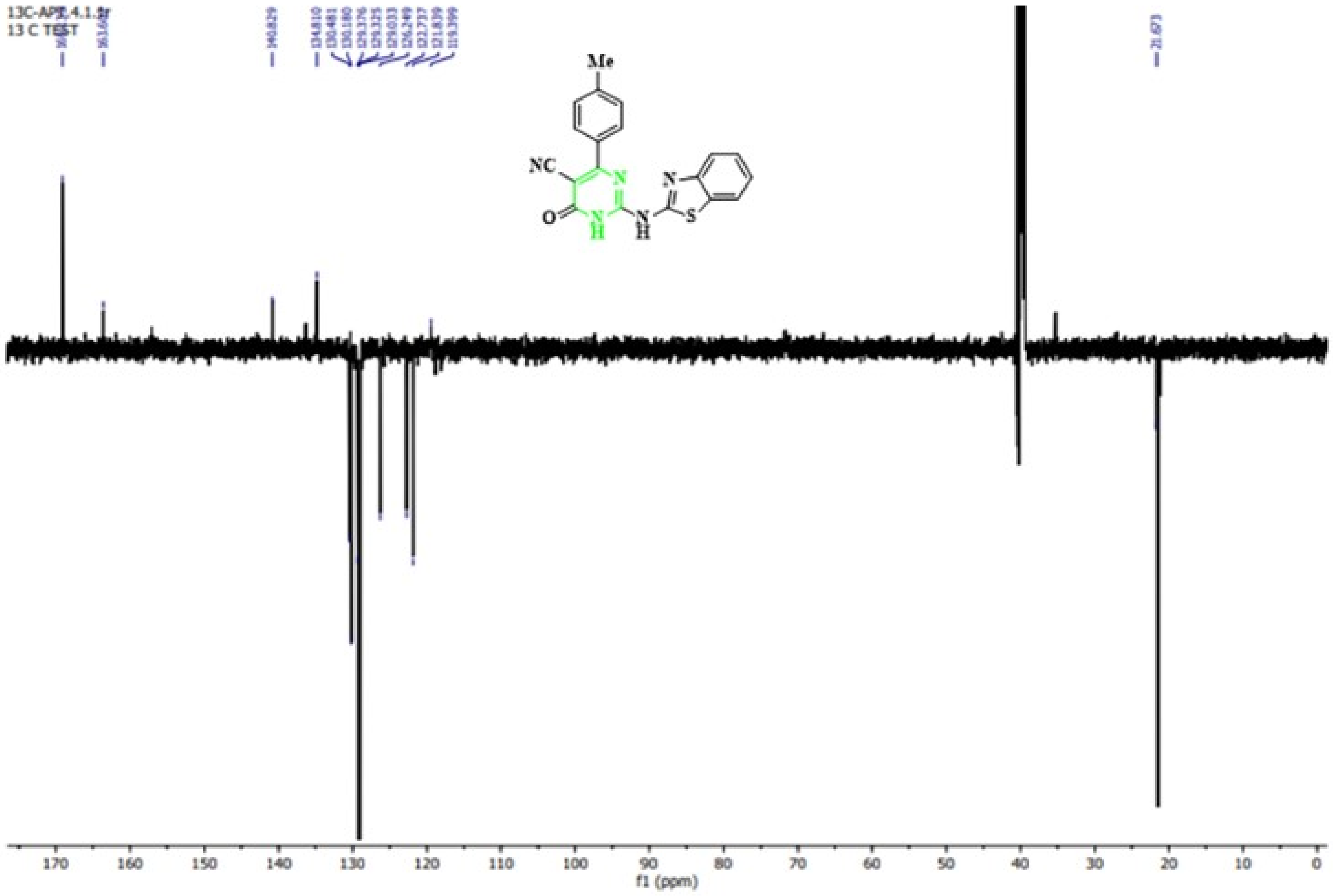

Figure 10. ^{13}C NMR spectrum of compound **7d**

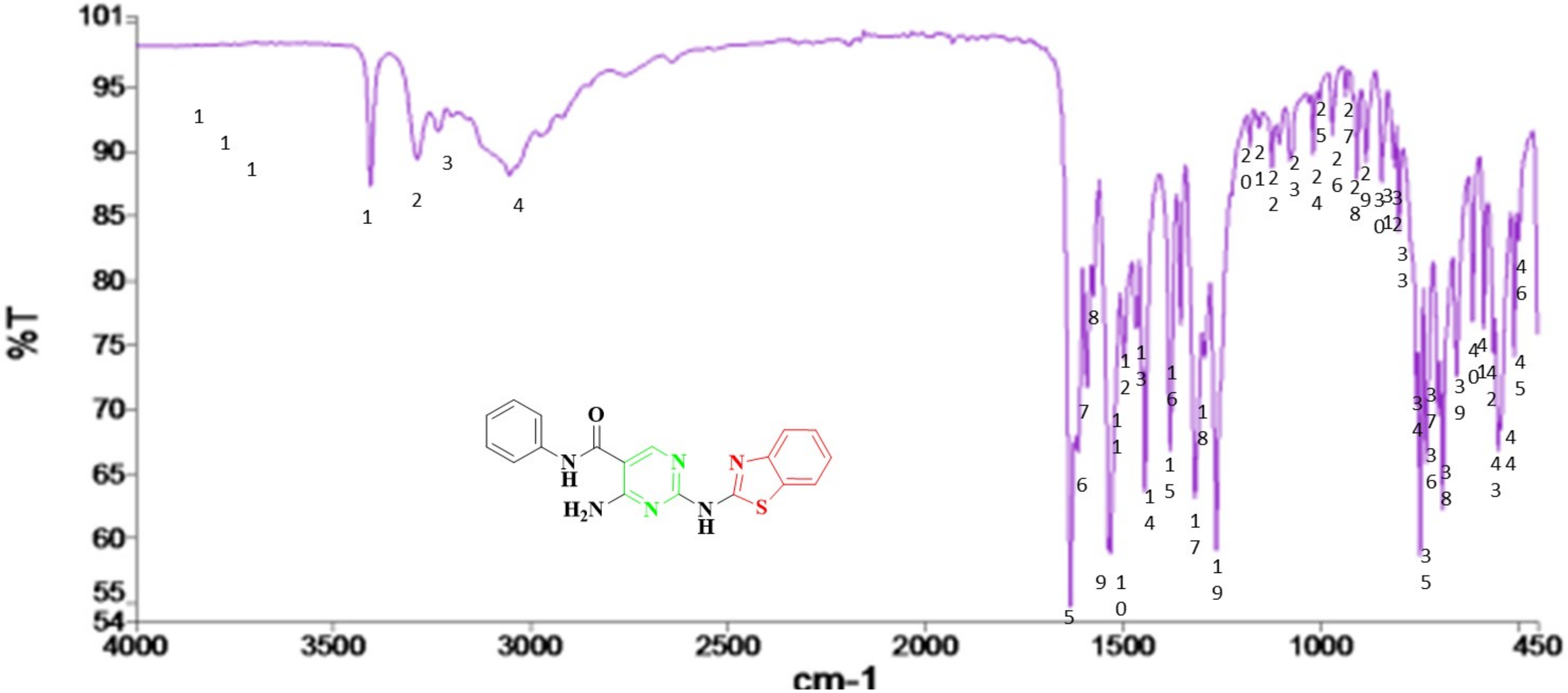

No.	Position	Intinsty	No.	Position	Intinsty	No.	Position	Intinsty	No.	Position	Intinsty	No.	Position	Intinsty
1	3407.64	87.42	11	1497.26	73.86	21	1154.76	91.88	31	845.02	87.71	41	585.22	76.24
2	3289.83	89.50	12	1465.00	76.27	22	1122.90	88.81	32	812.63	88.81	42	560.89	74.36
3	3236.93	91.68	13	1451.96	72.46	23	1104.49	90.67	33	800.89	83.86	43	548.42	66.77
4	3055.95	88.27	14	1444.58	63.58	24	1077.05	89.37	34	757.11	70.40	44	542.03	68.44
5	1633.91	54.63	15	1380.29	66.76	25	1017.87	89.86	35	746.34	58.59	45	508.79	74.16
6	1615.05	66.70	16	1354.20	76.58	26	1001.61	93.99	36	732.39	65.80	46	496.07	83.16
7	1591.20	71.70	17	1317.95	63.07	27	970.03	91.40	37	701.47	69.56			
8	1574.64	78.82	18	1293.26	73.97	28	935.66	94.41	38	689.54	62.22			
9	1537.56	59.11	19	1263.82	59.03	29	907.26	87.93	39	654.72	72.61			
10	1532.42	58.73	20	1178.74	90.41	30	883.73	89.24	40	613.85	76.84			

Figure 11. IR spectrum of compound **13a**

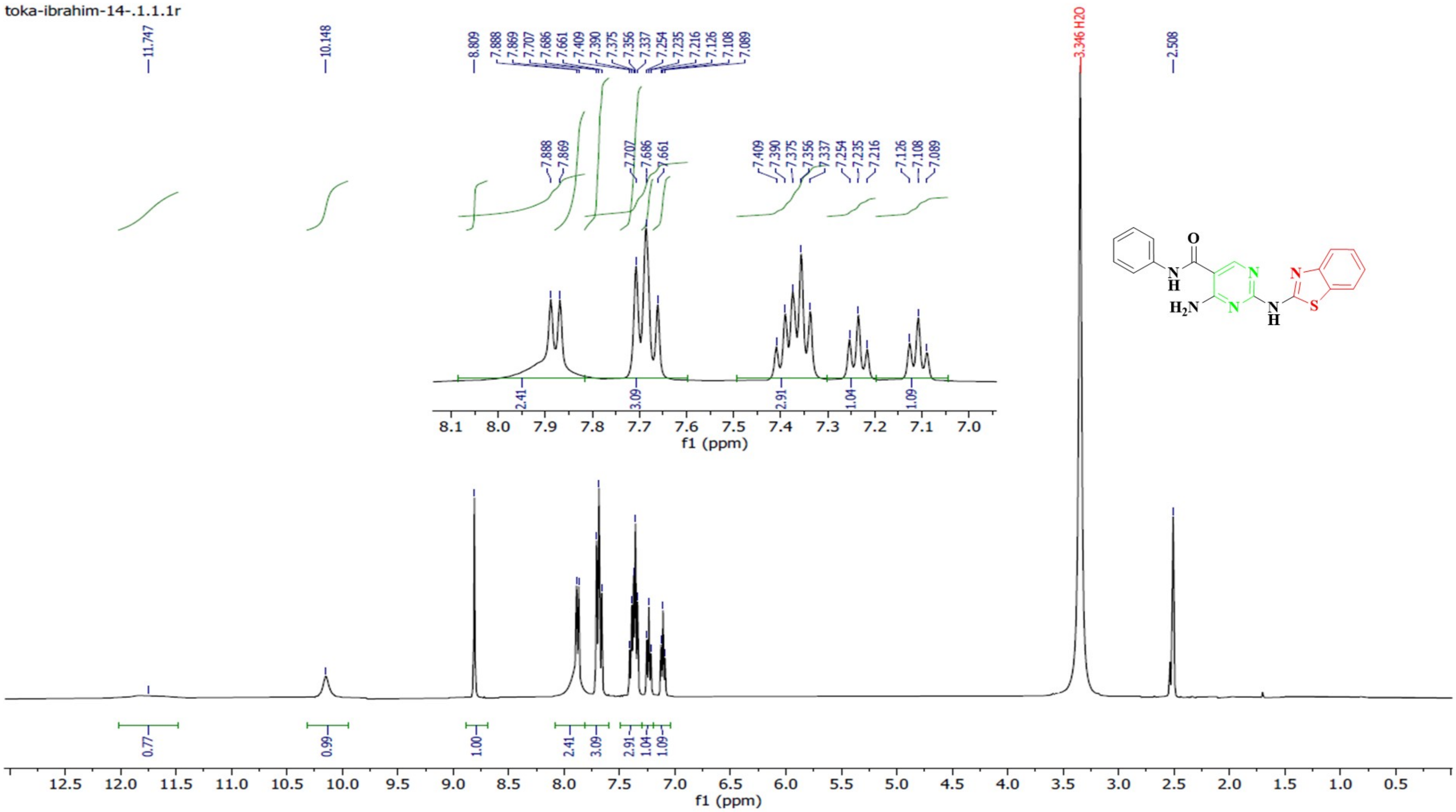

Figure 12. ^{1}H NMR spectrum of compound **13a**

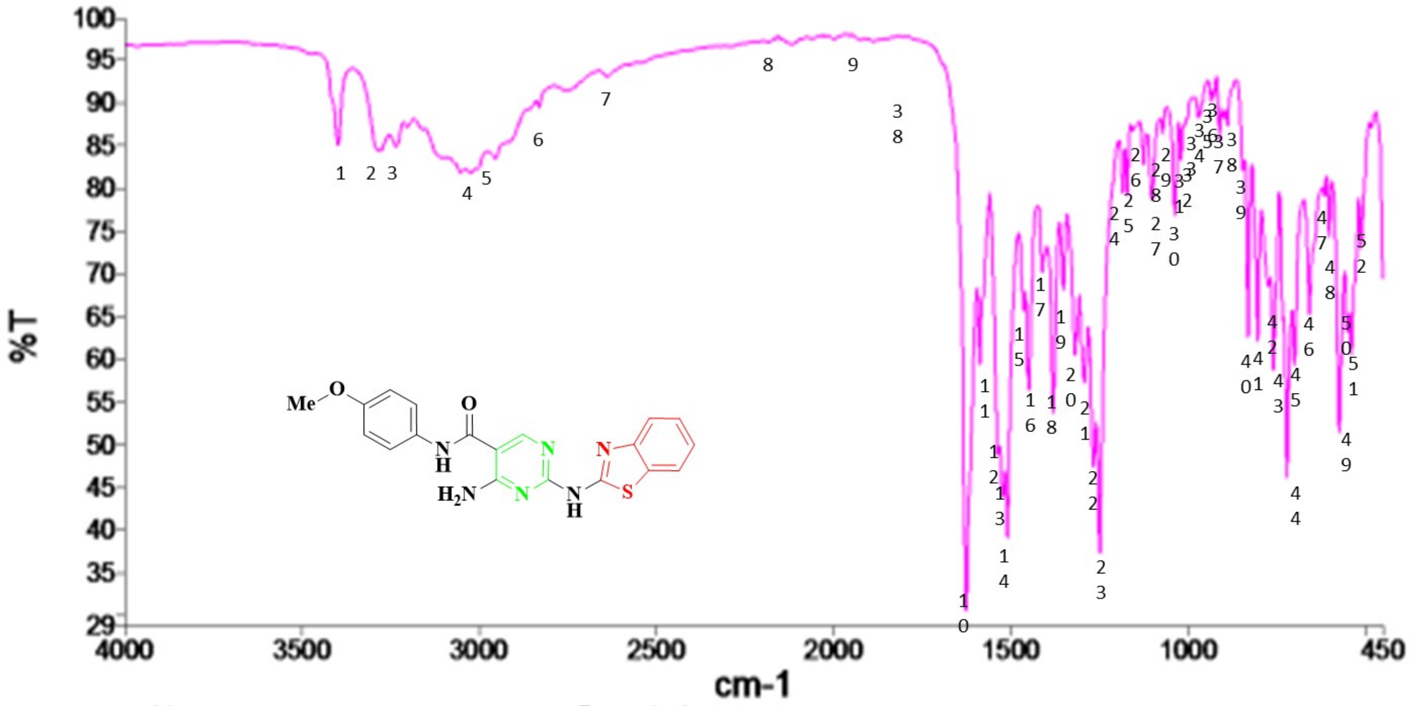

No.	Position	Intinsty	No.	Position	Intinsty	No.	Position	Intinsty	No.	Position	Intinsty	No.	Position	Intinsty
1	3400.92	85.18	11	1588.03	59.44	21	1319.17	60.53	31	1037.27	76.96	41	802.98	62.13
2	3286.67	84.51	12	1537.43	48.83	22	1292.29	57.32	32	1019.63	83.39	42	772.75	68.56
3	3237.36	84.98	13	1523.18	43.84	23	1267.55	47.41	33	971.80	88.37	43	758.15	58.76
4	3027.44	81.85	14	1509.59	39.11	24	1249.18	37.28	34	934.25	90.35	44	719.85	46.17
5	2956.59	83.62	15	1463.80	65.69	25	1184.35	79.51	35	926.48	90.78	45	698.51	59.40
6	2833.06	89.60	16	1453.94	58.17	26	1171.05	79.36	36	910.91	85.79	46	657.47	65.31
7	2640.45	93.15	17	1448.16	56.44	27	1155.71	86.82	37	900.58	88.40	47	614.42	79.22
8	2117.04	96.98	18	1411.67	70.32	28	1125.89	82.85	38	888.72	87.48	48	600.79	74.38
9	1891.21	97.27	19	1380.27	53.71	29	1101.07	78.80	39	845.17	82.22	49	572.88	51.46
10	1626.60	30.49	20	1351.40	68.08	30	1071.20	86.49	40	830.15	62.66	50	549.22	63.19
51	538.20	60.57	52	510.46	73.05									

Figure 13. IR spectrum of compound **13b**

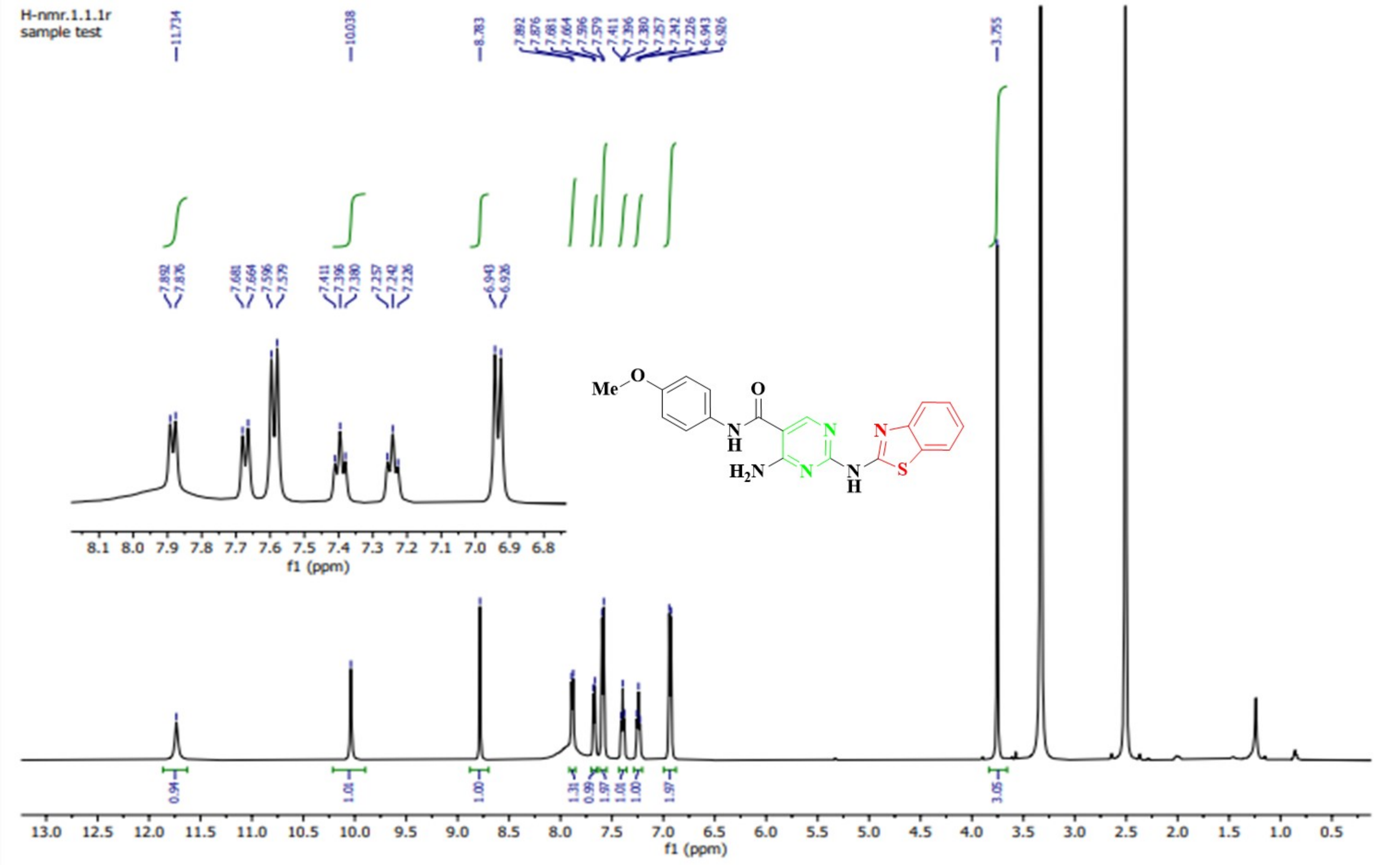

Figure 14. ^{1}H NMR spectrum of compound **13b**

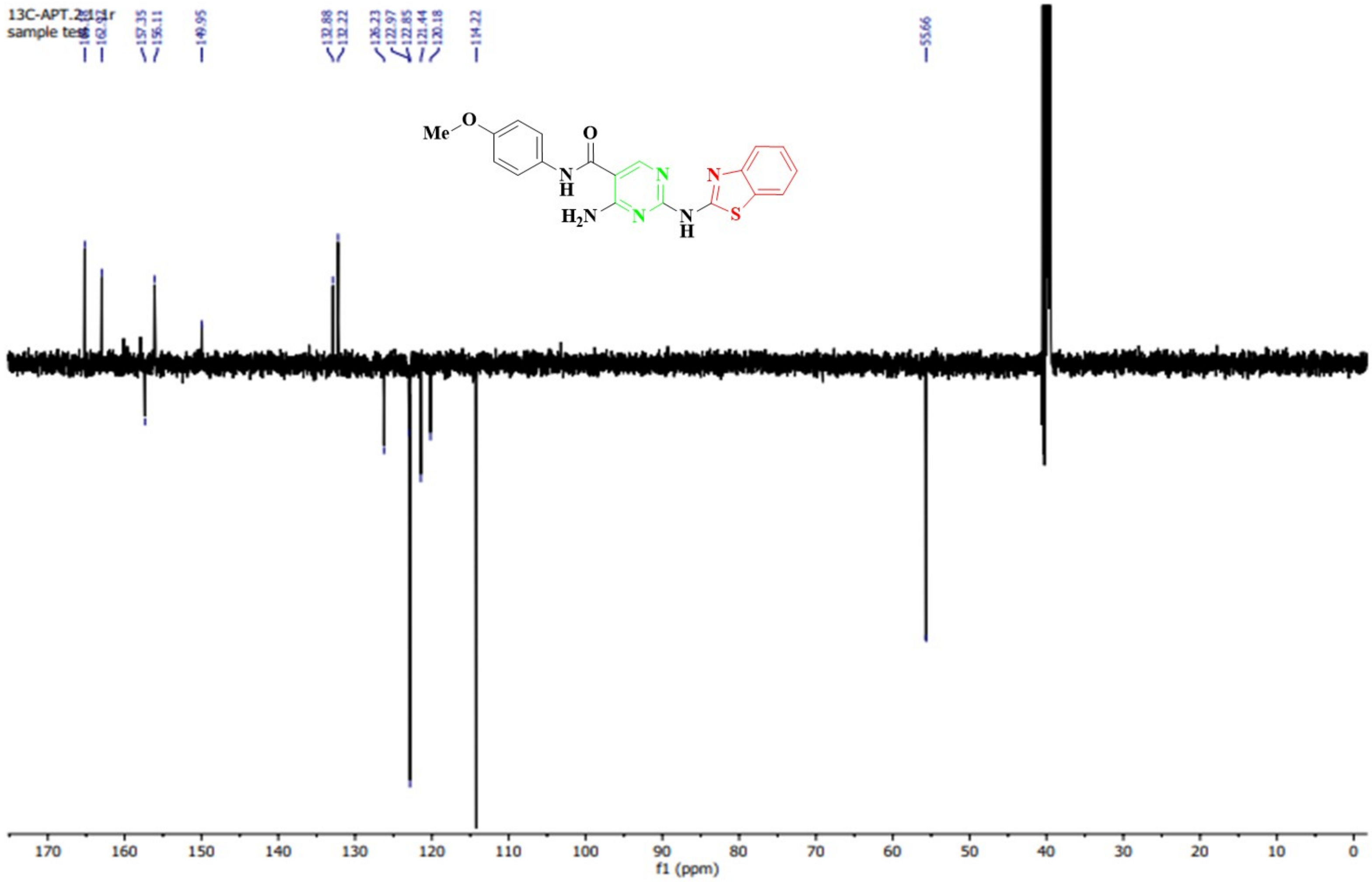

Figure 15. ^{13}C NMR spectrum of compound **13b**

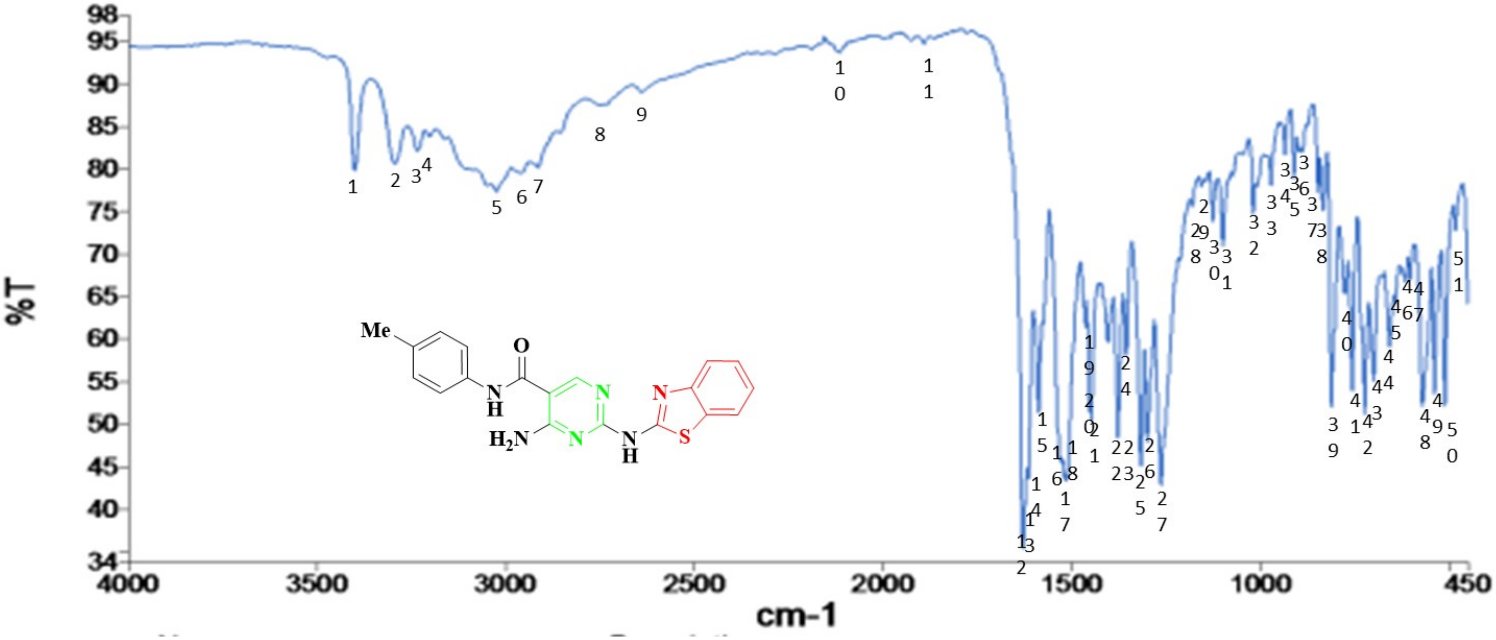

No.	Position	Intinsty	No.	Position	Intinsty	No.	Position	Intinsty	No.	Position	Intinsty	No.	Position	Intinsty
1	3402.92	79.94	12	1628.66	35.29	22	1403.98	59.64	32	1018.62	74.79	42	722.67	51.14
2	3296.91	80.72	13	1615.67	43.53	23	1377.88	48.47	33	971.80	78.16	43	698.23	55.07
3	3236.26	82.21	14	1588.28	51.35	24	1355.01	58.14	34	933.76	81.71	44	657.60	59.14
4	3204.93	83.92	15	1531.26	45.66	25	1316.67	45.12	35	910.39	78.94	45	640.86	64.23
5	3027.50	77.44	16	1525.05	45.26	26	1299.26	47.90	36	888.22	82.10	46	614.77	66.78
6	2960.29	79.61	17	1519.77	43.51	27	1262.74	42.76	37	846.63	77.37	47	601.39	67.07
7	2916.62	80.21	18	1515.13	43.28	28	1180.64	75.66	38	832.80	75.17	48	569.78	52.11
8	2751.37	87.65	19	1463.32	61.34	29	1156.21	78.06	39	810.85	51.99	49	537.33	53.49
9	2641.11	89.17	20	1453.62	51.51	30	1126.23	73.85	40	775.69	65.34	50	510.16	52.17
10	2116.55	93.86	21	1447.57	50.42	31	1099.45	71.03	41	755.61	53.90	51	481.04	72.85
11	1892.06	94.88												

Figure 16. IR spectrum of compound **13c**

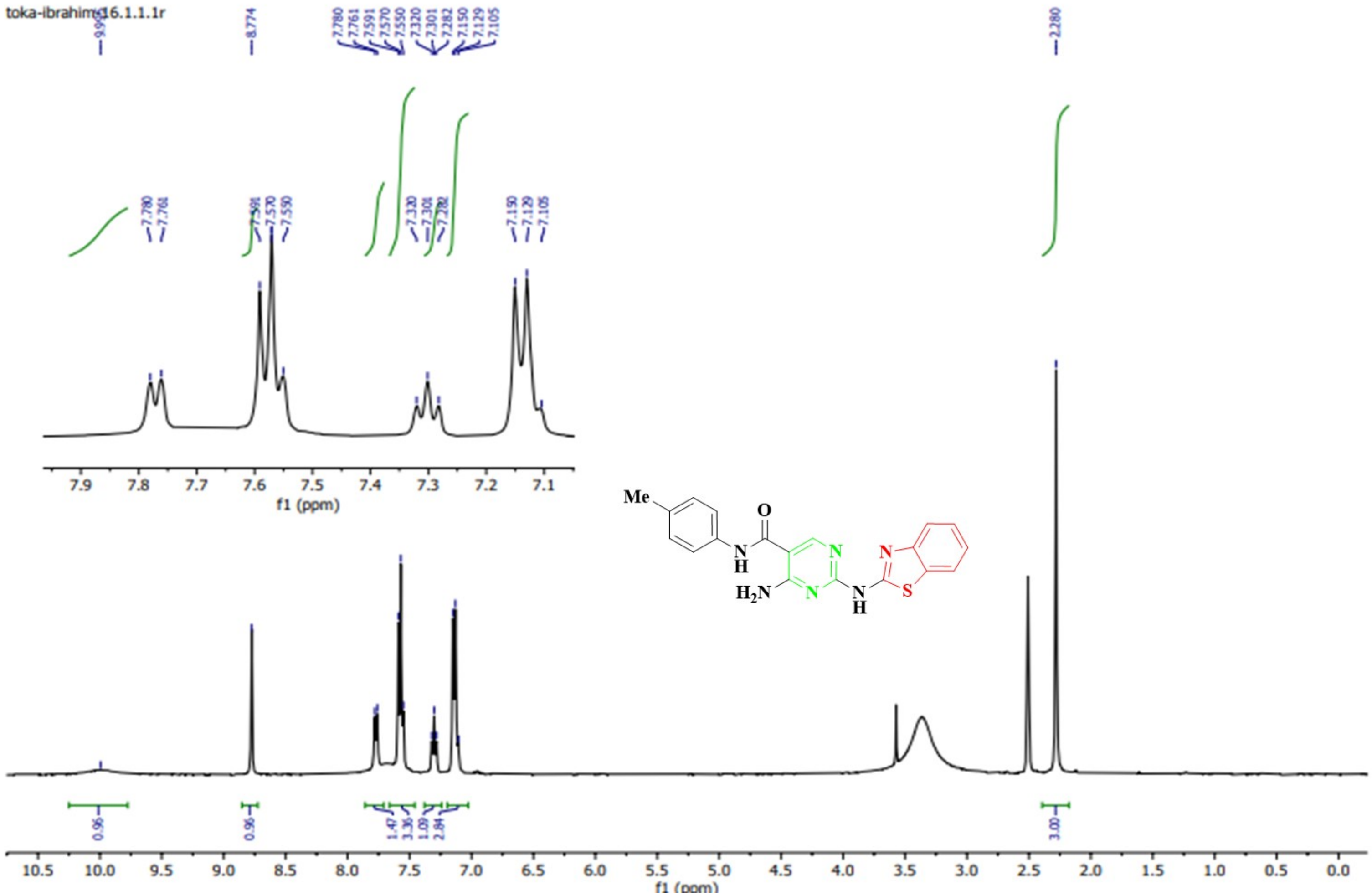

Figure 17. ^{1}H NMR spectrum of compound **13c**

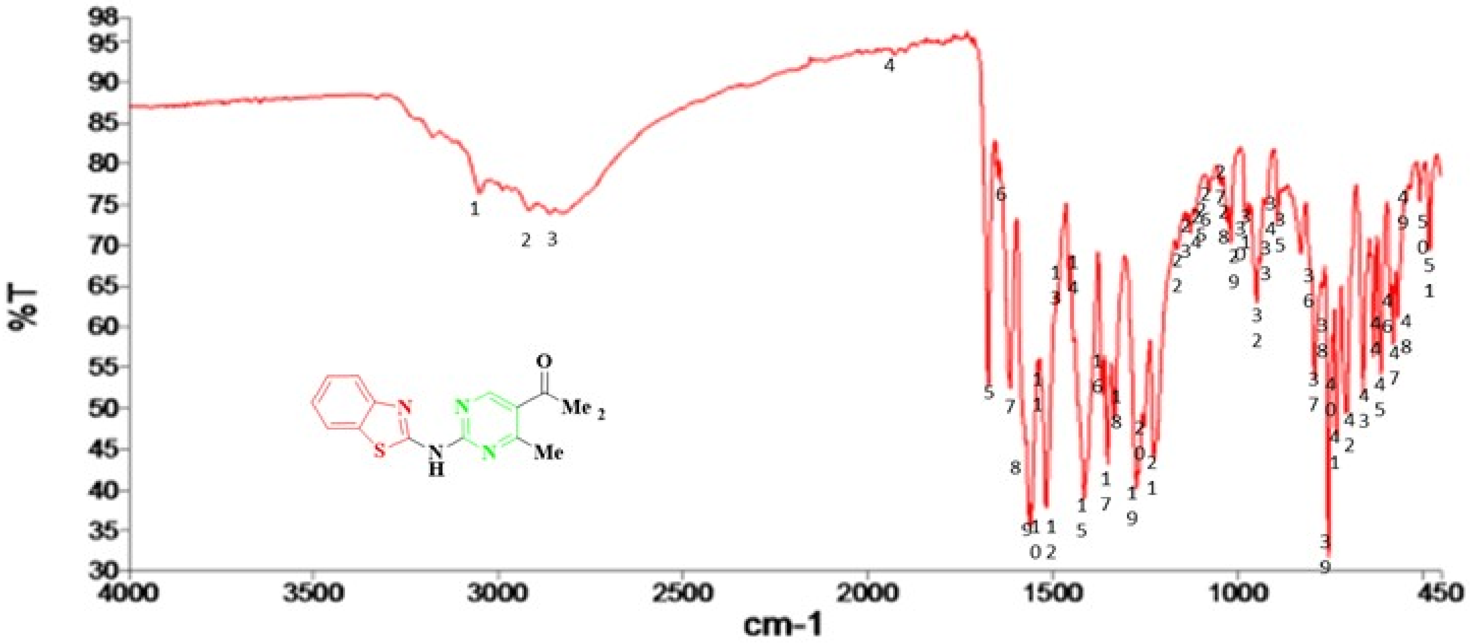

No.	Position	Intinsty	No.	Position	Intinsty	No.	Position	Intinsty	No.	Position	Intinsty	No.	Position	Intinsty
1	3052.59	76.42	12	1518.71	37.79	22	1162.86	69.59	32	947.45	62.97	42	704.73	49.32
2	2917.76	74.35	13	1486.75	62.86	23	1137.29	73.15	33	938.45	67.80	43	658.79	51.60
3	2862.04	73.96	14	1456.01	64.51	24	1127.77	71.52	34	921.51	75.00	44	630.14	56.42
4	1928.75	93.48	15	1416.17	38.69	25	1114.82	73.71	35	891.45	72.83	45	611.52	54.12
5	1674.41	52.73	16	1366.82	51.5	26	1076.43	76.68	36	828.30	69.00	46	585.95	62.63
6	1650.37	78.49	17	1352.32	43.11	27	1046.18	77.44	37	790.57	53.44	47	577.82	57.86
7	1615.48	52.49	18	1333.49	48.61	28	1028.74	72.87	38	769.00	64.89	48	564.83	61.12
8	1574.19	45.55	19	1274.51	40.07	29	1017.55	70.36	39	753.74	31.69	49	531.88	76.98
9	1563.53	35.37	20	1252.42	47.70	30	978.36	73.05	40	742.02	58.21	50	506.24	75.48
10	1557.84	35.67	21	1226.41	43.93	31	966.01	73.89	41	731.07	46.48	51	479.90	69.31
11	1538.57	54.75												

Figure 18. IR spectrum of compound **15a**

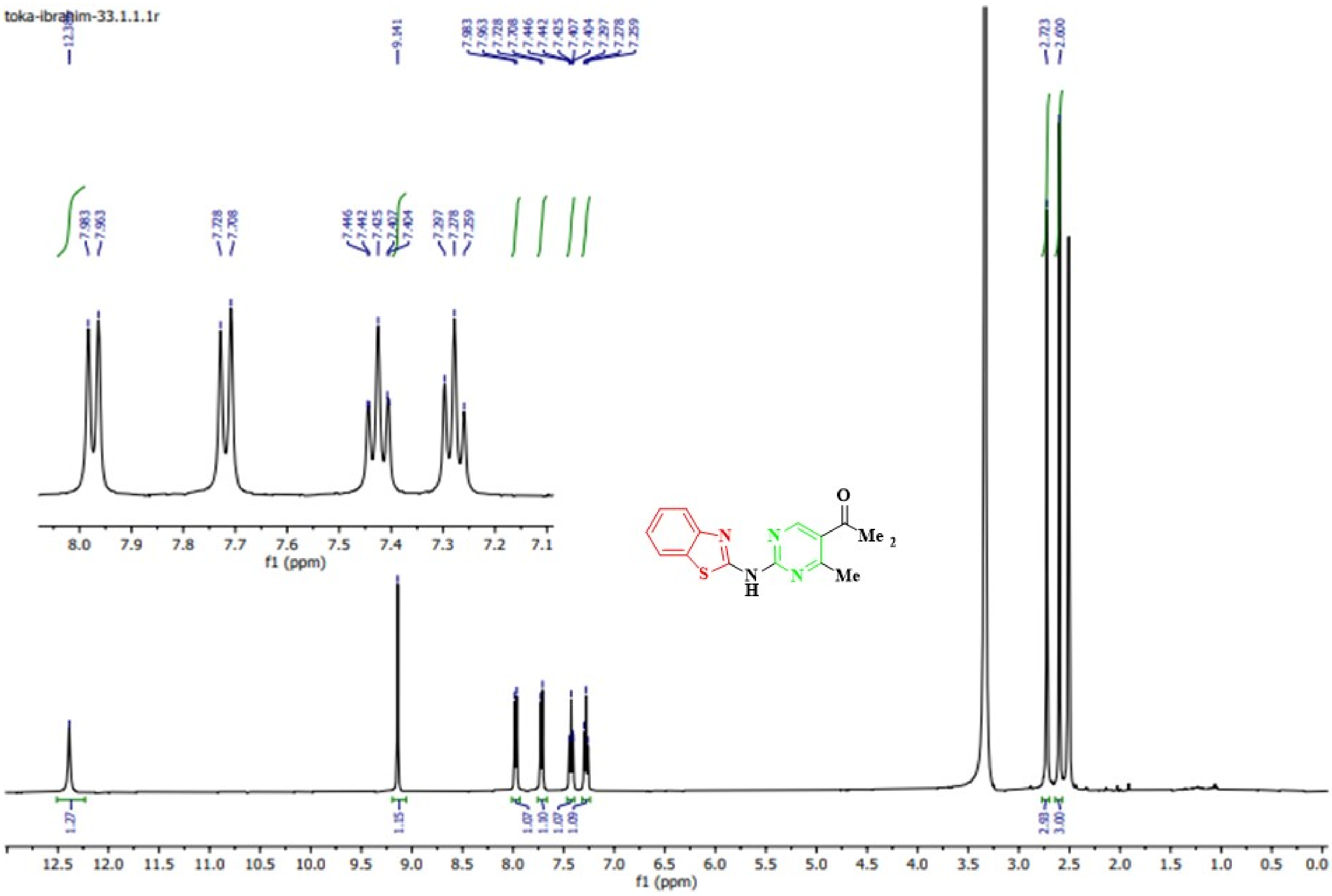

Figure 19. ^{1}H NMR spectrum of compound **15a**

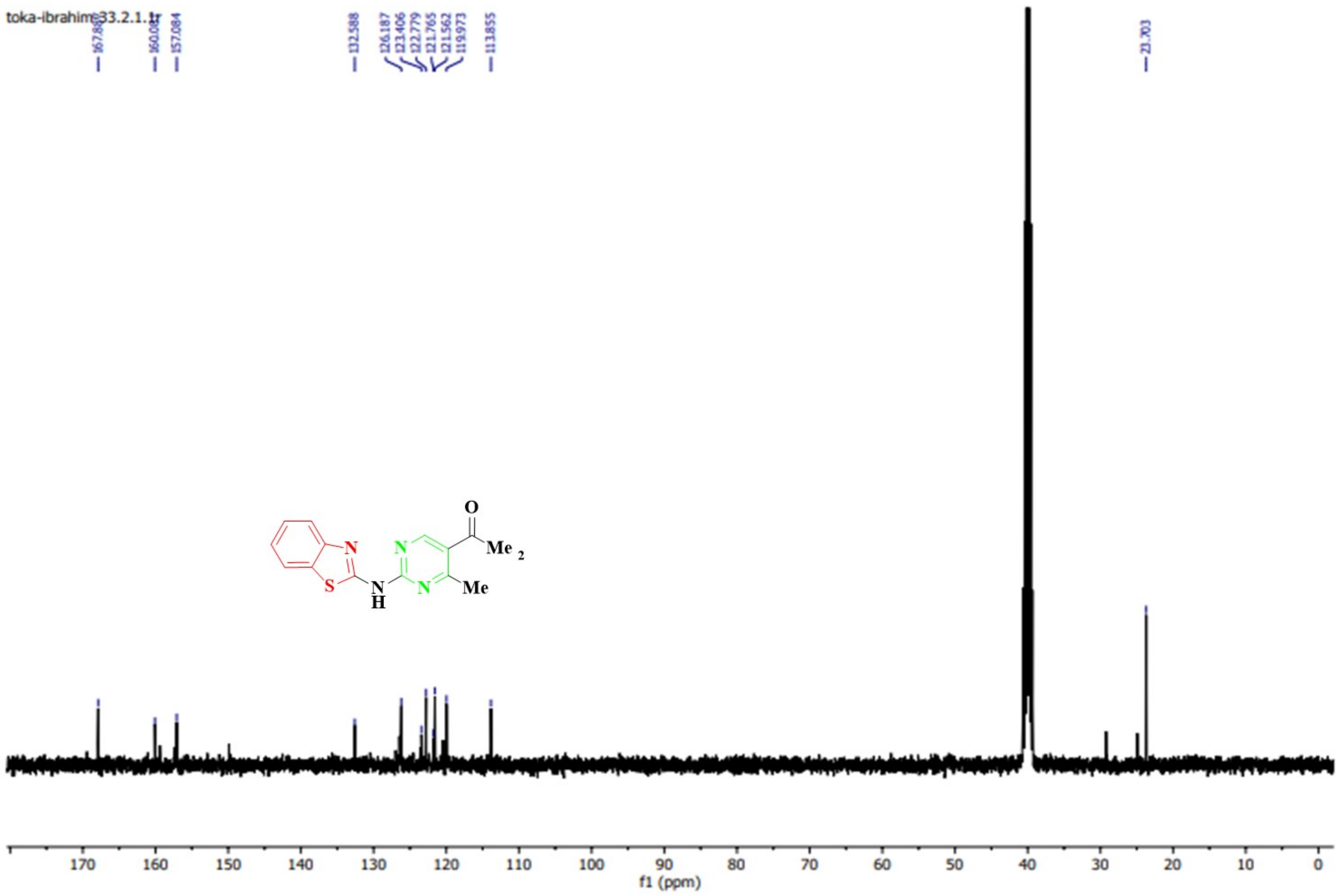

Figure 20. ^{13}C NMR spectrum of compound **15a**

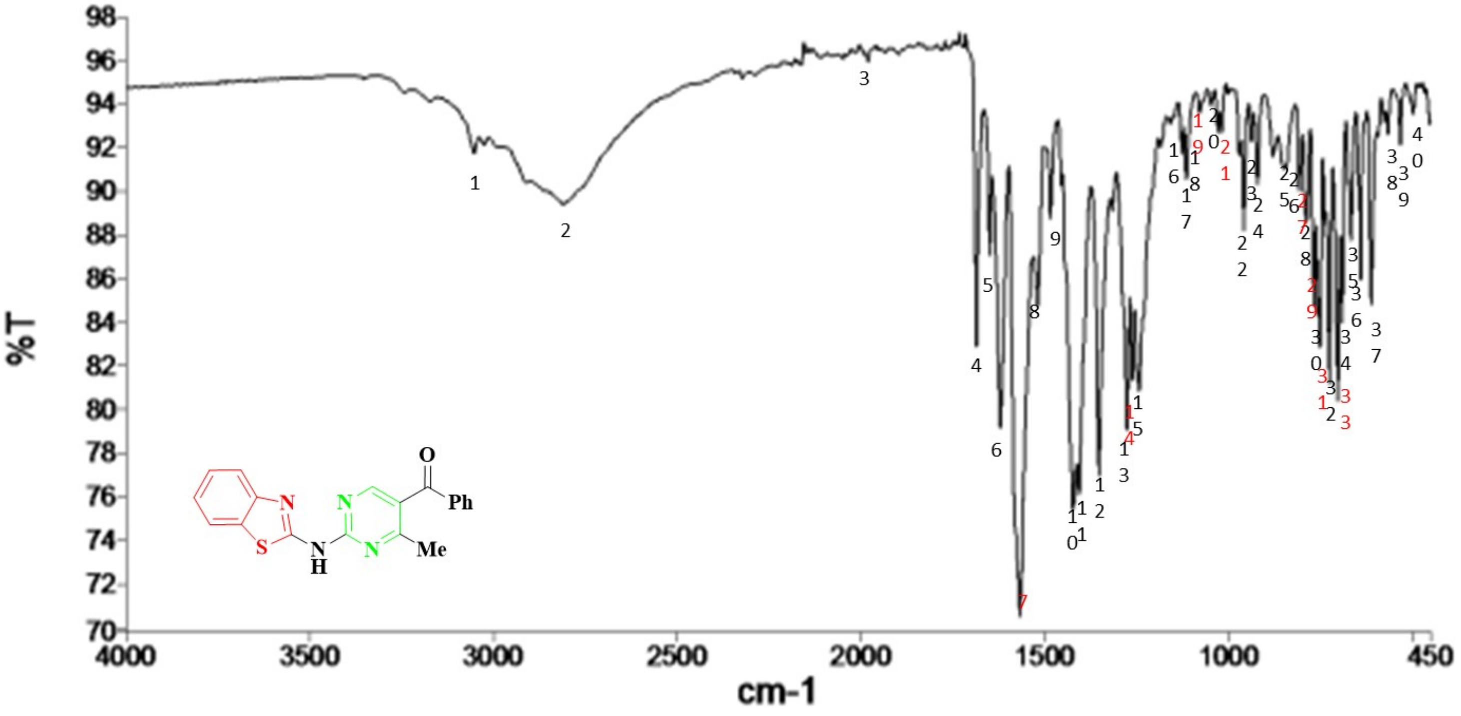

No.	Position	Intinsty	No.	Position	Intinsty	No.	Position	Intinsty	No.	Position	Intinsty	No.	Position	Intinsty
1	3054.78	91.79	9	1484.15	88.76	17	1114.30	90.60	25	878.17	91.62	33	701.52	80.40
2	2811.04	89.44	10	1422.82	75.41	18	1077.68	93.64	26	847.00	91.06	34	689.70	84.00
3	1980.67	95.99	11	1406.97	76.10	19	1046.83	93.96	27	807.01	90.07	35	665.52	87.77
4	1686.17	82.91	12	1352.42	76.98	20	1028.13	92.75	28	787.92	88.58	36	638.50	85.97
5	1650.57	87.08	13	1275.43	79.05	21	1018.14	92.75	29	766.69	84.7	37	610.61	84.84
6	1620.92	79.16	14	1261.25	81.37	22	957.39	88.23	30	755.47	84.28	38	564.68	92.65
7	1567.70	70.48	15	1242.57	80.85	23	937.47	92.35	31	749.44	82.87	39	531.04	92.19
8	1519.09	84.77	16	1126.59	91.75	24	920.01	90.36	32	723.80	81.19	40	496.08	93.60

Figure 21. IR spectrum of compound **15b**

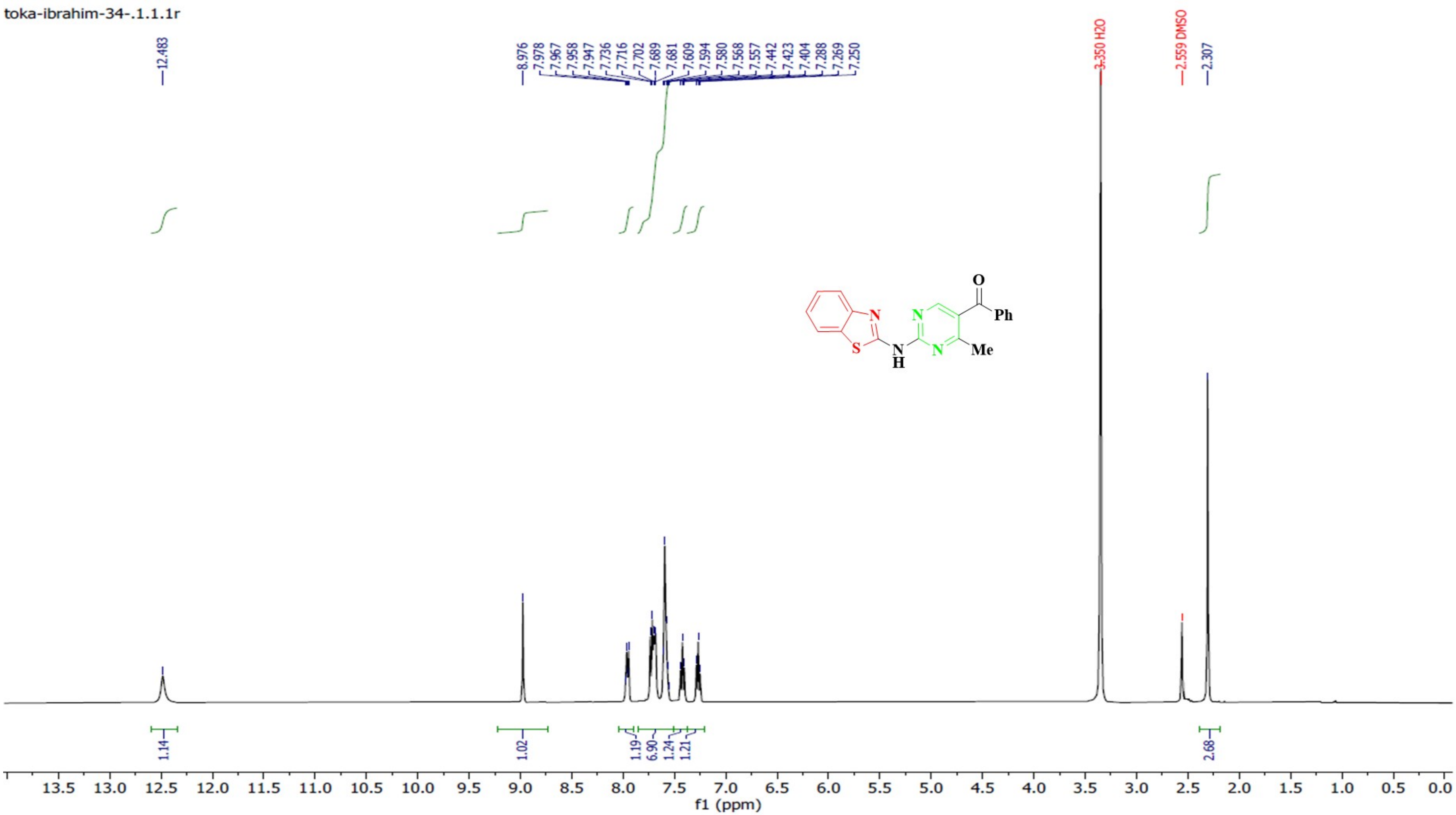

Figure 22. ^{1}H NMR spectrum of compound **15b**

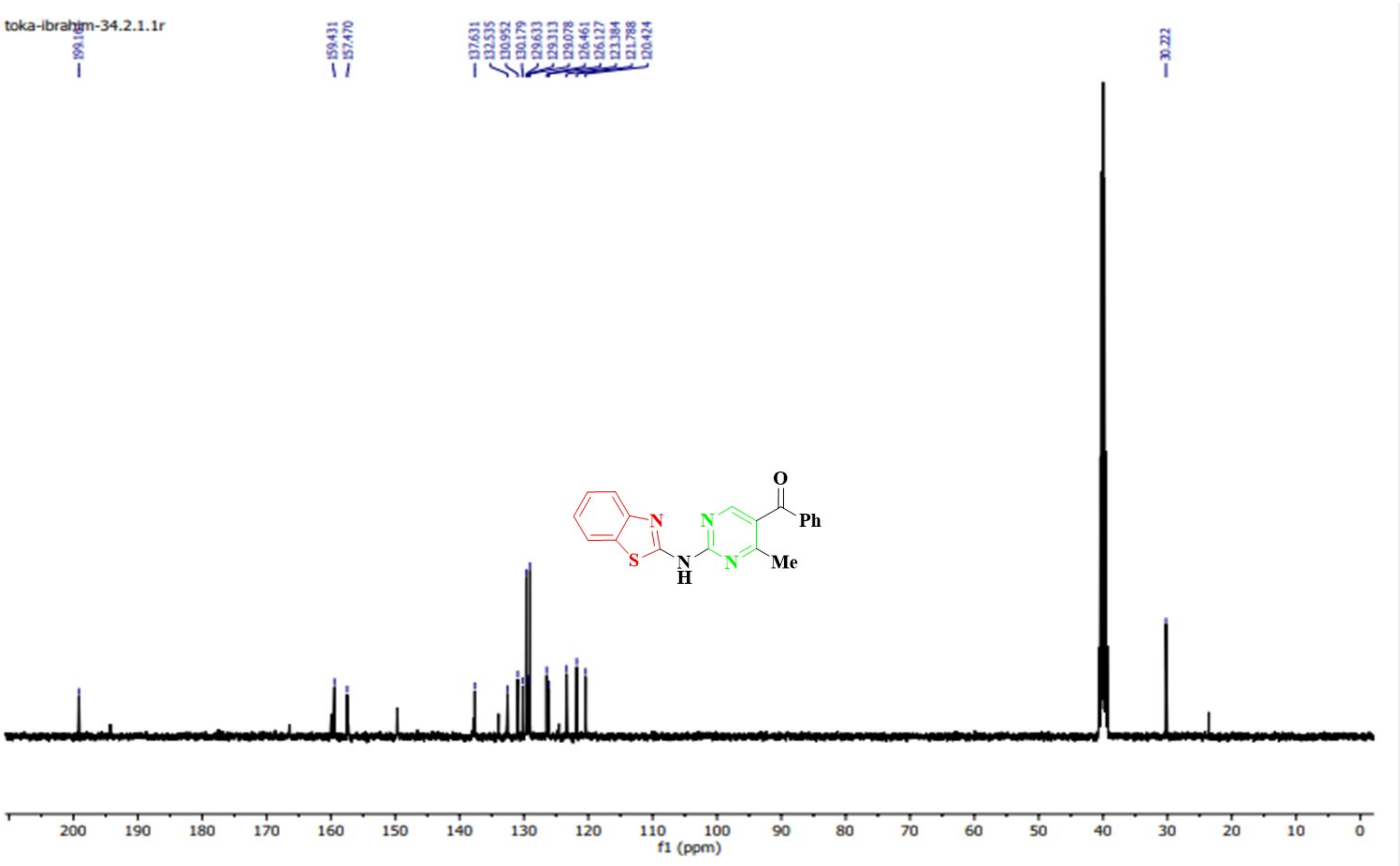

Figure 23. ^{13}C NMR spectrum of compound **15b**

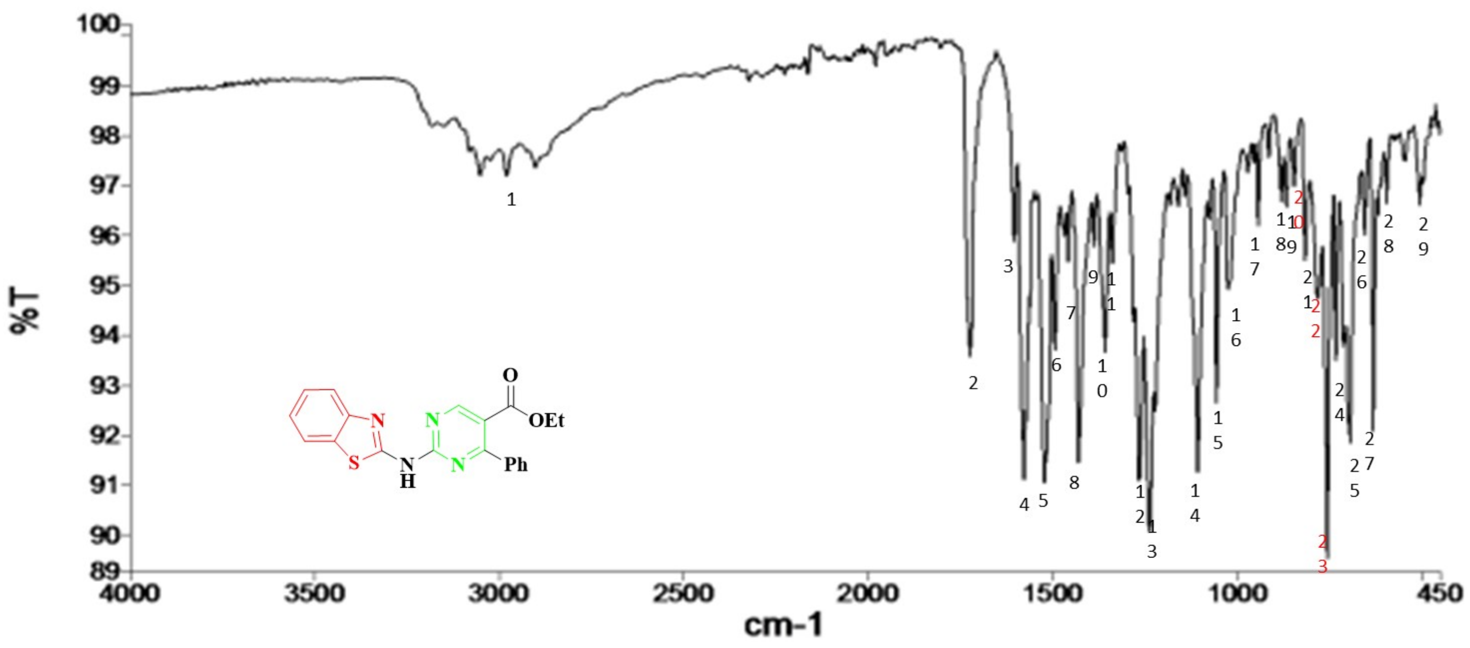

No.	Position	Intinsty	No.	Position	Intinsty	No.	Position	Intinsty	No.	Position	Intinsty	No.	Position	Intinsty
1	2982.06	97.22	7	1458.11	95.49	13	1237.36	90.05	19	865.63	96.58	25	692.72	91.84
2	1724 .28	93.58	8	1430.07	91.45	14	1106.45	91.26	20	845.10	97.00	26	653.52	96.02
3	1604.07	95.90	9	1388.35	95.79	15	1055.40	92.66	21	815.11	95.51	27	630.36	92.08
4	1578.00	91.12	10	1357.88	93.66	16	1024.89	94.93	22	781.69	94.74	28	593.60	96.66
5	1522.63	91.04	11	1338.64	95.45	17	942.42	96.21	23	755.24	89.53	29	504.23	96.63
6	1493.47	93.71	12	1265.57	91.07	18	880.38	96.70	24	731.96	93.50			

Figure 24. IR spectrum of compound **15c**

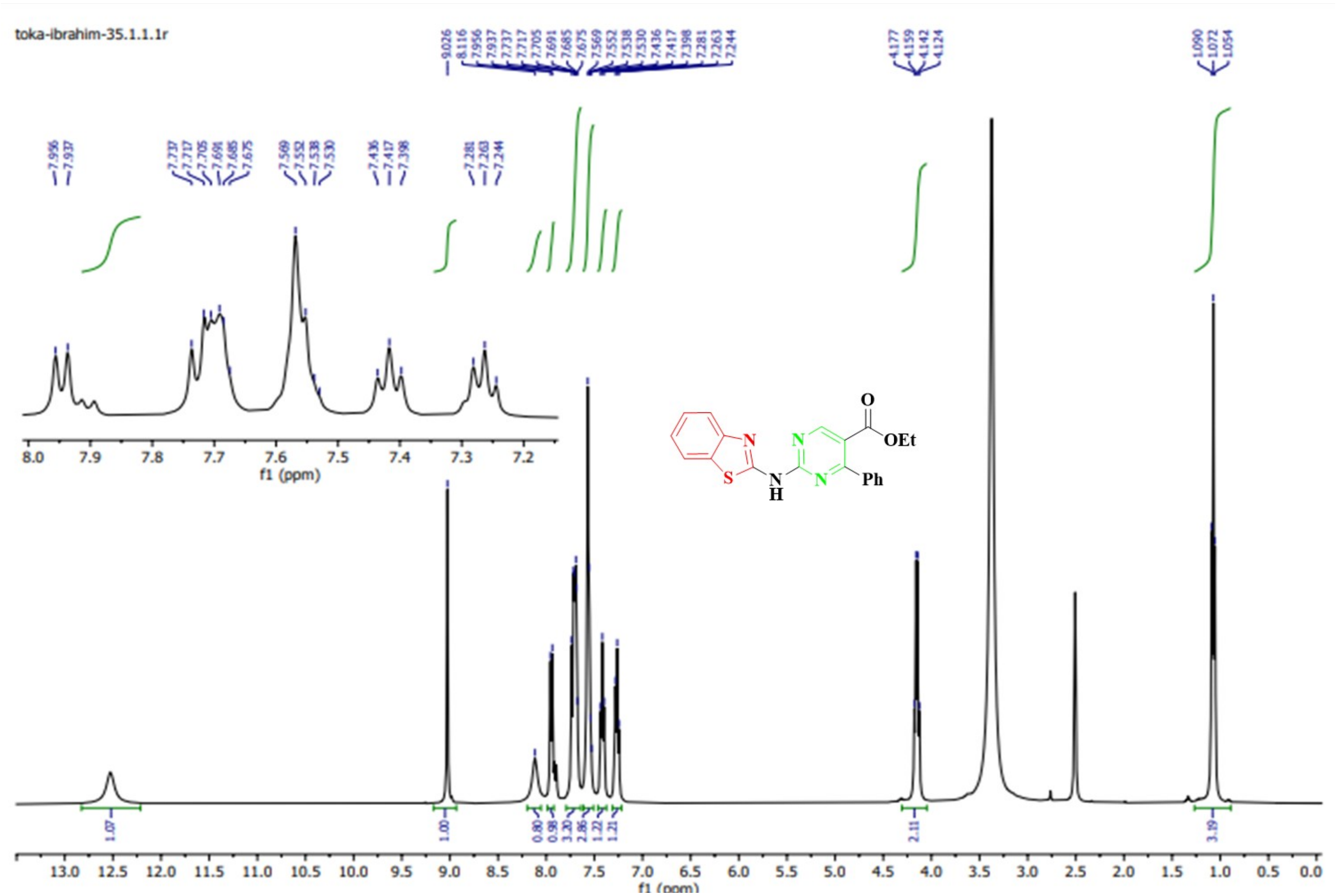

Figure 25. ^{1}H NMR spectrum of compound **15c**

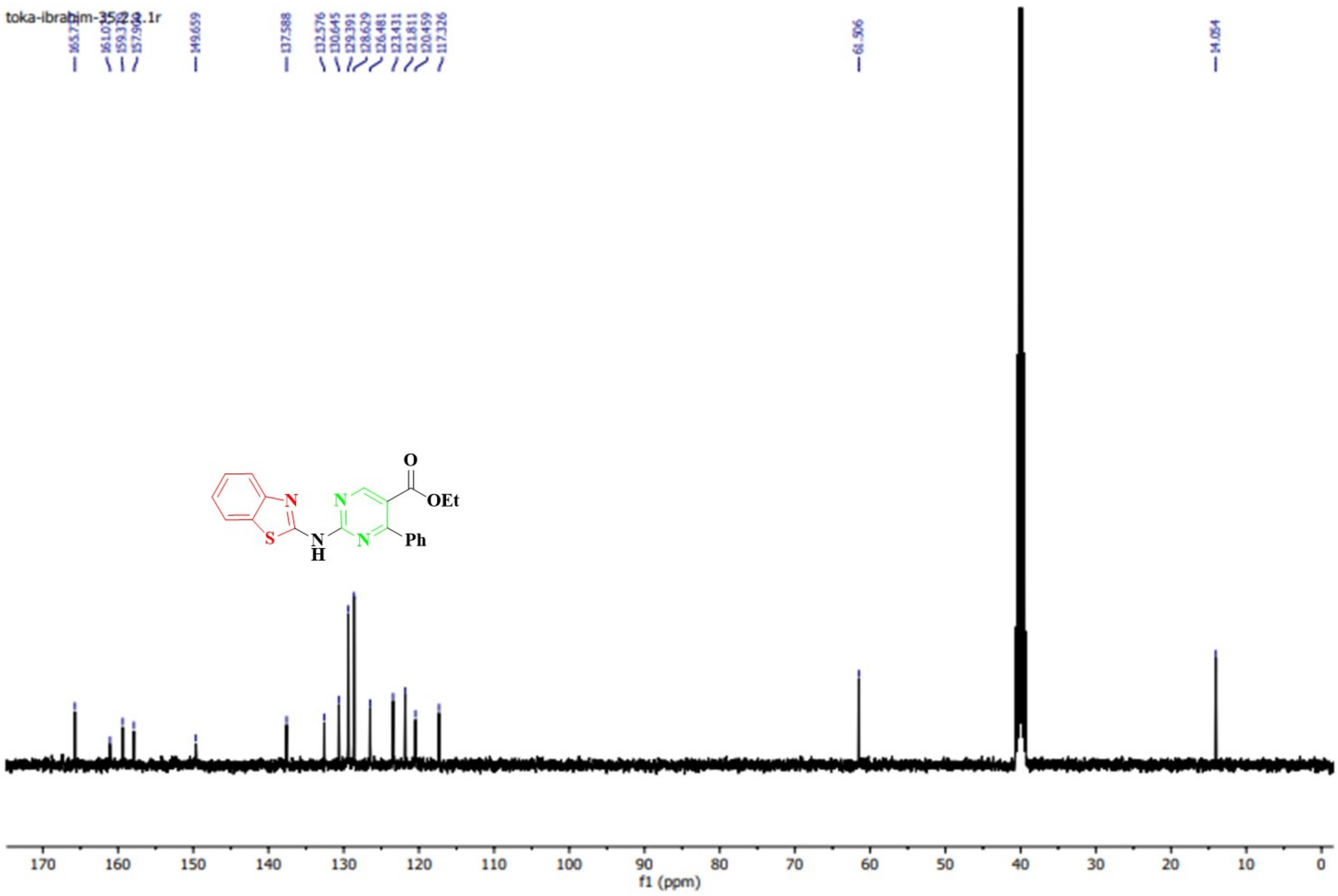

Figure 26. ^{13}C NMR spectrum of compound **15c**

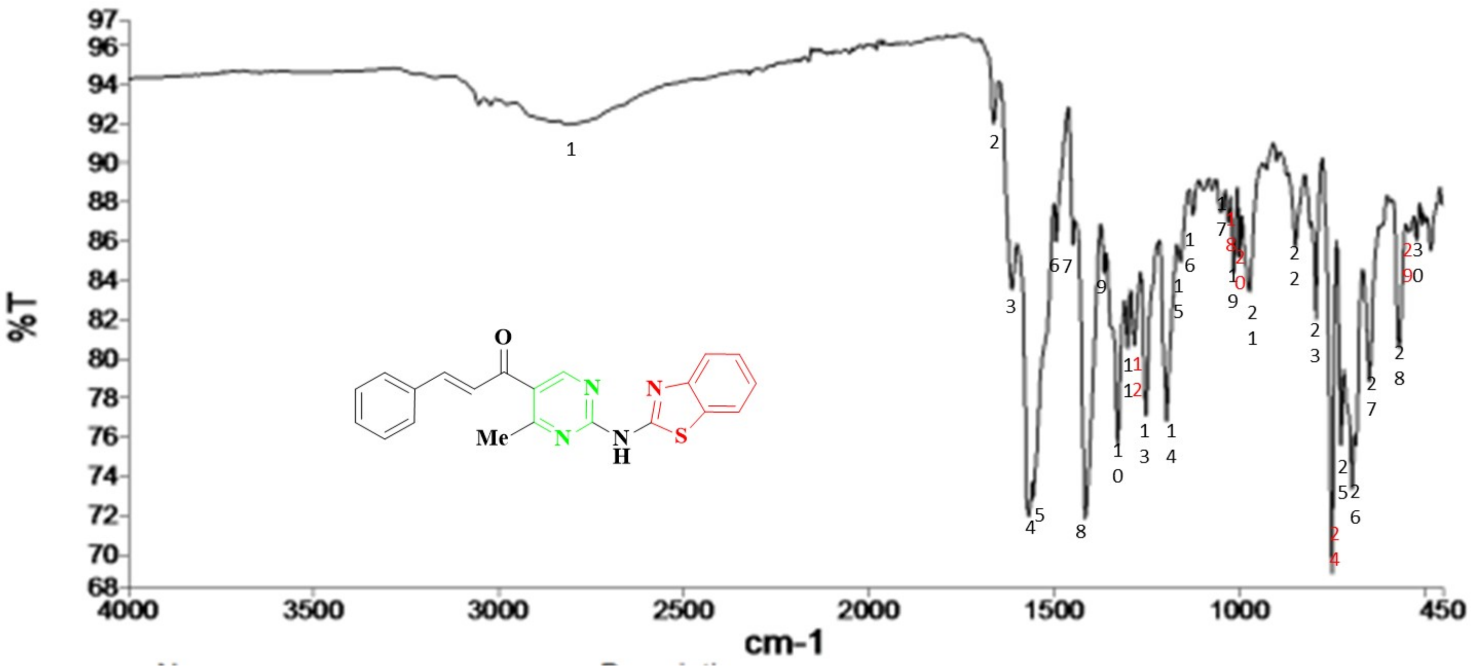

No.	Position	Intensity	No.	Position	Intensity	No.	Position	Intensity	No.	Position	Intensity	No.	Position	Intensity
1	2815.22	92.01	7	1448.55	85.81	13	1251.91	77.12	19	1014.00	84.4	25	724.48	75.57
2	1663.16	92.02	8	1415.78	71.80	14	1195.96	76.81	20	998.79	85.19	26	694.12	73.30
3	1614.67	83.53	9	1364.22	84.37	15	1158.13	84.94	21	972.34	83.44	27	647.81	78.86
4	1568.04	71.92	10	1327.77	75.59	16	1124.63	87.34	22	847.79	85.73	28	567.88	80.54
5	1557.63	72.83	11	1301.31	80.52	17	1048.97	87.49	23	791.87	82.03	29	519.54	86.06
6	1494.42	86.01	12	1282.87	80.69	18	1029.50	86.99	24	748.68	69.00	30	481.66	85.54

Figure 27. IR spectrum of compound **17a**

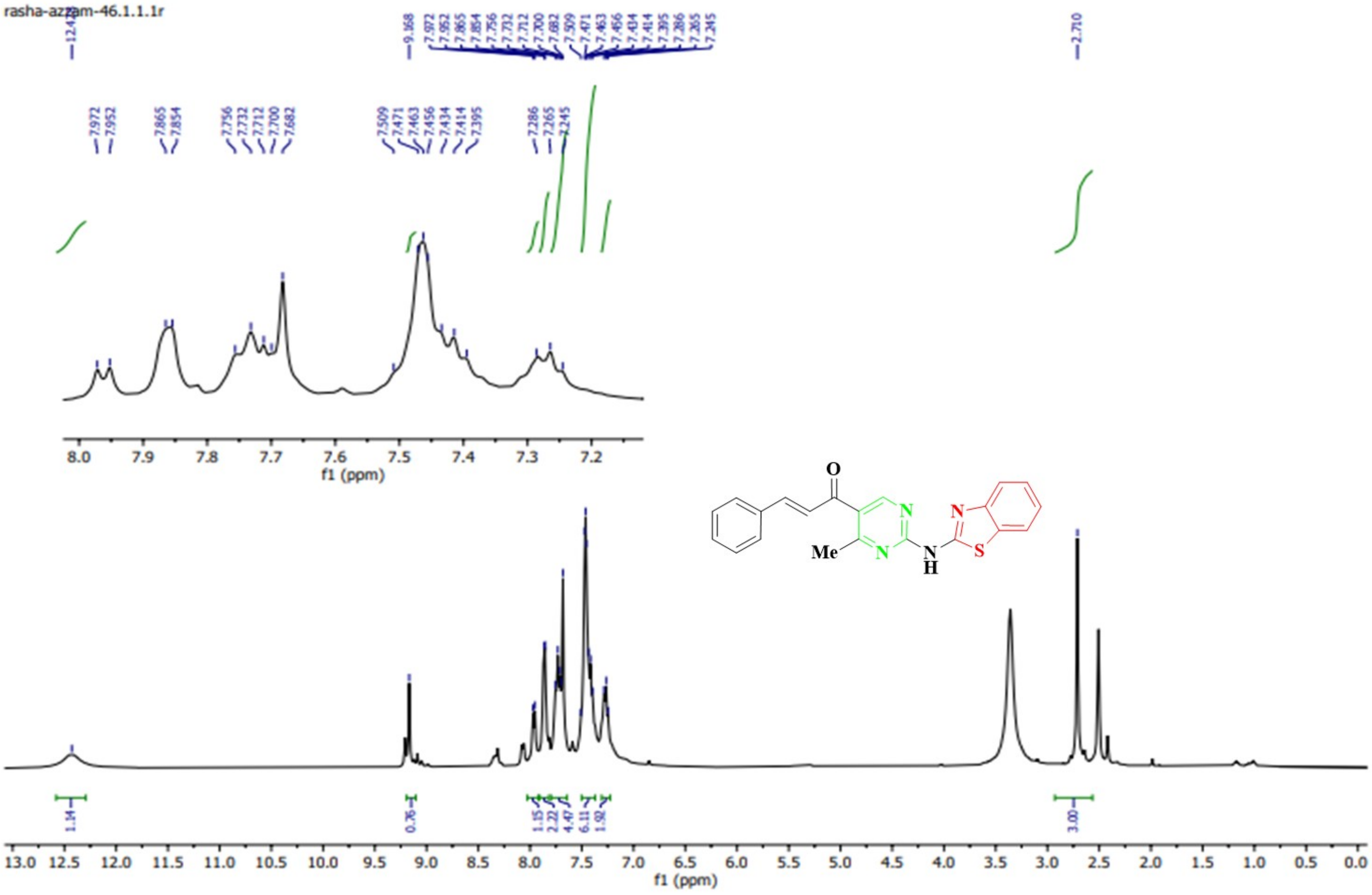

Figure 28. ^{1}H NMR spectrum of compound **17a**

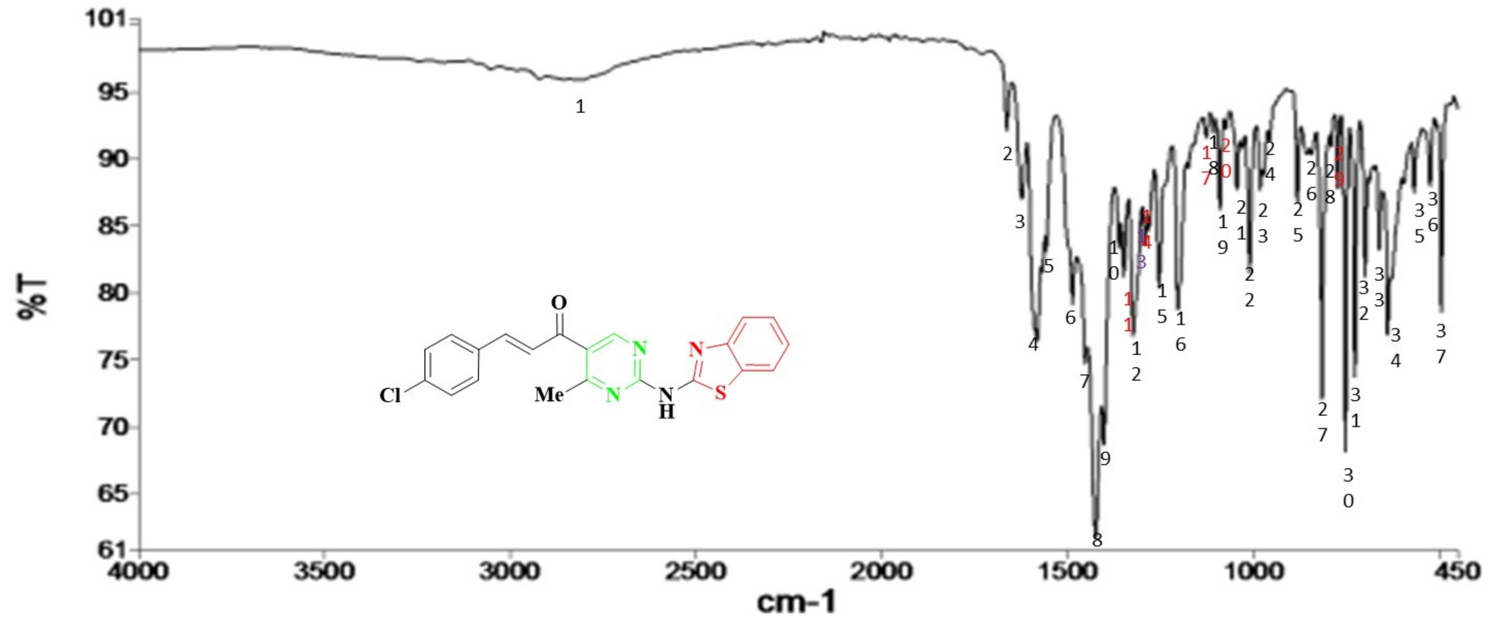

No.	Position	Intinsty	No.	Position	Intinsty	No.	Position	Intinsty	No.	Position	Intinsty	No.	Position	Intinsty
1	2826.67	95.96	9	1402.05	68.66	17	1126.94	91.63	25	880.53	86.78	33	660.10	83.23
2	1663.83	92.17	10	1360.87	83.34	18	1107.45	92.03	26	843.20	90.33	34	637.69	76.86
3	1623.04	87.02	11	1348.09	81.27	19	1089.80	86.25	27	814.64	72.08	35	566.31	87.54
4	1582.01	76.40	12	1322.96	76.78	20	1073.43	92.33	28	789.30	90.97	36	523.01	88.02
5	1557.63	83.12	13	1291.24	83.57	21	1043.57	87.76	29	771.90	87.81	37	492.87	78.62
6	1487.39	79.19	14	1280.86	84.64	22	1009.27	81.55	30	751.29	68.09			
7	1452.36	74.69	15	1253.42	80.41	23	980.96	87.71	31	725.94	73.75			
8	1424.44	61.74	16	1203.05	78.75	24	956.21	91.40	32	698.27	81.26			

Figure 29. IR spectrum of compound **17b**

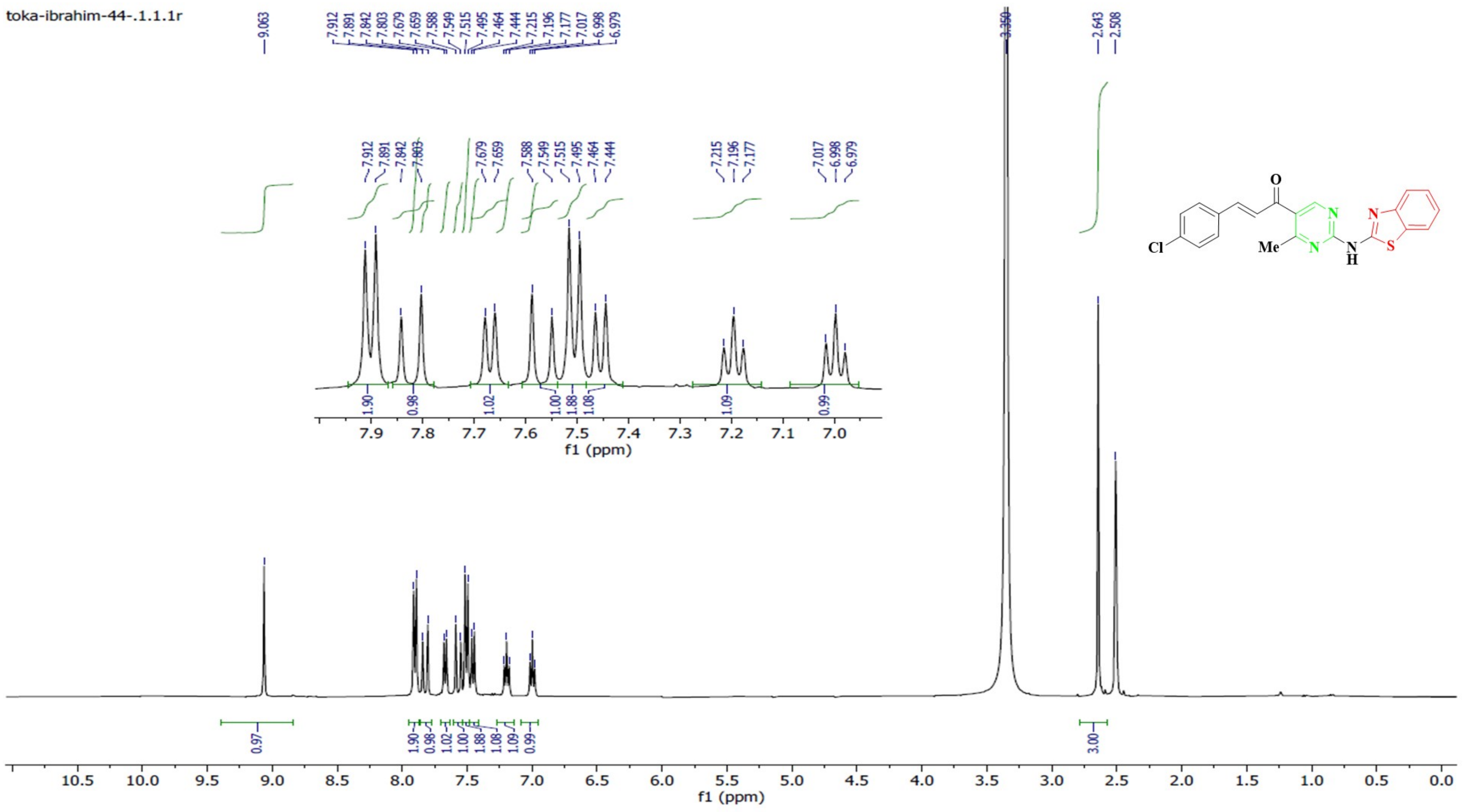

Figure 30. ^{1}H NMR spectrum of compound **17b**

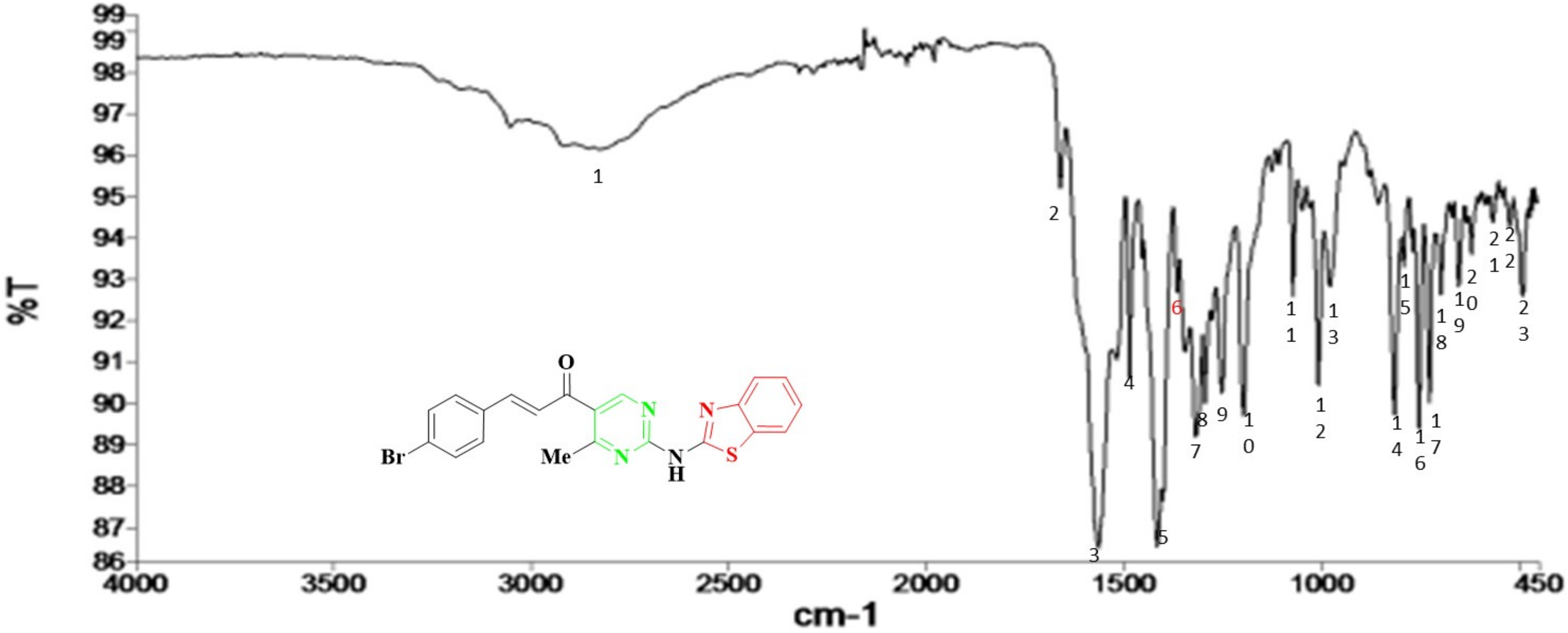

No.	Position	Intinsty	No.	Position	Intinsty	No.	Position	Intinsty	No.	Position	Intinsty	No.	Position	Intinsty
1	2825.11	96.16	6	1365.56	92.68	11	1071.41	92.60	16	752.79	89.37	21	565.62	94.38
2	1661.31	95.21	7	1318.46	89.19	12	1007.53	90.43	17	725.91	90.02	22	522.86	94.25
3	1563.61	86.48	8	1294.59	90.00	13	978.51	92.83	18	696.85	92.64	23	489.21	92.59
4	1486.12	90.61	9	1252.14	90.24	14	814.07	89.71	19	651.68	92.82			
5	1416.25	86.50	10	1196.33	89.70	15	791.22	93.30	20	619.33	93.61			

Figure 31. IR spectrum of compound **17c**

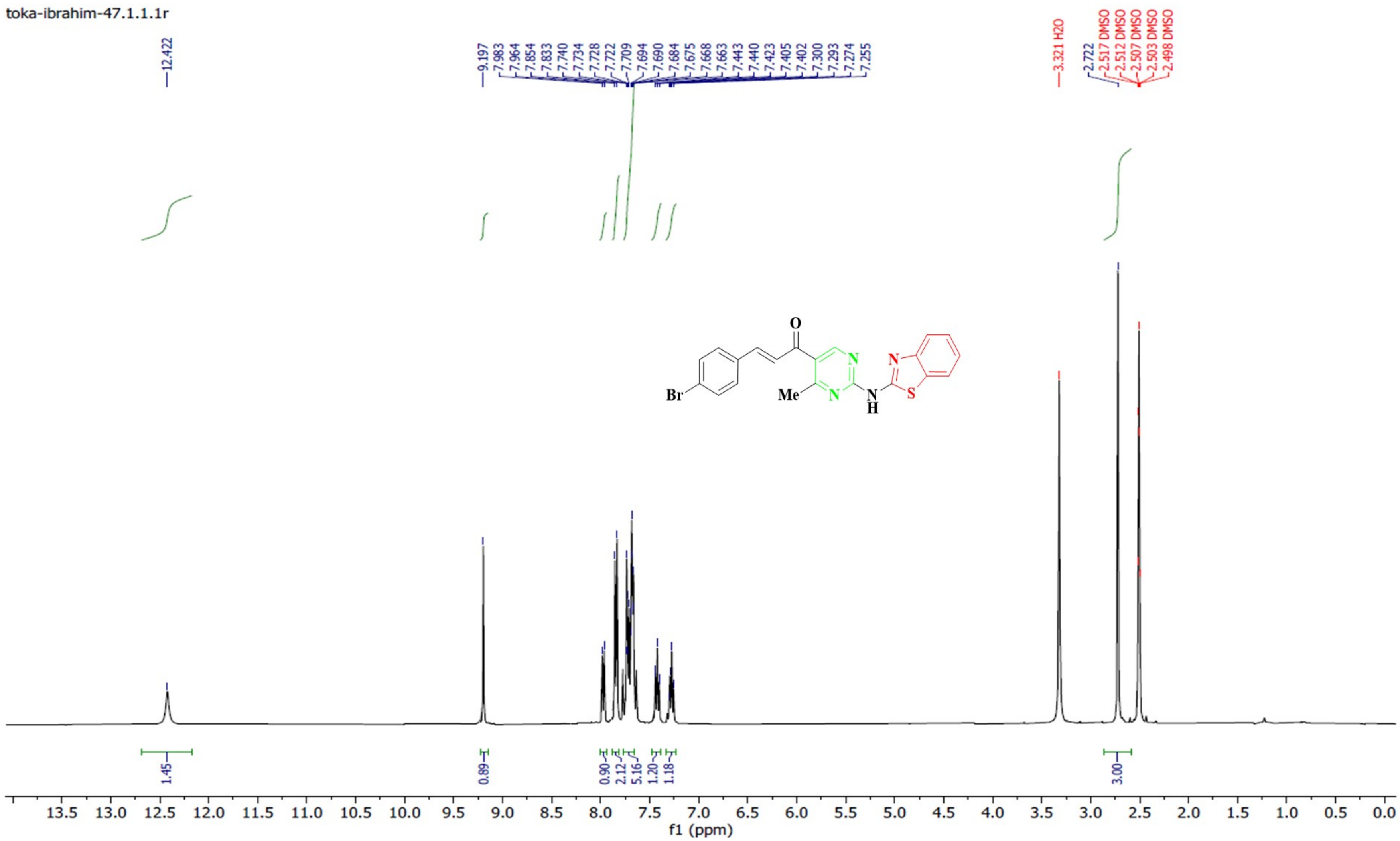

Figure 32. ^{1}H NMR spectrum of compound **17c**

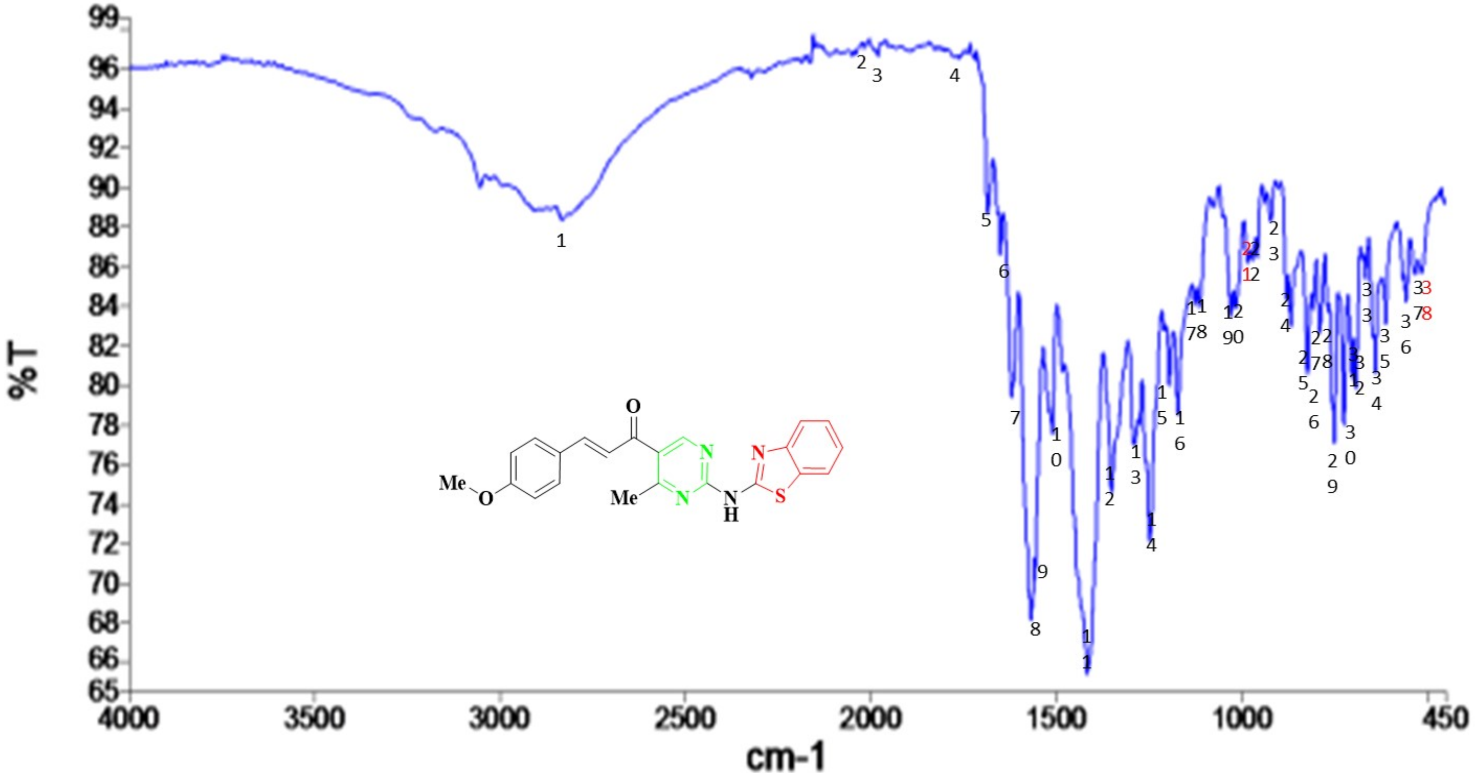

No.	Position	Intinsty	No.	Position	Intinsty	No.	Position	Intinsty	No.	Position	Intinsty	No.	Position	Intinsty
1	2834.31	88.38	9	1558.00	70.13	17	1126.94	84.08	25	866.68	83.05	33	666.12	85.40
2	2050.95	96.71	10	1511.04	77.51	18	1113.82	83.97	26	822.06	80.58	34	638.76	80.63
3	1981.14	96.71	11	1416.71	65.32	19	1028.84	83.53	27	807.72	83.83	35	611.26	83.10
4	1763.01	96.61	12	1351.30	74.57	20	1015.73	83.93	28	788.98	82.68	36	555.72	84.23
5	1685.40	88.71	13	1290.08	77.00	21	982.77	86.23	29	749.35	77.09	37	531.39	85.62
6	1651.68	86.67	14	1247.76	72.08	22	958.06	86.47	30	723.62	77.99	38	514.18	85.68
7	1620.97	79.39	15	1195.12	79.99	23	921.34	88.30	31	701.32	80.31			
8	1568.03	68.09	16	1171.82	78.52	24	879.64	84.35	32	690.49	79.87			

Figure 33. IR spectrum of compound **17d**

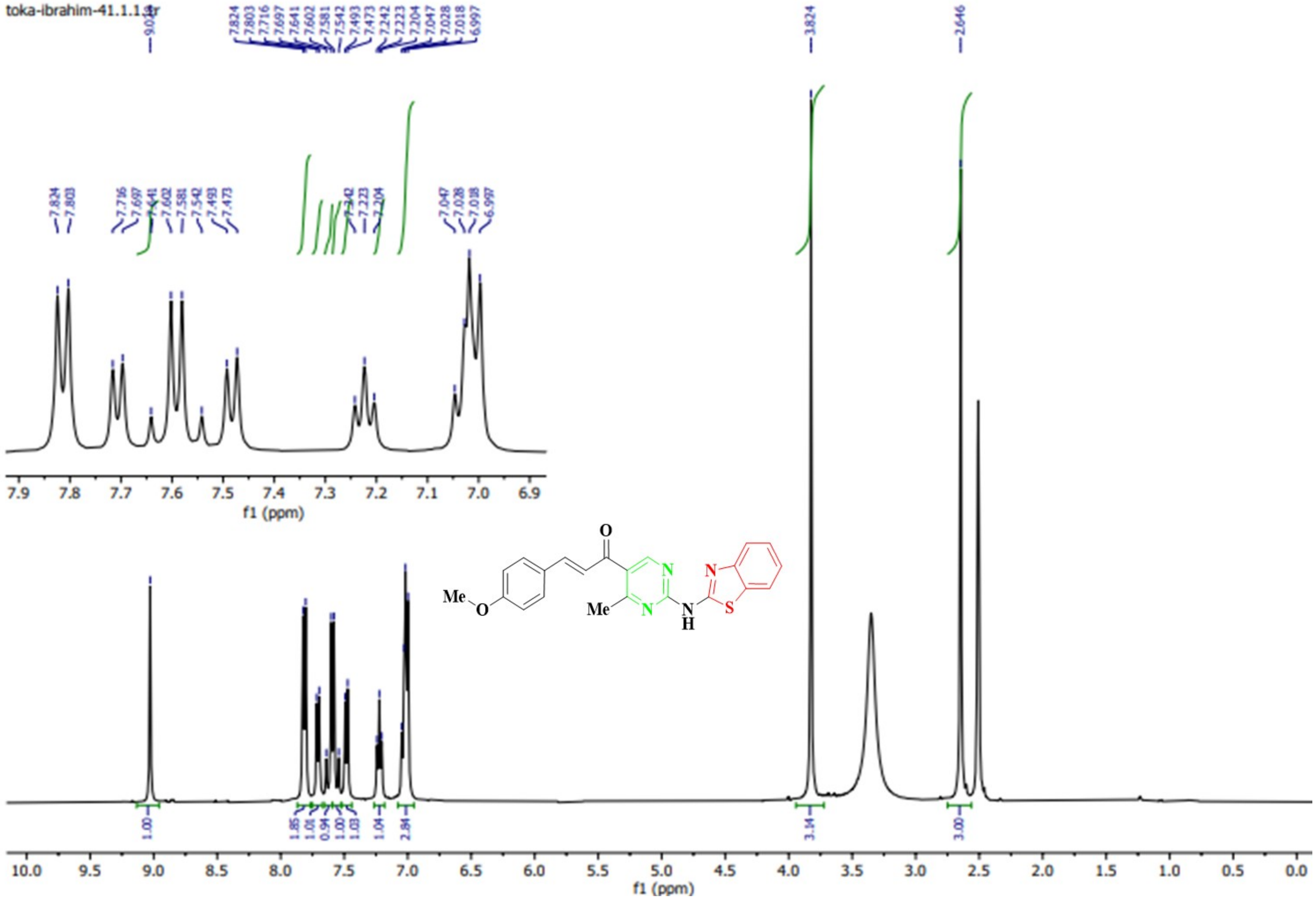

Figure 34. ^{1}H NMR spectrum of compound **17d**

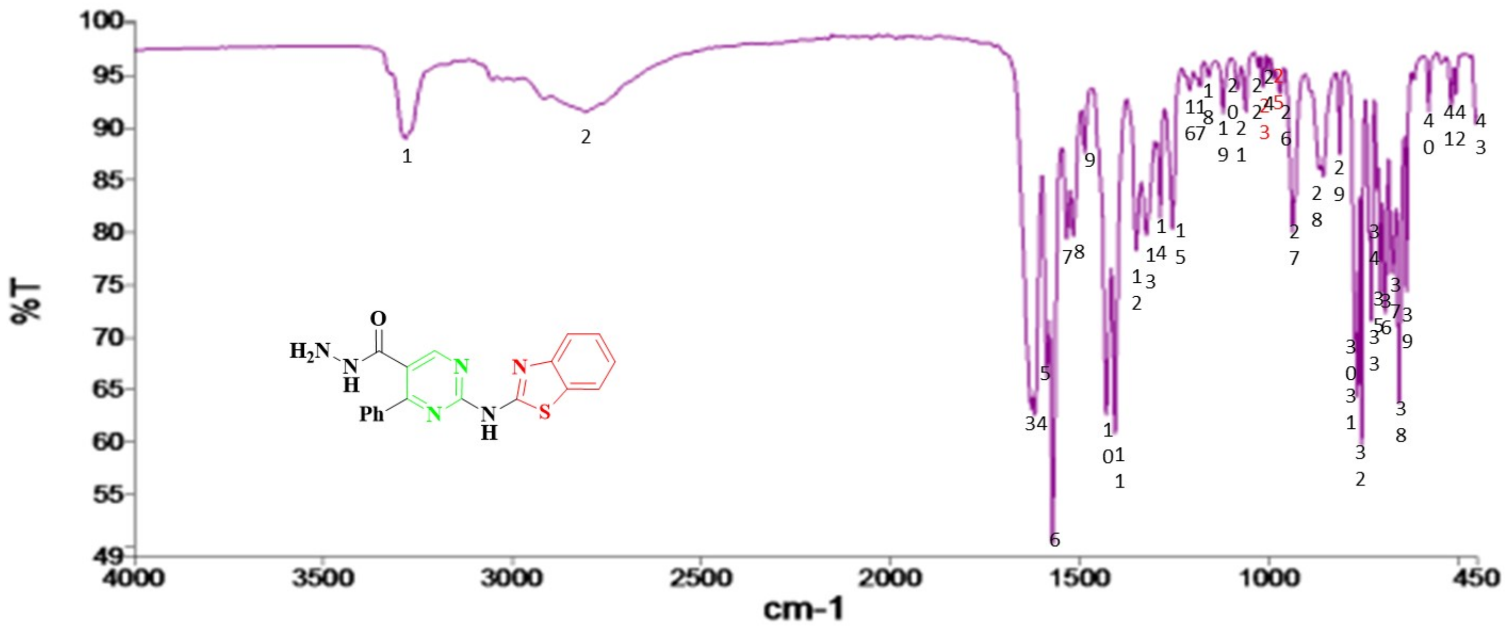

No.	Position	Intinsty	No.	Position	Intinsty	No.	Position	Intinsty	No.	Position	Intinsty	No.	Position	Intinsty
1	3285.18	89.06	10	1429.30	62.55	19	1120.81	91.43	28	854.88	85.40	37	671.69	76.11
2	2807.00	91.63	11	1406.68	60.75	20	1081.26	93.78	29	811.62	87.52	38	653.97	63.60
3	1628.77	63.02	12	1350.21	78.36	21	1060.13	91.63	30	771.91	69.77	39	634.57	74.38
4	1618.23	62.52	13	1323.61	79.80	22	1027.48	95.91	31	765.73	64.32	40	575.75	91.67
5	1586.06	66.14	14	1286.78	81.46	23	1014.65	94.00	32	753.58	59.65	41	516.88	91.93
6	1571.16	50.22	15	1254.43	80.42	24	1001.13	95.29	33	727.87	71.57	42	504.01	93.30
7	1534.08	79.44	16	1208.80	93.73	25	987.67	94.75	34	712.78	84.06	43	451.84	90.50
8	1517.07	79.75	17	1182.14	94.05	26	969.17	93.35	35	703.94	74.23			
9	1486.71	87.68	18	1158.03	94.92	27	936.12	80.07	36	690.84	72.30			

Figure 35. IR spectrum of compound **18**

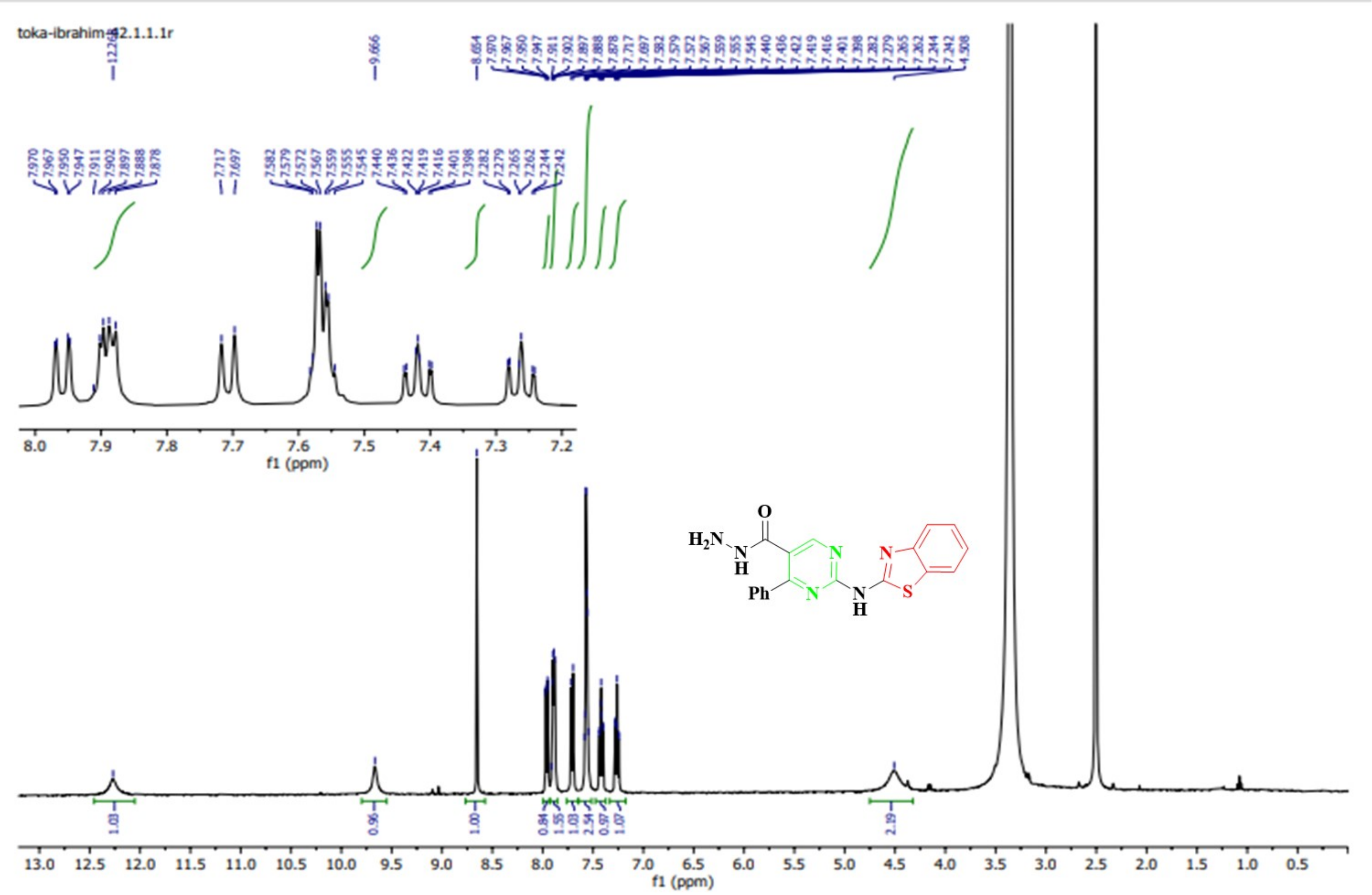

Figure 36. ^{1}H NMR spectrum of compound **18**

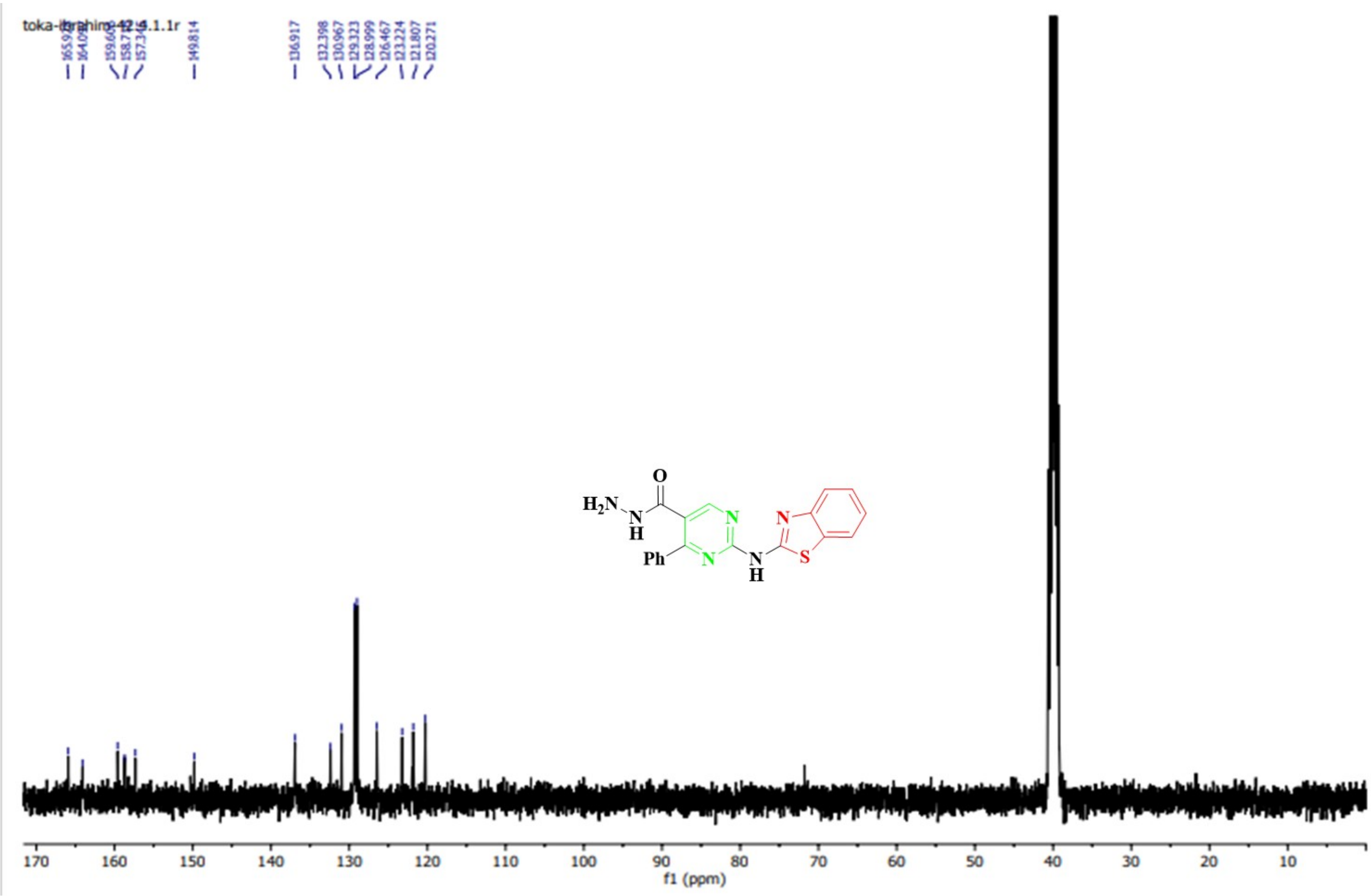

Figure 37. ^{13}C NMR spectrum of compound **18**

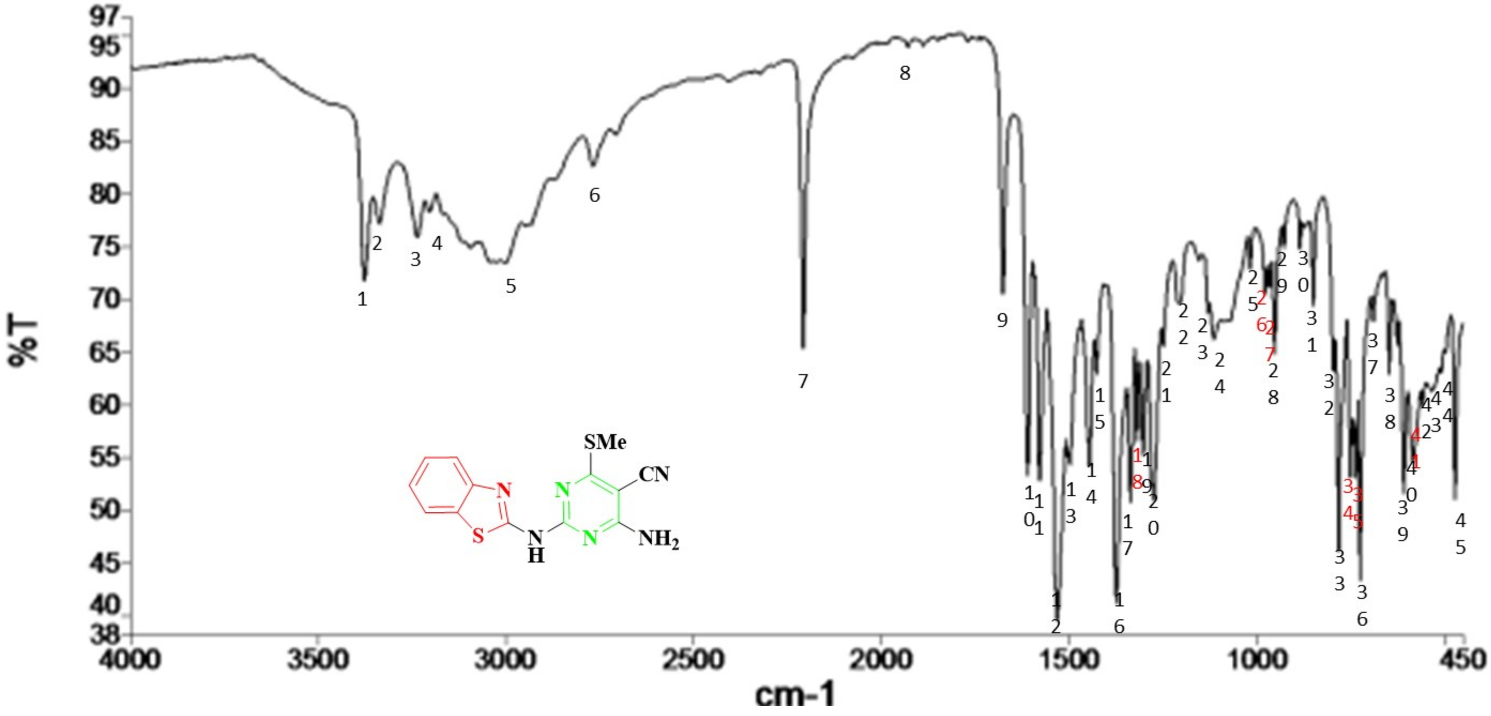

No.	Position	Intinsty	No.	Position	Intinsty	No.	Position	Intinsty	No.	Position	Intinsty	No.	Position	Intinsty
1	3379.48	71.84	10	1610.16	53.29	19	1302.24	55.17	28	951.69	64.88	37	688.24	68.01
2	3339.59	77.23	11	1578.68	52.76	20	1272.96	50.88	29	926.70	74.95	38	645.92	62.98
3	3238.48	75.96	12	1531.64	39.54	21	1248.89	65.60	30	884.56	74.92	39	624.26	65.61
4	3205.87	78.40	13	1497.18	54.33	22	1204.62	69.64	31	848.46	69.43	40	606.91	51.58
5	3004.34	73.45	14	1445.56	54.16	23	1130.07	68.74	32	795.32	63.25	41	582.75	54.56
6	2769.92	82.70	15	1426.44	62.90	24	1113.66	66.25	33	780.85	46.08	42	556.78	59.98
7	2208.92	65.36	16	1373.81	41.13	25	1017.38	72.95	34	749.98	53.06	43	533.27	61.31
8	1930.31	94.09	17	1336.64	50.72	26	976.92	70.60	35	734.28	53.07	44	507.99	62.98
9	1676.17	70.59	18	1318.02	56.60	27	964.43	71.17	36	723.90	43.26	45	470.42	51.00

Figure 38. IR spectrum of compound **20**

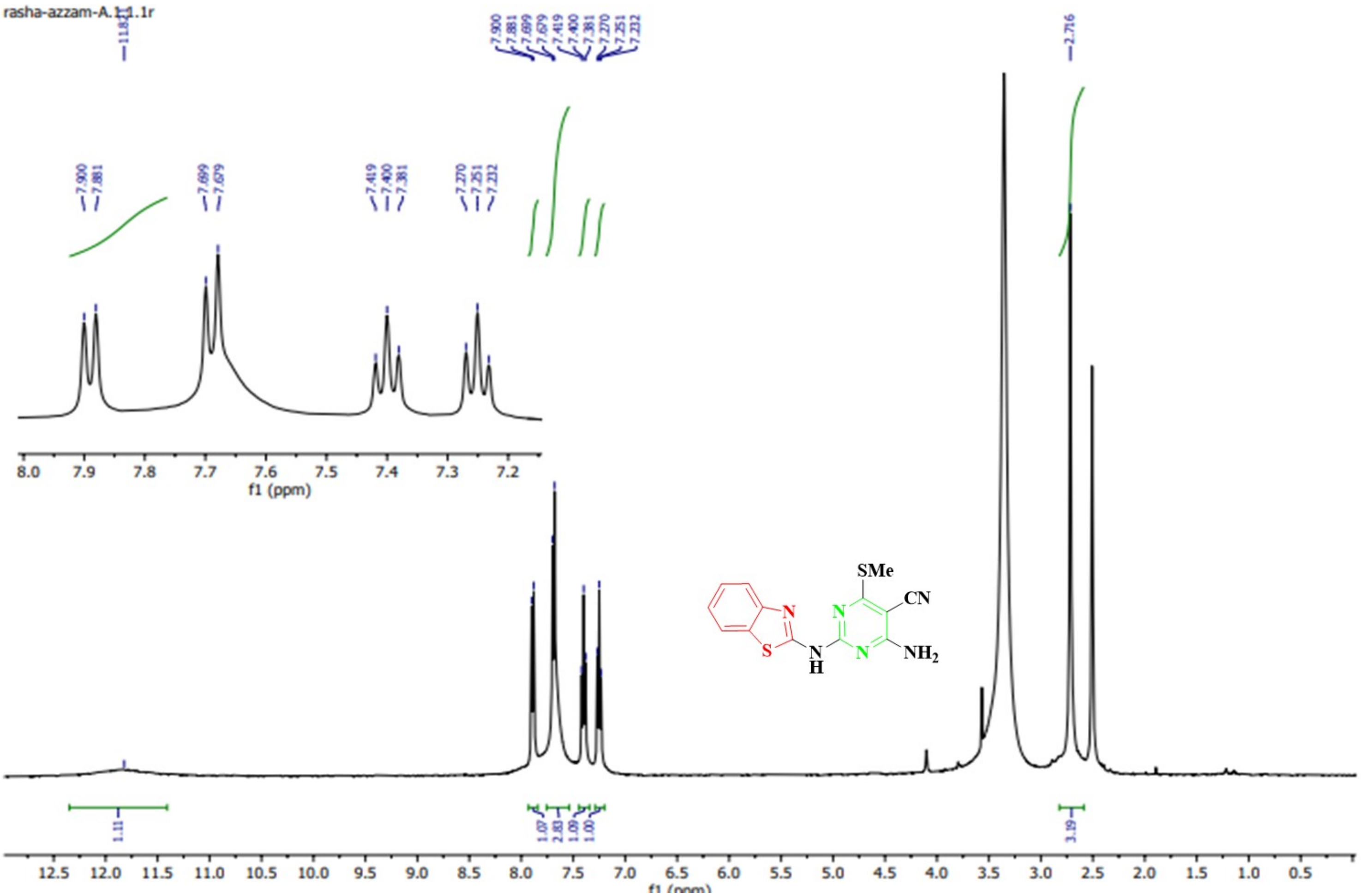

Figure 39. ^{1}H NMR spectrum of compound **20**

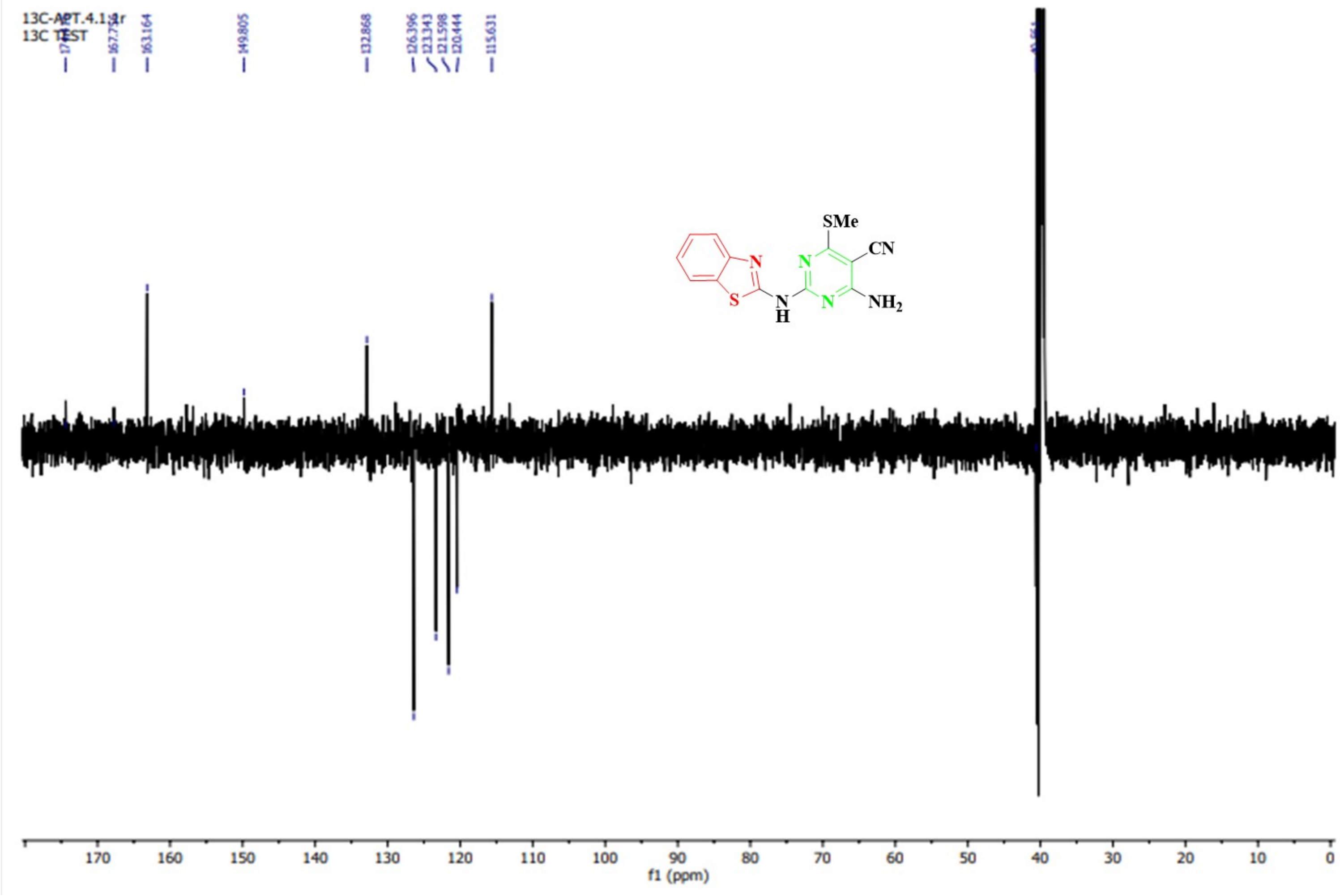

Figure 40. ^{13}C NMR spectrum of compound **20**

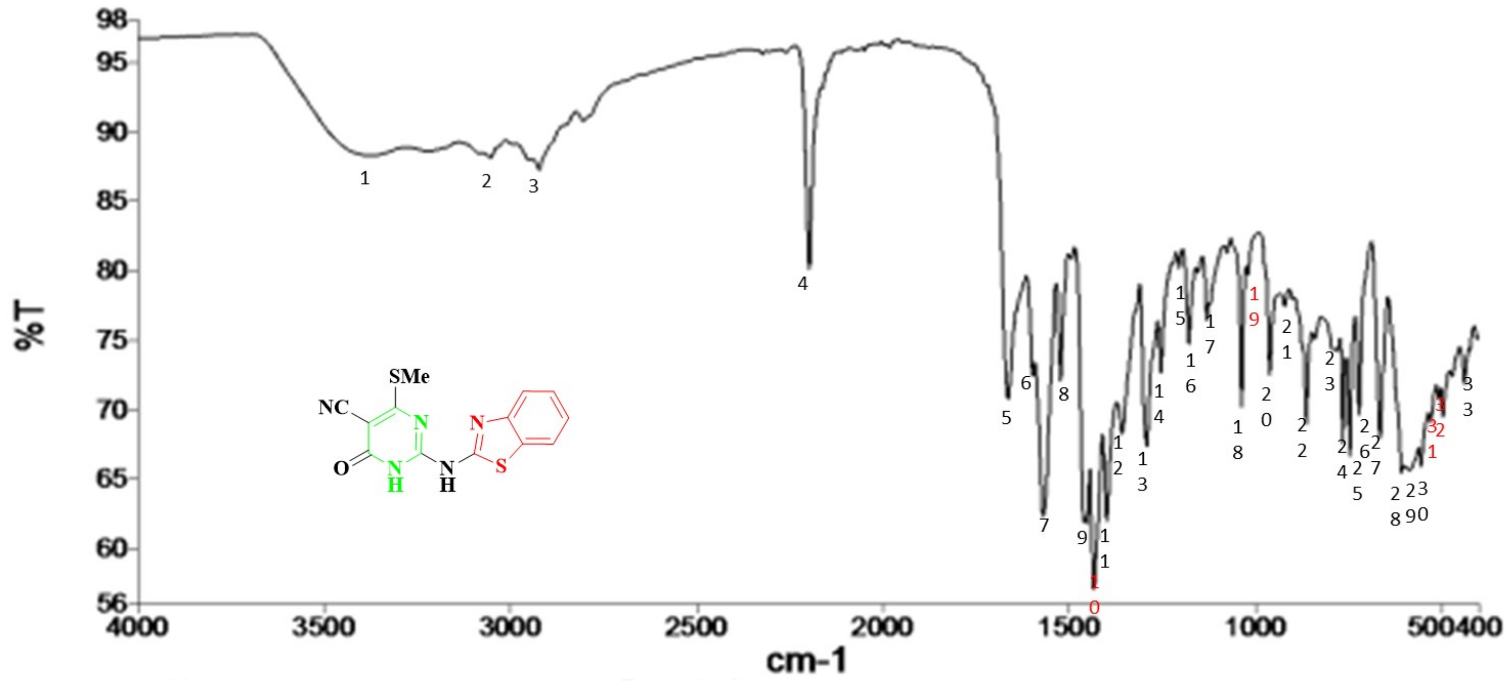

No.	Position	Intensity	No.	Position	Intensity	No.	Position	Intensity	No.	Position	Intensity	No.	Position	Intensity
1	3378.59	88.35	8	1523.08	72.09	15	1204.85	80.24	22	862.29	68.99	29	554.11	65.90
2	3055.36	88.18	9	1452.50	61.76	16	1178.16	74.76	23	778.43	74.20	30	527.95	69.11
3	2924.68	87.32	10	1433.83	57.02	17	1129.62	76.50	24	764.23	67.59	31	506.92	70.86
4	2198.45	80.17	11	1398.95	61.93	18	1036.59	70.17	25	745.08	66.66	32	495.01	69.49
5	1663.77	70.81	12	1357.68	68.28	19	1019.42	79.76	26	720.50	69.6	33	436.99	71.84
6	1597.67	72.47	13	1290.69	67.35	20	960.81	72.50	27	663.90	67.90			
7	1569.66	62.32	14	1253.44	72.72	21	920.40	77.49	28	606.72	65.46			

Figure 41. IR spectrum of compound **22**

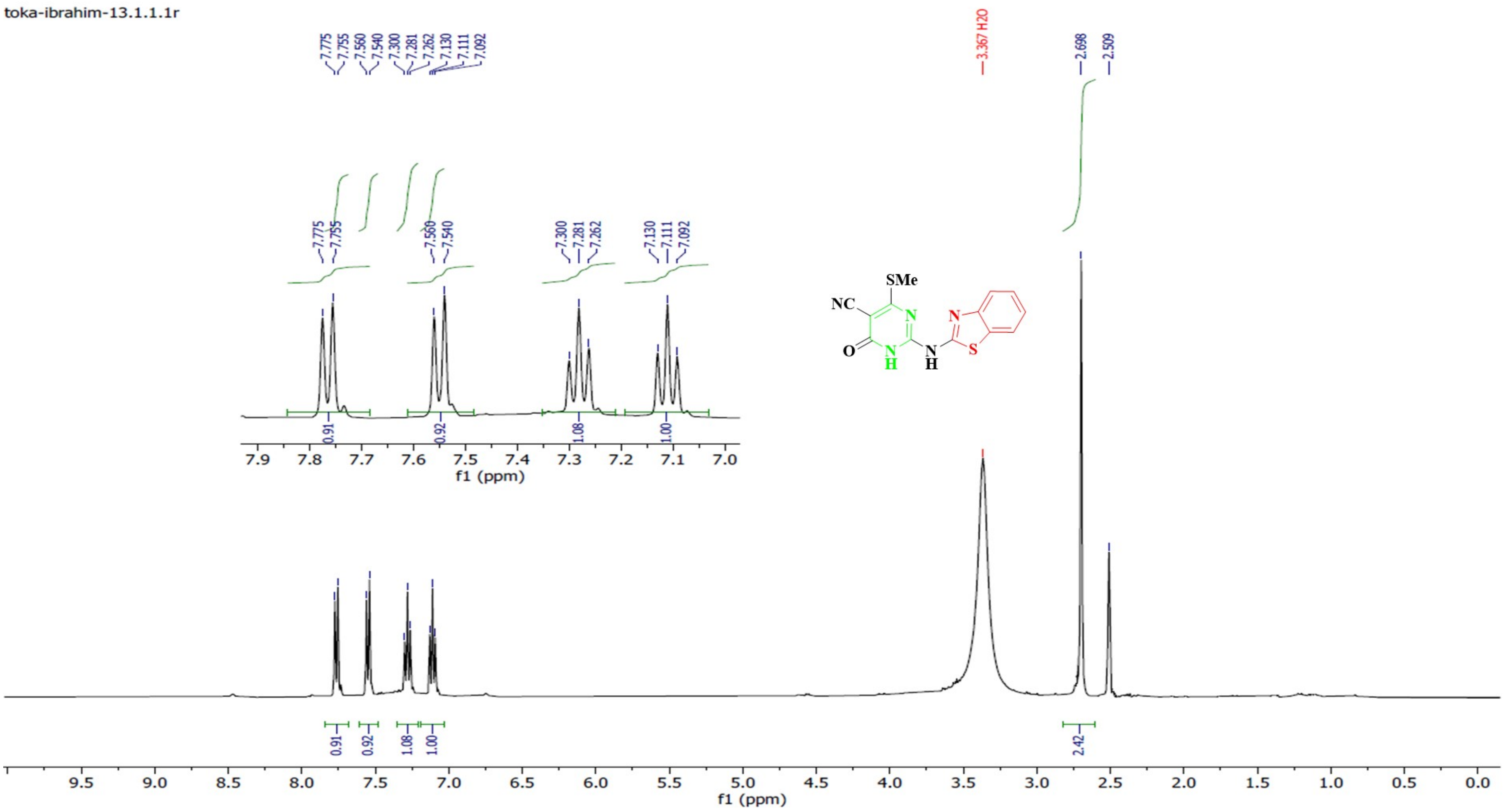

Figure 42. ^{1}H NMR spectrum of compound **22**

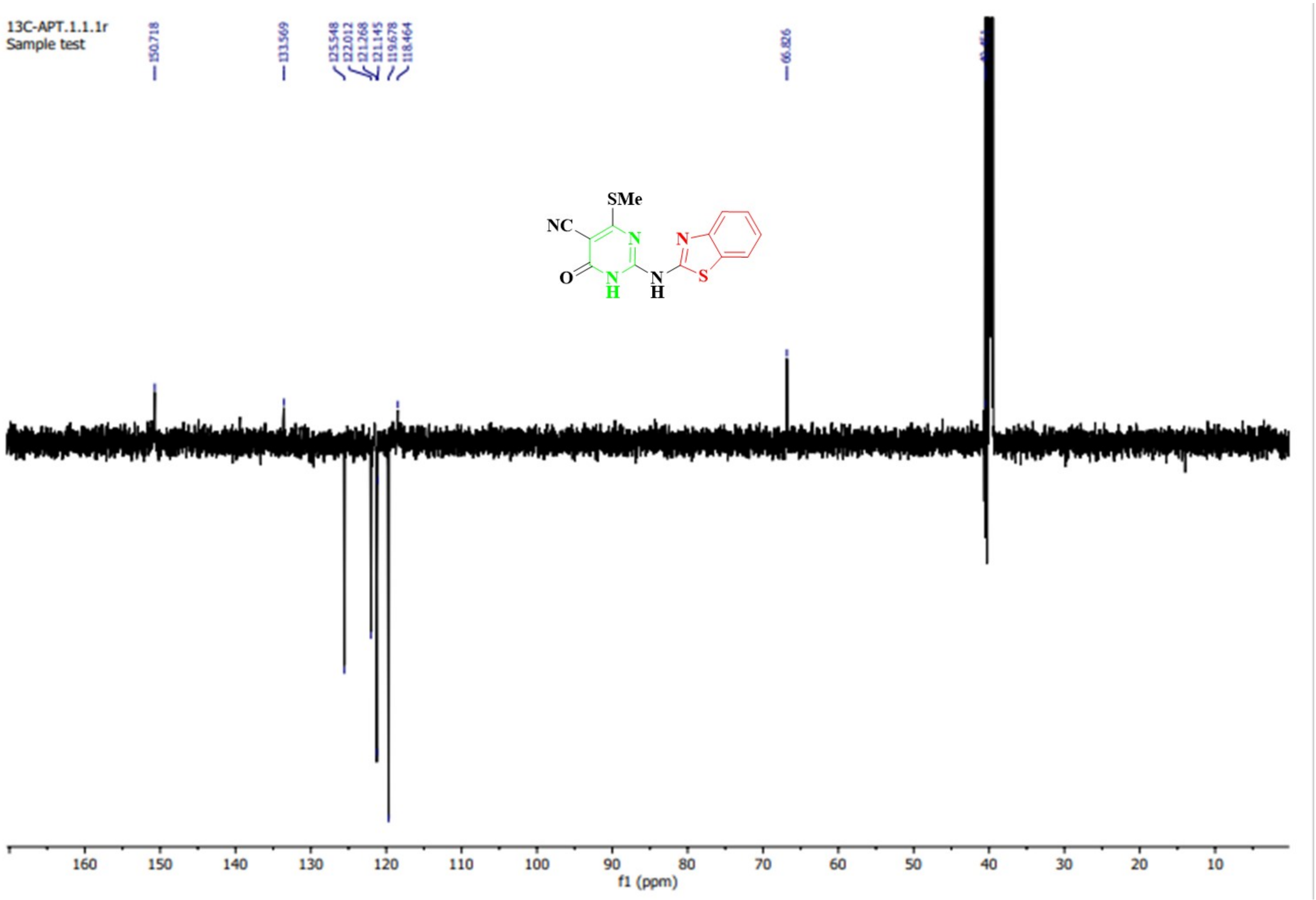

Figure 43. ^{13}C NMR spectrum of compound **22**

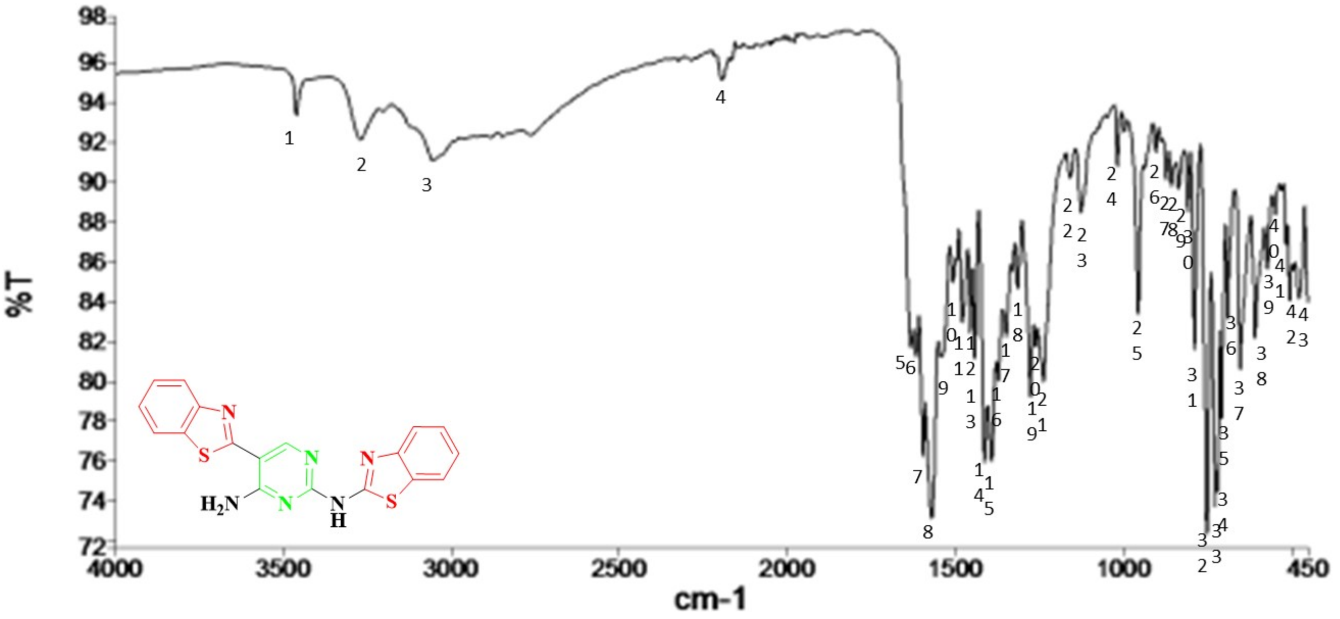

No.	Position	Intinsty	No.	Position	Intinsty	No.	Position	Intinsty	No.	Position	Intinsty	No.	Position	Intinsty
1	3464.19	93.40	10	1505.92	84.94	19	1276.52	79.22	28	855.46	89.84	37	650.94	80.61
2	3273.37	92.18	11	1478.66	82.95	20	1260.07	81.76	29	834.36	89.69	38	606.72	82.17
3	3057.47	91.13	12	1455.23	82.43	21	1236.84	79.97	30	807.29	88.52	39	570.20	85.66
4	2195.74	95.21	13	1441.88	81.15	22	1157.67	90.27	31	786.91	81.58	40	546.30	88.40
5	1632.17	81.70	14	1412.50	75.89	23	1124.61	88.51	32	750.88	72.27	41	514.52	86.88
6	1616.70	81.18	15	1391.72	75.96	24	1016.89	90.84	33	726.03	73.65	42	503.29	84.07
7	1595.34	76.18	16	1372.08	80.05	25	955.15	83.37	34	718.45	74.45	43	475.06	84.16
8	1570.32	73.07	17	1348.56	82.26	26	902.08	91.55	35	706.43	78.11			
9	1542.97	81.21	18	1314.59	84.67	27	871.74	90.16	36	687.96	83.13			

Figure 44. IR spectrum of compound 24

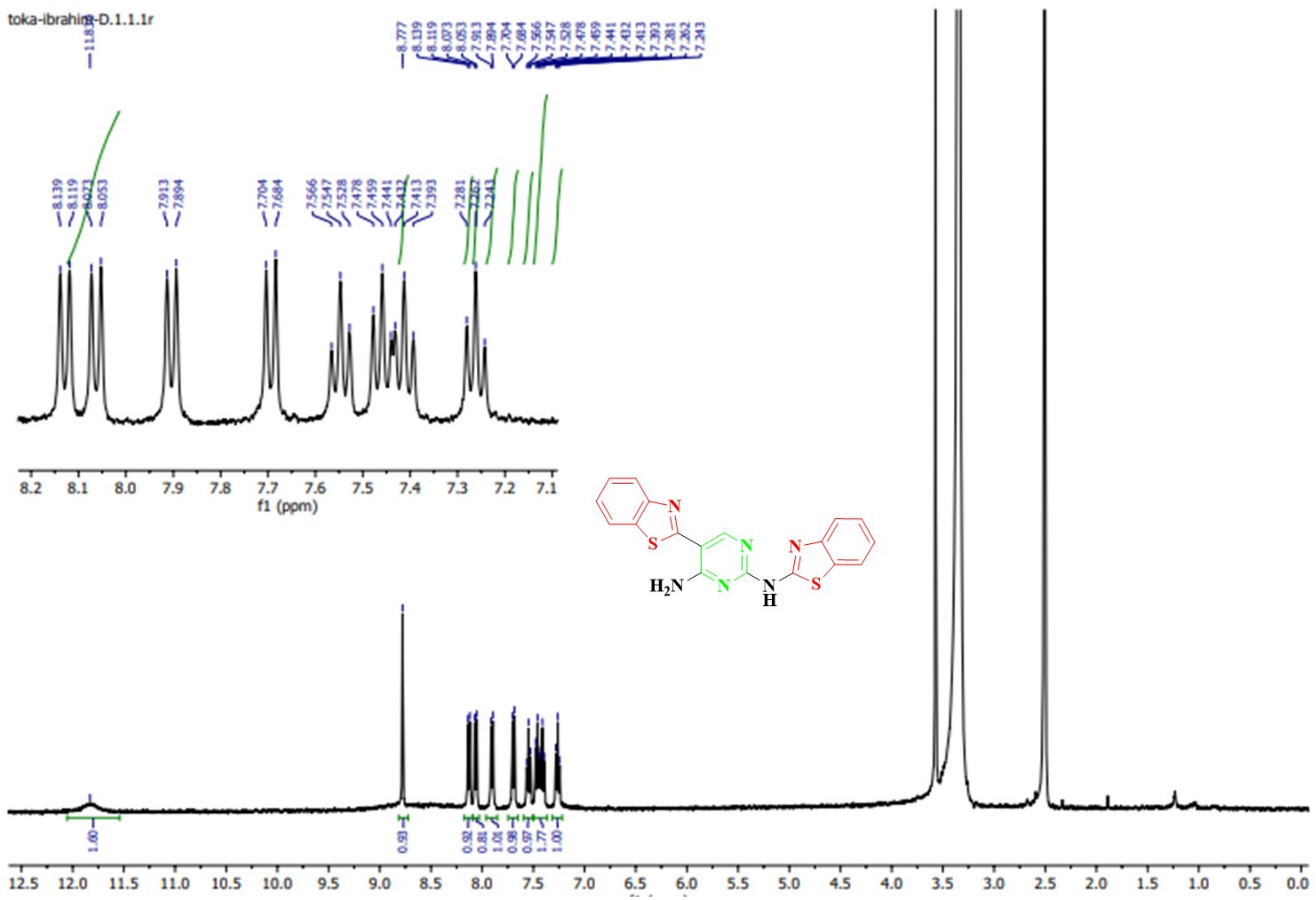

Figure 45. ^{1}H NMR spectrum of compound **24**

Assessment of cytotoxic effect of different pyrimidine derivatives on cancer cell lines:

Table 1. MTT assay results for 10 formulas in HepG2 cancer cells.

Experiment	HepG2	7a	7b	7c	7d	13a	13b	13c	15a	15b	15c	5-FU
**OD1**	3.518	3.173	2.418	2.111	3.273	2.658	2.479	2.934	2.863	3.381	2.572	**2.256**
**OD2**	3.328	2.863	2.574	2.052	3.381	2.514	2.315	2.913	2.719	3.205	2.522	**2.082**
**OD3**	3.482	2.815	2.522	2.171	3.219	2.305	2.252	2.822	2.806	3.228	2.419	**2.319**
**Average OD**	3.443	2.950	2.505	2.111	3.291	2.492	2.349	2.890	2.796	3.271	2.504	**2.219**
**Viability (%) 1**	102.18	92.16	70.23	61.31	95.06	77.20	72.00	85.22	83.15	98.20	74.70	**65.52**
**Viability (%) 2**	96.66	83.15	74.76	59.60	98.20	73.02	67.24	84.61	78.97	93.09	73.25	**60.47**
**Viability (%) 3**	101.13	81.76	73.25	63.06	93.4	66.95	65.41	81.96	81.50	93.76	70.26	**67.35**
**Viability (%)**	99.93	85.64	72.70	61.29	95.53	72.35	68.18	83.88	81.16	94.96	72.69	**64.41**

OD: optic density. MTT: 4,5-dimethylthiazol-2-yl)-2,5-diphenyltetrazolium bromide

Table 2. MTT assay results for 10 formulas in HepG2 cancer cells.

Experiment	HepG2	17a	17b	17c	17d	18	20	22	24	5-FU
**OD1**	3.518	2.934	2.658	2.765	2.111	2.479	3.385	2.866	2.765	**2.256**
**OD2**	3.328	2.741	2.571	2.811	2.052	2.315	3.272	2.716	2.711	**2.082**
**OD3**	3.482	2.836	2.628	2.739	2.145	2.115	3.162	2.86	2.628	**2.319**
**Average OD**	3.443	2.837	2.619	2.772	2.103	2.303	3.273	2.814	2.701	**2.219**
**Viability (%) 1**	102.18	85.22	77.20	80.31	61.31	72.00	98.32	83.24	80.31	**65.52**
**Viability (%) 2**	96.66	79.61	74.67	81.64	59.60	67.24	95.03	78.88	78.74	**60.47**
**Viability (%) 3**	101.13	82.37	76.33	79.55	62.30	61.43	91.84	83.07	76.33	**67.35**
**Viability (%)**	99.93	82.35	76.02	80.45	61.04	66.85	95.01	81.68	78.41	**64.41**

OD: optic density. MTT: 4,5-dimethylthiazol-2-yl)-2,5-diphenyltetrazolium bromide

Table 3. MTT assay results for 10 formulas in HCT116 cancer cells.

Experiment	HCT 116	7a	7b	7c	7d	13a	13b	13c	15a	15b	15c	5-FU
**OD1**	3.571	2.807	2.463	3.169	3.192	2.724	3.322	2.412	3.045	3.192	2.463	**2.018**
**OD2**	3.519	2.719	2.596	3.064	3.381	2.615	3.126	2.358	3.182	3.092	2.319	**2.022**
**OD3**	3.622	2.522	2.507	3.115	3.211	2.552	3.052	2.419	3.055	3.117	2.254	**1.955**
**Average OD**	3.571	2.683	2.522	3.116	3.261	2.630	3.167	2.396	3.094	3.134	2.345	**1.998**
**Viability (%) 1**	100.00	78.61	68.97	88.74	89.39	76.28	93.03	67.5	85.27	89.39	68.97	**56.51**
**Viability (%) 2**	98.54	76.14	72.70	85.80	94.68	73.23	87.54	66.03	89.11	86.59	64.94	**56.62**
**Viability (%) 3**	101.43	70.62	70.20	87.23	89.92	71.46	85.47	67.74	85.55	87.29	63.12	**54.75**
**Viability (%)**	99.99	75.12	70.62	87.26	91.33	73.66	88.68	67.11	86.64	87.75	65.68	**55.96**

OD: optic density. MTT: 4,5-dimethylthiazol-2-yl)-2,5-diphenyltetrazolium bromide

Table 4. MTT assay results for 10 formulas in HCT116 cancer cells.

Experiment	HCT 116	17a	17b	17c	17d	18	20	22	24	5-FU
**OD1**	3.571	3.066	3.155	3.069	3.168	3.322	3.019	3.195	3.229	**2.018**
**OD2**	3.519	2.952	3.251	3.115	3.056	3.192	3.105	3.092	3.069	**2.022**
**OD3**	3.622	3.185	3.185	3.018	3.112	3.257	2.951	3.172	3.112	**1.955**
**Average OD**	3.571	3.068	3.197	3.067	3.112	3.257	3.025	3.153	3.137	**1.998**
**Viability (%) 1**	100.00	85.86	88.35	85.94	88.71	93.03	84.54	89.47	90.42	**56.51**
**Viability (%) 2**	98.54	82.67	91.04	87.23	85.58	89.39	86.95	86.59	85.94	**56.62**
**Viability (%) 3**	101.43	89.19	89.19	84.51	87.15	91.21	82.64	88.83	87.15	**54.75**
**Viability (%)**	99.99	85.90	89.53	85.90	87.15	91.21	84.71	88.29	87.84	**55.96**

OD: optic density. MTT: 4,5-dimethylthiazol-2-yl)-2,5-diphenyltetrazolium bromide

Table 5. MTT assay results for 10 formulas in MCF7 cancer cells.

Experiment	MCF7	7a	7b	7c	7d	13a	13b	13c	15a	15b	15c	5-FU
**OD1**	3.062	2.948	3.365	3.157	2.974	2.452	2.762	2.619	3.271	3.126	3.061	**2.152**
**OD2**	3.152	2.715	3.061	3.348	3.126	2.319	2.715	2.974	3.182	3.085	3.115	**1.928**
**OD3**	3.181	2.814	2.912	3.116	2.881	2.224	3.046	2.655	3.055	2.954	3.092	**1.817**
**Average OD**	3.132	2.826	3.113	3.207	2.994	2.332	2.841	2.749	3.169	3.055	3.089	**1.966**
**Viability (%) 1**	97.77	94.13	107.44	100.80	94.96	78.29	88.19	83.62	104.44	99.81	97.73	**68.71**
**Viability (%) 2**	100.64	86.69	97.73	106.90	99.81	74.04	86.69	94.96	101.60	98.50	99.46	**61.56**
**Viability (%) 3**	101.56	89.85	92.98	99.49	91.99	71.01	97.25	84.77	97.54	94.32	98.72	**58.01**
**Viability (%)**	99.99	90.22	99.38	102.39	95.58	74.45	90.71	87.78	101.19	97.54	98.64	**62.76**

OD: optic density. MTT: 4,5-dimethylthiazol-2-yl)-2,5-diphenyltetrazolium bromide

Table 6. MTT assay results for 10 formulas in MCF7 cancer cells.

Experiment	MCF7	17a	17b	17c	17d	18	20	22	24	5-FU
**OD1**	3.062	2.974	3.224	3.242	3.157	3.046	2.671	3.252	2.151	**2.152**
**OD2**	3.152	2.718	3.192	3.152	3.152	2.819	2.527	3.183	2.242	**1.928**
**OD3**	3.181	2.622	3.185	3.005	3.055	3.072	2.711	2.951	2.182	**1.817**
**Average OD**	3.132	2.771	3.200	3.133	3.121	2.979	2.636	3.129	2.192	**1.966**
**Viability (%) 1**	97.77	94.96	102.9	103.51	100.8	97.25	85.28	103.83	68.68	**68.71**
**Viability (%) 2**	100.64	86.78	101.9	100.64	100.6	90.01	80.68	101.63	71.58	**61.56**
**Viability (%) 3**	101.56	83.72	101.6	95.95	97.54	98.08	86.56	94.22	69.67	**58.01**
**Viability (%)**	99.99	88.48	102.2	100.03	99.66	95.11	84.17	99.89	69.98	**62.76**

OD: optic density. MTT: 4,5-dimethylthiazol-2-yl)-2,5-diphenyltetrazolium bromide

2. Assessment of IC_{50} of the selected compounds:

Table 7. Determination of IC_{50} of compound 7c on HepG2 cells.

Experiment	IC_{50} (µmol/mL)					
	**HepG2**	**0.01**	**0.1**	**1**	**10**	**100**
**OD1**	2.989	2.806	2.650	2.358	1.924	1.484
**OD2**	2.774	2.644	2.592	2.311	1.866	1.448
**OD3**	2.804	2.782	2.633	2.396	1.879	1.547
**Average OD**	2.856	2.744	2.625	2.355	1.890	1.493
**Viability (%) 1**	104.65%	98.24%	92.78%	82.56%	67.37%	51.96%
**Viability (%) 2**	97.14%	92.59%	90.74%	80.91%	65.35%	50.69%
**Viability (%) 3**	98.17%	97.41%	92.21%	83.88%	65.79%	54.15%
**Viability (%)**	100.90%	95.42%	91.76%	81.74%	66.36%	51.32%
**IC_{50}**	**2.73±0.25 µmol/mL**					

OD: optic density. MTT: 4,5-dimethylthiazol-2-yl)-2,5-diphenyltetrazolium bromide

Table 8. Determination of IC_{50} of compound 13b on HepG2 cells.

Experiment	IC_{50} (µmol/mL)					
	**HepG2**	**0.01**	**0.1**	**1**	**10**	**100**
**OD1**	2.989	2.467	2.315	2.146	1.868	1.583
**OD2**	2.774	2.558	2.276	2.139	1.866	1.733
**OD3**	2.804	2.501	2.413	2.126	1.996	1.701
**Average OD**	2.856	2.508	2.335	2.137	1.910	1.673
**Viability (%) 1**	104.65%	86.37%	81.07%	75.15%	65.40%	55.44%
**Viability (%) 2**	97.14%	89.56%	79.71%	74.88%	65.33%	60.67%
**Viability (%) 3**	98.17%	87.56%	84.50%	74.43%	69.87%	59.57%
**Viability (%)**	100.90%	87.97%	80.39%	75.02%	65.36%	58.06%
**IC_{50}**	**0.56±0.03 µmol/mL**					

OD: optic density. MTT: 4,5-dimethylthiazol-2-yl)-2,5-diphenyltetrazolium bromide

Table 9. Determination of IC_{50} of compound 17d on HepG2 cells.

Experiment	IC_{50} (µmol/mL)					
	**HepG2**	**0.01**	**0.1**	**1**	**10**	**100**
**OD1**	2.989	2.589	2.430	2.051	1.801	1.746
**OD2**	2.774	2.569	2.345	2.080	1.870	1.718
**OD3**	2.804	2.476	2.469	2.060	1.762	1.718
**Average OD**	2.856	2.545	2.414	2.064	1.811	1.727
**Viability (%) 1**	101.59%	88.00%	82.58%	69.72%	61.21%	59.34%
**Viability (%) 2**	94.30%	87.32%	79.70%	70.71%	63.56%	58.40%
**Viability (%) 3**	95.33%	84.17%	83.95%	70.04%	59.90%	58.41%
**Viability (%)**	97.95%	87.66%	81.14%	70.22%	62.38%	58.87%
**IC_{50}**	**0.41 ±0.01 µmol/mL**					

OD: optic density. MTT: 4,5-dimethylthiazol-2-yl)-2,5-diphenyltetrazolium bromide

Table 10. Determination of IC_{50} of compound 18 on HepG2 cells.

Experiment	IC_{50} (µmol/mL)					
	**HepG2**	**0.01**	**0.1**	**1**	**10**	**100**
**OD1**	2.989	2.608	2.443	2.144	1.730	1.673
**OD2**	2.774	2.622	2.462	2.082	1.715	1.671
**OD3**	2.804	2.653	2.446	2.063	1.700	1.789
**Average OD**	2.856	2.628	2.450	2.096	1.715	1.711
**Viability (%) 1**	101.59%	91.31%	85.55%	75.07%	60.56%	58.58%
**Viability (%) 2**	94.30%	91.82%	86.21%	72.89%	60.06%	58.51%
**Viability (%) 3**	95.33%	92.88%	85.64%	72.22%	59.53%	62.64%
**Viability (%)**	97.95%	91.57%	85.88%	73.98%	60.31%	58.54%
**IC_{50}**	**0.53 ±0.05 µmol/mL**					

OD: optic density. MTT: 4,5-dimethylthiazol-2-yl)-2,5-diphenyltetrazolium bromide

Table 11. Determination of IC_{50} of compound 7b on HCT116cells.

Experiment	IC_{50} (µmol/mL)					
	HCT116	0.01	0.1	1	10	100
OD1	2.768	2.595	2.557	2.375	2.211	2.02
OD2	2.704	2.613	2.583	2.406	2.107	1.852
OD3	2.606	2.652	2.566	2.491	2.119	1.931
Average OD	2.693	2.620	2.569	2.424	2.146	1.934
Viability (%) 1	102.78%	96.36%	94.95%	88.19%	82.10%	75.01%
Viability (%) 2	100.41%	97.03%	95.92%	89.34%	78.24%	68.77%
Viability (%) 3	96.77%	98.48%	95.28%	92.50%	78.69%	71.70%
Viability (%)	101.60%	96.70%	95.43%	88.77%	80.17%	71.89%
IC_{50}	2.95±0.26 µmol/mL					

OD: optic density. MTT: 4,5-dimethylthiazol-2-yl)-2,5-diphenyltetrazolium bromide

Table 12. Determination of IC_{50} of compound 13c on HCT116cells.

Experiment	IC_{50} (µmol/mL)					
	HCT116	0.01	0.1	1	10	100
OD1	2.768	2.649	2.587	2.271	2.154	1.822
OD2	2.704	2.621	2.572	2.261	2.107	1.917
OD3	2.606	2.575	2.485	2.309	2.155	1.862
Average OD	2.693	2.615	2.548	2.280	2.139	1.867
Viability (%) 1	102.78%	98.37%	96.06%	84.33%	79.99%	67.66%
Viability (%) 2	100.41%	97.33%	95.51%	83.96%	78.24%	71.18%
Viability (%) 3	96.77%	95.62%	92.28%	85.74%	80.02%	69.14%
Viability (%)	101.60%	97.85%	95.79%	84.14%	79.11%	69.42%
IC_{50}	1.033±0.06 µmol/mL					

OD: optic density. MTT: 4,5-dimethylthiazol-2-yl)-2,5-diphenyltetrazolium bromide

Table 13. Determination of IC_{50} of compound 15c on HCT116cells.

Experiment	IC_{50} (µmol/mL)					
	HCT116	0.01	0.1	1	10	100
OD1	2.768	2.284	1.416	1.258	1.179	1.041
OD2	2.704	2.296	1.466	1.311	1.134	1.069
OD3	2.606	2.062	1.396	1.230	1.112	1.038
Average OD	2.693	2.214	1.426	1.267	1.142	1.049
Viability (%) 1	102.78%	84.81%	52.58%	46.72%	43.78%	38.67%
Viability (%) 2	100.41%	85.26%	54.44%	48.69%	42.11%	39.68%
Viability (%) 3	96.77%	76.57%	51.84%	45.68%	41.29%	38.56%
Viability (%)	101.60%	85.04%	53.51%	47.71%	42.95%	39.18%
IC_{50}	0.02±0.001 µmol/mL					

OD: optic density. MTT: 4,5-dimethylthiazol-2-yl)-2,5-diphenyltetrazolium bromide

Table 14. Determination of IC_{50} of compound 24 on MCF7 cells.

Experiment	IC_{50} (µmol/mL)					
	MCF7	0.01	0.1	1	10	100
OD1	2.869	2.790	2.654	2.362	2.172	1.943
OD2	2.859	2.774	2.716	2.461	2.043	1.845
OD3	2.736	2.780	2.719	2.450	2.128	1.913
Average OD	2.821	2.781	2.697	2.424	2.114	1.900
Viability (%) 1	100.73%	97.95%	93.18%	82.94%	76.27%	68.23%
Viability (%) 2	100.38%	97.39%	95.38%	86.40%	71.72%	64.77%
Viability (%) 3	96.06%	97.62%	95.48%	86.02%	74.73%	67.18%
Viability (%)	100.55%	97.67%	94.28%	84.67%	74.00%	66.50%
IC_{50}	1.485±0.15 µmol/mL					

OD: optic density. MTT: 4,5-dimethylthiazol-2-yl)-2,5-diphenyltetrazolium bromide
